# A Metacognitive Intervention for Children and Adolescents in Neuropediatric Care (Mio-Training): Protocol for a Randomized Controlled Trial

**DOI:** 10.2196/95139

**Published:** 2026-06-26

**Authors:** Saskia Salzmann, Valentin Benzing, Sebastian Grunt, Rhoikos Furtwängler, Regula Everts

**Affiliations:** 1 Division of Neuropaediatrics, Development and Rehabilitation, Department of Paediatrics, Inselspital, Bern University Hospital, University of Bern, Freiburgstrasse 15Bern, 3010, Switzerland, +41 316328497; 2 Graduate School for Health Sciences, University of Bern, Switzerland; 3 Institute of Sport Science, University of Bern, Bern, Switzerland; 4 Division of Pediatric Hematology and Oncology, Department of Pediatrics, Inselspital, Bern University Hospital, University of Bern, Switzerland

**Keywords:** cognitive training, metacognitive abilities, childhood, pediatric cancer, attention-deficit/hyperactivity disorder, ADHD, mnemonic strategies, motor coordination, working memory training

## Abstract

**Background:**

Diseases during childhood and adolescence such as cancer or attention-deficit/hyperactivity disorder (ADHD) can have an impact on brain development and place children and adolescents at increased risk for cognitive long-term problems. Most cognitive trainings currently available have limited efficacy and show limited transfer to nontrained tasks and everyday functioning. We developed a novel intervention (Mio-Training) aiming to increase metacognitive abilities at the intersection between exercise psychology and cognitive science to strengthen the cognitive development of pediatric patients with atypical brain development in the long term.

**Objective:**

The study assesses the efficacy of the Mio-Training on the primary (metacognitive abilities) and secondary outcomes (executive functions, processing speed, and memory) before the training, immediately after the training, and at a 3-month follow-up in patients with atypical development and healthy controls.

**Methods:**

The Mio-Training stimulates metacognition through 38 digital games, which playfully teach mnemonic strategies (ie, rehearsal, chaining, and associations), present intensive verbal and visual working memory training, and motor coordination tasks. The training group will train for 5 weeks, 3 times per week, for 20 minutes. The waiting control group will receive the training after completion of the study procedure. We will evaluate the efficacy of the Mio-Training on metacognitive abilities and cognitive performance in a randomized controlled clinical trial. We expect a long-term increase in metacognitive abilities associated with an increase in subjective and objective cognitive performance. The efficacy of the Mio-Training will be investigated in 3 subgroups (patients with cancer, ADHD, and healthy controls; each group n=40; all aged 8‐16 years) using pre-intervention and post-intervention assessments. All participants will be randomly assigned to the Mio-Training or the waiting control group, stratified by age and sex.

**Results:**

This study protocol describes the study design of the randomized controlled trial evaluating the efficacy of the Mio-Training. The project is funded from October 2024 to December 2027. Recruitment for healthy controls has been completed (n=40; October 2024-August 2025), recruitment for childhood cancer survivors (n=10, 25% participants recruited) is scheduled from August 2025 to December 2027, and recruitment for participants with ADHD (n=39, 97.5% recruited) is scheduled from October 2025 to September 2026. Data analyses have not yet commenced; first results from the ADHD subgroup are expected in early 2027, with findings from the cancer survivor subgroup anticipated in early 2028 following completion of recruitment.

**Conclusions:**

To strengthen cognitive development in young patients with atypical development, it is necessary to address the current lack of effective treatment options. The combination of cognitive and motor training with metacognitive abilities may support patients’ cognitive maturation trajectories and will enable transfer of the training effect to everyday and school situations.

## Introduction

Many diseases during childhood and adolescence such as cancer or attention-deficit/hyperactivity disorder (ADHD) have an impact on the development of the brain and place children and adolescents at increased risk for cognitive long-term impairments [1-4]. These cognitive impairments can lead to difficulties in everyday and school life, decreased quality of life, and may interfere with the achievement of developmental milestones [5-8]. Through pruning and myelination, there is a rapid increase in white matter and a decrease in gray matter in the child’s brain [9]. Childhood is a maturation period that comes with high cerebral plasticity, and there is evidence that cognitive training during childhood and adolescence entails rapid neural changes [10]. On the basis of the abovementioned knowledge, we developed a cognitive training that supports this sensitive phase of brain development in healthy children and adolescents as well as in patients with cancer or ADHD.

Most cognitive trainings currently available for children and adolescents have only limited efficacy and show limited transfer to nontrained tasks, particularly everyday functioning [11-14]. Intensive training programs often focus on a specific cognitive function (ie, working memory), and different meta-analyses show that intensive training of isolated cognitive functions improves the trained function immediately after the training but shows limited transfer to nontrained tasks, and long-term effects are lacking [11,13].

There is a close relationship between cognitive functions and motor abilities (eg, motor coordination) due to shared cognitive and neural mechanisms between cognitive processes and motor abilities [15]. Studies with pediatric cancer survivors, children and adolescents with ADHD, and healthy children and adolescents show that physical activities can have a positive effect on cognition and even on quality of life [16-19]. However, recent empirical evidence indicates that the type of physical exercise matters to obtain the largest benefits for cognitive functions [20-22]. Pure endurance exercise is thought to be less suitable for promoting cognitive development [23-25]. Instead, physical exercises that include both cognitive and physical demands are more likely to touch upon and thus improve cognition, such as executive functions [21,26-29]. Meta-analyses show that physical activities with cognitive engagement or thoughtful reflections (ie, metacognitive abilities) support the development of executive functions (ie, the ability to inhibit interference, routine thoughts, and impulsive behaviors) [21,26].

Besides intensive training of isolated functions and motor training, the acquisition of mnemonic strategies can enhance cognitive functions. Teaching mnemonic strategies not only leads to improvements in memory functions but also in untrained cognitive domains such as reading and attention in the short and long term [30-33]. Mnemonic strategies are often used in adult neurorehabilitation to enhance learning and recall after brain injury [34]. During and after the acquisition of mnemonic strategies, individuals are thought to develop metacognitive abilities, namely, the ability to supervise, manage, and monitor cognitive processes and to use this knowledge to regulate behavior, cognition, and motor abilities [35,36].

Metacognition refers to the awareness and knowledge an individual has of their own cognitive processes (also referred to as “thinking about thinking”). Metacognitive abilities are a cross-functional set of skills that has a positive effect on school success, social behavior, and a variety of everyday situations [35,37]. An improvement of metacognitive abilities comes along with increased awareness and knowledge of one’s own cognitive and motor performance. Metacognition is the knowledge that allows individuals to use cognitive and motor resources to best plan, monitor, and evaluate behavior, cognition, and motor abilities. Interventions focusing on metacognitive abilities in children showed positive effects on metacognitive abilities, self-efficacy, executive functions, and academic skills [38]. Various models exist to describe applications of metacognitive abilities, all of them sharing the importance of engaging in a variety of processes to plan, monitor, and evaluate performance [39-41].

A recent review demonstrates the positive effects of serious games as an intervention for pediatric patients with cancer [42] because they may help in a playful manner to increase compliance by being intrinsically motivating, highly engaging, and easily accessible at any time, regardless of age and gender [43]. In patients with ADHD, digital health interventions presented via a serious game showed positive effects on cognitive outcomes [44]. A meta-analysis comparing serious games versus conventional instruction methods (ie, lectures, reading, drill, and practice) revealed stronger effects of serious games on learning and retention than conventional instruction methods [45]. Interventions that do not require additional visits to a facility reduce participants’ burden, yield higher compliance rates, and are highly attractive to children and adolescents with cancer and ADHD and their families due to the autonomous handling [46].

On the basis of the background above, we developed the Mio-Training, an easily accessible, playful, multicomponent digital intervention that stimulates metacognitive abilities through the combination of motor coordination and cognitive training in a serious game. We evaluate the efficacy of the Mio-Training in a randomized controlled trial (RCT) in 3 groups of children and adolescents, namely children and adolescents with cancer, children and adolescents with ADHD, and healthy controls. We include healthy children and adolescents to evaluate the feasibility of the Mio-Training and to compare their data with those of the children and adolescents with cancer and ADHD.

The primary objective of this study is to investigate the short- and long-term efficacy of the Mio-Training on metacognitive abilities assessed with the Junior Metacognitive Awareness Inventory (Jr.MAI; primary outcome) [47]. Furthermore, we will investigate the efficacy of the Mio-Training on executive functions, processing speed, and memory (secondary outcomes). Additionally, the feasibility of the Mio-Training will be assessed (ie, usability, enjoyment, level of autonomy, and perceived impact).

We hypothesize that 5 weeks of Mio-Training will lead to improved metacognitive abilities in the short and long term (primary outcome) in children and adolescents with cancer and ADHD. Furthermore, an increase in metacognitive abilities will be associated with an increase in objective performance in executive functions, processing speed, and memory in the short term and with an increase in subjective performance in executive functions in the long term (secondary outcomes). In addition, we hypothesize that the Mio-Training will be perceived as feasible by the healthy children and adolescents and the patients with cancer and ADHD.

## Methods

### Design and Setting of the Study

The study is an investigator-initiated RCT including 2 experimental groups (Mio-Training and a waiting control group). We will investigate the efficacy of the Mio-Training using the same study design in three subgroups, namely (1) healthy children and adolescents, (2) children and adolescents with cancer, and (3) children and adolescents with ADHD.

**Figure 1.**
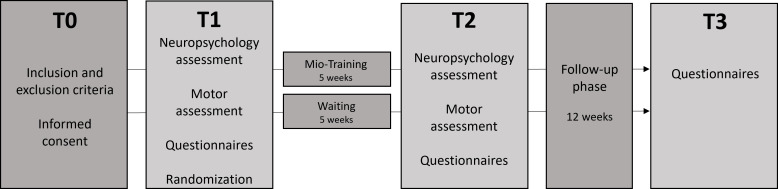
Study flow chart. T0: screening; T1: first assessment (before intervention or waiting period); T2: second assessment (immediately after intervention or waiting period); T3: third assessment (12 weeks after intervention or waiting period).

Participants will be screened for eligibility according to the inclusion and exclusion criteria by a study investigator (for details, see participants section [cancer survivors, children and adolescents with ADHD and healthy controls]). After the screening, 3 study visits will take place at the Division of Neuropediatrics, Development and Rehabilitation at the Inselspital in Bern or at the participants’ home. Cognitive and motor assessments will be performed before the intervention and the waiting period (baseline assessment; T1) and will be repeated after 5 weeks at immediate follow-up (T2). The questionnaires will be completed again at a 3-month follow-up (T3; Figure 1).

### Participants

#### Overview

In total, 120 German- or French-speaking children and adolescents aged 8 to 16 years will be included in the study. In each subgroup (cancer, ADHD, and healthy controls), 40 (33.3%) participants will be randomly allocated to either the Mio-Training (n=20) or the waiting control group (n=20), with age (8 to <12 years vs 12 to 16 years) and sex (male vs female) as stratification factors (1:1 allocation ratio). The randomization sequence will be generated by an independent statistician and implemented in REDCap (Research Electronic Data Capture; Vanderbilt University) to ensure concealment of allocation. Randomization will be performed at the study site (Children’s University Hospital Bern).

#### Cancer Survivors

Forty children and adolescents with a previous diagnosis of cancer with or without central nervous system involvement who are between 3 months before and 10 years after termination of treatment (chemotherapy, and/or radiotherapy, and/or surgery) will be included. The following exclusion criteria will be applied: (1) unstable neurological condition (eg, epilepsy), (2) a severe psychiatric disease (eg, eating disorder) or severe learning disability, (3) known or suspected noncompliance, (4) inability to follow the procedures of the study, (5) IQ <85, or (6) a history of cancer without central nervous system involvement and only surgical removal of the tumor without subsequent radiation and/or chemotherapy. Cancer survivors will be recruited at the Division of Neuropediatrics, Development and Rehabilitation at the Inselspital in Bern and other children’s hospitals in Switzerland through flyers.

#### Children and Adolescents With ADHD

Forty children and adolescents with ADHD will be included in the study. All types of ADHD will be included (inattentive, hyperactive, and combined type). The following exclusion criteria will be applied: (1) unstable neurological condition (eg, epilepsy), (2) severe psychiatric disease (eg, eating disorder) or severe learning disability influencing the development, (3) known or suspected noncompliance, (4) inability to follow the procedures of the study, or (5) IQ <85. Patients with ADHD will be recruited at the Division of Neuropediatrics, Development and Rehabilitation at the Inselspital in Bern and at other institutions in Switzerland through flyers.

#### Healthy Controls

Forty healthy children and adolescents will be included. The following exclusion criteria will be applied: (1) known neurological, (2) psychiatric, (3) other chronic disease influencing the cognitive development, or (4) IQ <85.

### Ethical Considerations

The study with the healthy children and adolescents was approved by the ethics committee of the University of Bern (2023-12-05). The study with the children and adolescents with cancer (2023‐01196) and the study with the children and adolescents with ADHD (2023‐01189) were approved by the cantonal ethics committee of Bern. The study will be conducted in accordance with the current version of the World Medical Association Declaration of Helsinki [48], the International Council for Harmonisation Good Clinical Practice guidelines, and the local legally applicable requirements. All participants and/or one parent or legal guardian will sign a written informed consent.

The studies were registered at ClinicalTrials.gov (cancer and healthy controls: NCT06464237; ADHD: NCT07162831).

### Intervention

**Figure 2.**
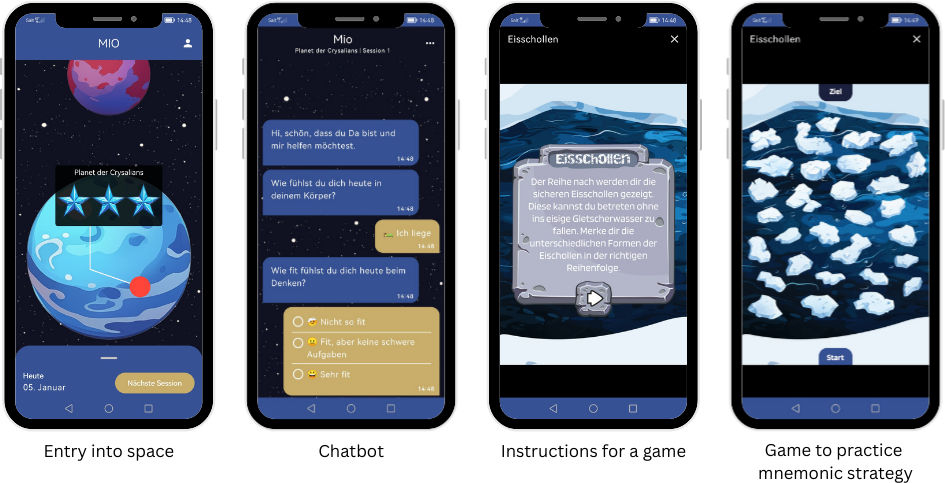
Prototype of the Mio-Training app.

The intervention consists of the Mio-Training (Figure 2), a training app developed by a multidisciplinary team of experts at the Division of Neuropediatrics, Development and Rehabilitation at the Inselspital in Bern in collaboration with the software development company Pioneo GmbH. The Mio-Training stimulates metacognitive abilities in a playful manner using a serious game, a journey through space. The Mio-Training is a multicomponent training program, and each session includes intensive visual and verbal working memory training, the teaching and practicing of 5 mnemonic strategies and memory tasks, a motor coordination exercise, and metacognitive questions that stimulate reflection on cognitive processes through a chatbot. The content of the Mio-Training is based on the knowledge and experience from previous cognitive training and motor intervention studies [23,25,32,33,49-51]. Children and adolescents are encouraged to train 3 times per week for 15 to 20 minutes per training session, resulting in 15 training sessions in total (approximately 300 min of training).

To maximize adherence to the Mio-Training, a user-friendly design that includes reward elements and elements of gamification in the framework of a chatbot was incorporated. To monitor adherence to the Mio-Training, minimal usage data, including length, frequency, compliance, retention rates, chosen difficulty levels, and within-training performance levels will be recorded during the use of this training. These data will be used to monitor compliance. A study investigator will have insight into the usage data on a regular basis, and the participant will be contacted if noncompliance is observed.

### Outcome Measures

Baseline characteristics (age, sex, and socioeconomic factors) and clinical characteristics will be assessed through clinical chart review and questionnaires.

#### Primary Outcome

The primary end point will be the change score in metacognitive abilities, as measured by the Jr.MAI [47] calculated based on the change between baseline and the 3-month follow-up assessment. The score summarizes 18 items and evaluates metacognitive abilities, focusing on metacognitive knowledge and regulation. The score ranges between 18 and 90, with higher values indicating better metacognitive abilities. Research findings confirmed the validity of the Jr.MAI and demonstrated the reliability of the overall score with a Cronbach α of 0.87 [52]. The Jr.MAI will be assessed in all subgroups and both experimental groups (Mio-Training vs waiting control) before and after the training and waiting period and at 3-month follow-up.

#### Secondary and Additional Outcomes

The secondary end points evaluate changes in cognitive functions between T1, T2, and T3 with different standardized tests.

The neuropsychological assessment will be performed at T1 and T2 (Figure 1 and Table 1) by trained psychology students and neuropsychologists. All children and adolescents will undergo an assessment of general intelligence (short form of the Wechsler Intelligence Scale for Children–Fifth Edition [WISC-V]) [53], which will be performed only at T1. The following executive functions will be assessed: working memory (Working Memory Index; WISC-V) [53], cognitive flexibility and inhibition (Color-Word Interference Test; Delis-Kaplan Executive Function System [D-KEFS]) [54], and planning (Tower of Hanoi; D-KEFS) [54]. Furthermore, processing speed (Processing Speed Index; WISC-V) [53], verbal short- and long-term memory (Verbal Learning and Memory Test; VLMT) [55] and visuospatial short- and long-term memory (Battery for Assessment in Children–Merk- und Lernfähigkeitstest; Basic-MLT) [56] will be assessed. In the ADHD group, selective attention and divided attention (Go/Nogo, divided attention; Test of Attentional Performance; TAP) [57] will also be assessed.

**Table 1.** Detailed information about neuropsychological and motor assessment and questionnaires.

Assessment	Instrument	Time points	Populations	Reference
Neuropsychological assessment				
IQ	Short version WISC-V^a^	T1^b^	Healthy, cancer, and ADHD^c^	[53]
Executive functions				
Working memory	Working memory index (WISC-V)	T1 and T2^d^	Healthy, cancer, and ADHD	[53]
Inhibition	Color-Word Interference Test (D-KEFS^e^)	T1 and T2	Healthy, cancer, and ADHD	[54]
Cognitive flexibility	Color-Word Interference Test (D-KEFS)	T1 and T2	Healthy, cancer, and ADHD	[54]
Planning	Tower of Hanoi (D-KEFS)	T1 and T2	Healthy, cancer, and ADHD	[54]
Processing speed	Processing speed index (WISC-V)	T1 and T2	Healthy, cancer, and ADHD	[53]
Attention				
Selective attention	Go/Nogo (TAP^f^)	T1 and T2	ADHD	[57]
Divided attention	Divided attention (TAP)	T1 and T2	ADHD	[57]
Memory				
Verbal short- and long-term memory	VLMT^g^	T1 and T2	Healthy, cancer, and ADHD	[55]
Visual short- and long-term memory	Pattern learning (Basic-MLT)^h^	T1 and T2	Healthy, cancer, and ADHD	[56]
Motor assessment				[58]
Balancing	DMT^i^	T1 and T2	Healthy, cancer, and ADHD	
Jumping back and forth sideways	DMT	T1 and T2	Healthy, cancer, and ADHD	
Torso bend	DMT	T1 and T2	Healthy	
Push-ups	DMT	T1 and T2	Healthy	
Sit-ups	DMT	T1 and T2	Healthy	
Standing long jump	DMT	T1 and T2	Healthy	
Questionnaires				
Metacognitive abilities	Jr.MAI^j^	T1, T2, and T3^k^	Healthy, cancer, and ADHD^l^	[47,52]
Executive functions	BRIEF^m^	T1, T2, and T3	Healthy, cancer, and ADHD^n^	[59]
ADHD symptoms	Conners 3^o^	T1, T2, and T3	ADHD^p^	[60]
Resources	FRKJ^q^	T1, T2, and T3	Healthy, cancer, and ADHD^l^	[61]
Fatigue	PedsQL^r^	T1, T2, and T3	Healthy and cancer^n^	[62]
Physical self-concept	PSDQ-S^s^	T1, T2, and T3	Healthy, cancer, and ADHD^l^	[63]

a WISC-V: Wechsler Intelligence Scale for Children–Fifth Edition.

b T1: first assessment (before intervention or waiting period).

c ADHD: attention-deficit/hyperactivity disorder.

d T2: second assessment (immediately after intervention or waiting period).

e D-KEFS: Delis-Kaplan Executive Function System.

f TAP: Test of Attentional Performance.

g VLMT: Verbal Learning and Memory Test.

h Basic-MLT: Battery for Assessment in Children–Merk- und Lernfähigkeitstest.

i DMT: German Motor Test.

j Jr.MAI: Junior Metacognitive Awareness Inventory.

k T3: Third Assessment (12 weeks after intervention or waiting period).

l Self-rating.

m BRIEF: Behavior Rating Inventory of Executive Function.

n Parent and Self-rating.

o Conners 3: Conners, Third Edition.

p Parent Rating.

q FRKJ: Questionnaire on Resources in Childhood and Adolescence.

r PedsQL: Multidimensional Fatigue Scale.

s PSDQ-S: physical self-description questionnaire–short version.

The motor assessment will be performed at T1 and T2 (Figure 1 and Table 1). The following subtests of the German motor performance test will be performed: balancing, jumping back and forth sideways, torso bend, push-ups, sit-ups, and standing long jump [58].

The participants and parents will fill out questionnaires using a REDCap survey at T1, T2, and T3 (German and French versions; Figure 1 and Table 1). Questionnaires will be used to assess metacognitive abilities (Jr.MAI) [47], executive functions and metacognition (Behavior Rating Inventory of Executive Function) [59], personal and social resources (Questionnaire on resources in childhood and adolescence) [61], fatigue (Multidimensional Fatigue Scale) [62], physical self-concept (physical self-description questionnaire–short version) [63] and feasibility of the Mio-Training (self-made questionnaire). Additionally, for children with ADHD, the intensity of ADHD symptoms will be assessed (Conners, Third Edition) [60] at T1.

### Statistical Analysis

The sample size was calculated for the primary outcome measure (Jr.MAI total score) using G*Power 3: repeated measures ANOVA (within-between subjects interaction); small effect size (Cohen *f*^2^=0.25); α=.05, power=0.8; retest correlation=0.5. The resulting minimal sample size is 14 participants per experimental group (Mio-Training vs waiting control group), corresponding to n=28 per subgroup (healthy controls, children and adolescents with cancer, and children and adolescents with ADHD). To compensate for losses and dropouts, the sample size was defined as 40 participants per subgroup, with a total sample size across all 3 subgroups of 120.

Statistical analyses will be conducted using RStudio (Posit PBC). The planned statistical analyses were developed in consultation with an experienced statistician. The level of significance is set at α=.05. Data will be tested for normal distribution. The primary analysis will follow an intention-to-treat approach using linear mixed-effects models to calculate 2-sided 95% CIs for the primary outcome (Jr.MAI total score). The mixed-effects model will contain the total score of the Jr.MAI, the time points (ie, baseline, 5-week follow-up, or 3-month follow-up, as a categorical variable), treatment allocation (ie, Mio-Training vs waiting control group), the baseline score of the Jr.MAI (to adjust for individual differences in baseline functioning and to improve statistical precision), and stratification factors as fixed effects. The primary effect of interest is the treatment allocation-by-time point interaction. Participant ID will be included as a random intercept. Secondary end points will be analyzed using the same method. A per-protocol analysis will be conducted as a sensitivity analysis using the same model structure restricted to adherent participants (≥80% intervention compliance). Additional sensitivity analyses will examine differences in slopes over time between groups. Subgroup analyses will investigate potential effect modification by sex and age via interaction terms (treatment×sex and treatment×age), with stratified results reported where appropriate. Missing data exceeding 5% will be handled using multiple imputation by chained equations (50 datasets), incorporating all baseline and outcome variables at all time points as predictors. Rubin’s rules [64] will be used for pooling estimates. Variables with more than 50% missing values will not be used for the imputation model. To address multiplicity arising from secondary and subgroup analyses, a false discovery rate correction will be applied where appropriate. The primary analysis will not be adjusted for multiplicity, as it is prespecified and confirmatory. Secondary and exploratory subgroup analyses (including sex and age interactions) will be interpreted with false discovery rate–adjusted *P* values to control for type I error inflation arising from multiple comparisons.

### Safety and Monitoring

Although the intervention is considered low risk, safety monitoring procedures were implemented for all subgroups. Potential adverse events include tiredness, frustration, distress, muscle soreness, or overexertion during or after the training sessions. Adverse events are monitored throughout the study via participant self-report, caregiver feedback, and study team observations during assessments. All reported events are documented and reviewed by the study team on an ongoing basis. In case of any adverse reactions, training sessions are paused or discontinued as appropriate, and participants are referred for clinical evaluation if necessary.

### Data Management

The case report forms will be stored in a dedicated electronic data capture system (REDCap [65]) hosted by the Clinical Trials Unit Bern of the Faculty of Medicine of the University of Bern and the Inselspital, Bern University Hospital, Switzerland.

### Data Monitoring

On-site monitoring will be part of the quality control activities implemented. Data will be monitored on a regular basis, including a quality check of the data performed by the principal investigator or his designees.

## Results

The project is funded from October 2024 to December 2027. Data collection for the study with the healthy controls started in October 2024 and ended in August 2025. Data collection for the study with the cancer survivors started in August 2025 and is expected to continue until the end of 2027, while the study with the children with ADHD began in October 2025 and is projected to end in September 2026.

Forty participants have been recruited and assessed in the healthy control group, 10 (25%) participants in the cancer survivor group, and 39 (97.5%) participants in the ADHD group. In total, 40 participants per subgroup will be recruited. Data analyses have not yet commenced. First results from the ADHD study are expected to be published in late 2026 or early 2027. The healthy control data will not be published as a separate study, except for the feasibility results, but will be analyzed in comparison with the ADHD and cancer survivor groups. As recruitment for the cancer survivor study is more challenging and is expected to continue until the end of 2027, the ADHD study will be analyzed and published first. Publications from the cancer survivor study are anticipated for early 2028.

## Discussion

The Mio-Training is a newly developed multicomponent digital intervention designed to address a critical gap in the field, namely, the lack of multimodal, transferable, and accessible training approaches for children and adolescents with cancer and ADHD that focus on everyday functioning rather than isolated cognitive functions. By stimulating metacognitive abilities through the combination of intensive working memory training, mnemonic strategies, and motor coordination within a serious game, the Mio-Training aims to strengthen cognitive development in children and adolescents with atypical brain development. We anticipate that 5 weeks of Mio-Training will lead to improvements in metacognitive abilities, with associated gains in executive functions, processing speed, and memory that transfer beyond trained tasks and persist over time. To evaluate its feasibility and efficacy, we conduct a RCT including 3 pediatric subgroups (ADHD, cancer, and controls).

The development of the Mio-Training is grounded in evidence suggesting that isolated cognitive or motor training often yields only modest and domain-specific improvements, with limited transfer to everyday and school functioning [11]. Meta-analyses and longitudinal studies have shown that while cognitive training programs—particularly intensive working memory or attention training—can produce short-term gains, these effects lack generalization and sustainability in the long term [66]. Similarly, motor-based interventions, though beneficial for motor coordination and certain aspects of executive functioning, rarely lead to long-term improvements when implemented in isolation [67]. Hence, the Mio-Training stimulates metacognitive abilities during the acquisition of mnemonic strategies, the performance of intensive working memory training, and motor coordination tasks. The multimodal approach presented in the Mio-Training emphasizes the interactive and complementary nature of cognitive development and addresses the limitations of unimodal interventions.

Integrating metacognitive abilities into cognitive training is a critical factor in enhancing training efficacy, particularly in pediatric populations where introspection, self-reflection, and thinking about cognitive self-optimization are still absent or only emerging [35-41,68]. Metacognitive abilities—such as reflecting on one’s own thinking, learning and mnemonic strategies, and task performance—promote self-regulated learning and deeper cognitive engagement. Research has shown that metacognitive abilities (ie, planning, monitoring, and evaluating one’s own cognitive processes) are strongly associated with academic achievement, problem-solving abilities, and long-term school outcomes [69]. In children, especially those with atypical development, metacognitive abilities can encourage active participation in their learning, which can enhance motivation, persistence, and self-efficacy and, through this, support the transfer of trained skills (ie, the application of mnemonic strategies) to school and everyday life [35-41,68]. Thus, metacognitive abilities do not merely supplement cognitive training but rather enhance its relevance and long-term efficacy. Strong metacognitive abilities can give individuals a sense of agency over their rehabilitation journey [40].

Increasing evidence from developmental neuroscience suggests that motor coordination and cognitive functions, particularly executive functions and working memory, share overlapping neural networks, including the cerebellum, prefrontal cortex, and parietal regions [70,71]. Coordinative motor activities require goal-directed behavior, inhibition, and attentional control—all core components of executive functioning. Incorporating motor coordination exercises into cognitive training can therefore support the development of higher-order cognitive skills. Coordination tasks can thus enhance cognitive processes through embodied learning mechanisms [72]. Moreover, coordination tasks are often of a playful nature and hence increase motivation, engagement, and the experience of self-efficacy. The multimodal stimulation may be particularly beneficial for children and adolescents with atypical development [73], such as those with ADHD or pediatric cancer, whose neural systems may require multisensory and integrative stimulation to strengthen cognitive, everyday, and school performance.

To evaluate the efficacy of the Mio-Training scientifically, the implementation of an RCT is essential before its possible application in clinical practice. As the gold standard in intervention research, RCTs minimize bias, control for confounding variables through random allocation, and enable the detection of intervention effects by comparing outcomes between the intervention and control groups. Moreover, evaluating the Mio-Training not only in clinical populations (namely children with ADHD or pediatric cancer) but also in a healthy control sample allows for the assessment of differential responsiveness to the training across groups. This will allow insight into whether the training has specific benefits for populations with atypical development or whether its effects generalize to typically developing children.

The Mio-Training is presented in a well-designed, visually appealing, and intuitively navigable serious game—a journey through space—with a rewarding and immersive environment that can ease the use of the training program. All of these factors are particularly critical in pediatric populations to balance therapeutic attempts with user-centered design to enhance motivation, repeated use, engagement, and adherence and, through this, hopefully the acceptability and efficacy of the training.

In light of increasing economic constraints and staff shortages in educational and clinical settings, there is an urgent need for cognitive interventions that are both effective and easy to implement in environments where resources are limited. The low-threshold, user-friendly Mio-Training offers a promising tool to address both the needs of children and adolescents with atypical development and these systemic challenges.

Presenting the efficacy of a multimodal training program—the Mio-Training—is of high relevance to both clinical and scientific staff. For clinicians, the Mio-Training offers a training protocol that strengthens cognitive development in patients without much staff support; for researchers, it provides a framework to investigate different driving modes and their effects on neuroplasticity in vulnerable developmental populations.

Several limitations warrant consideration. As a protocol paper, all findings are anticipated rather than empirically established. The sample sizes within each subgroup are relatively small, which may limit statistical power and the generalizability of findings. Both clinical subgroups (children with cancer and children with ADHD) are inherently heterogeneous with respect to diagnosis, treatment history, and disease severity, which may introduce considerable variability in training response and complicate between-group comparisons. Blinding of participants is not possible given the nature of the intervention, and differential effects based on prior gaming experience or digital access cannot be excluded.

Future studies could use factorial trial designs to disentangle the relative contributions of the individual training components (motor coordination, working memory training, mnemonic strategies, and metacognitive strategy acquisition) to better understand which elements drive observed effects and for whom. Longer follow-up periods and neuroimaging studies could further clarify the durability and neural correlates of training-induced changes.

Findings will be published in open-access peer-reviewed journals and presented at relevant conferences in pediatric oncology and neuropsychology. Results will also be communicated to patient advocacy groups and clinical networks to support translation into practice.

## Supplementary material

10.2196/95139 Checklist 1 SPIRIT 2025 checklist.

10.2196/95139 Peer Review Report 1 Peer Review Report by the Swiss Cancer Research Foundation.

## Abbreviations

ADHD: attention-deficit/hyperactivity disorder
D-KEFS: Delis-Kaplan Executive Function System
Jr.MAI: Junior Metacognitive Awareness Inventory
RCT: randomized controlled trial
REDCap: Research Electronic Data Capture
WISC-V: Wechsler Intelligence Scale for Children–Fifth Edition
