# Supplementary material for: A Metacognitive Intervention for Children and Adolescents in Neuropediatric Care (Mio-Training): Protocol for a Randomized Controlled Trial
Source: JMIR Res Protoc. 2026 Jun 26;15:e95139. doi: 10.2196/95139 (PMC13309103; doi:10.2196/95139)
Supplement: Peer Review Report 1 [file resprot-v15-e95139-s002.pdf]

krebsforschung schweiz
recherche suisse contre le cancer
ricerca svizzera contro il cancro
swiss cancer research

krebsliga schweiz
ligue suisse contre le cancer
lega svizzera contro il cancro

Application Summary

Principal Applicant	Prof. Dr. Regula Everts
Title	Training the body and the brain of children with cancer using an E-health tool
Reference	KFS-5515-02-2022
Total Requested	CHF 315,021.00

1. Project summary

Is this a new project, continuation or revised version?	Revised

Original project number
Grant selected has been removed.

Original project number (if not shown above)
KFS-5260-02-2021

Start date	01.08.2022

Proposed duration of funding (in months)	36

Keyword classifications
Please provide a minimum of 5 and a maximum of 8 keywords.

Children
physical activity
interdisciplinary approach
neuropsychology
prevention

Propose new keyword classifications<br>You may optionally propose new keywords to be added to the list by recording them here.
sport science

Domain classifications
Select the most appropriate domain classification for this application (select only one). This helps allocate your application during the review process. We may reallocate your application to another area if we consider it appropriate.

Domains

Clinical

patient oriented research

2. Principal applicant

**Title**	Prof. Dr.	**Address Line 1**	Freiburgstrasse
**Forename(s)**	Regula	**Address Line 2**	
**Surname**	Everts	**Address Line 3**	
**Nationality**	Swiss	**City/Town**	Bern
**Date of Birth**	12.08.1975	**Country**	Switzerland
**Institution**	Inselspital Bern	**Postcode**	3010
**Department**	Children´s University Hospital	**Telephone No.**	+41 31 632 41 30
		**Email Address**	regula.everts@insel.ch

**ORCID iD**<br>If you have an ORCID iD, please paste it in the field provided.
0000-0001-6556-3419

Principal Applicant CV & Publications List Upload

(Applicant CV - Everts_CV.pdf) is included as an appendix within this file.
(Applicant Publications List - Everts_Publications.pdf) is included as an appendix within this file.

3. Co-Applicants

**Title**	Dr.	**Address Line 1**	Bremgartenstrasse 215
**Forename(s)**	Valentin	**Address Line 2**	
**Surname**	Benzing	**Address Line 3**	
**Nationality**	German	**City/Town**	Bern
**Institution**	University of Bern	**Country**	Switzerland
**Department**	Institute of Sport Science	**Postcode**	3027
		**Telephone No.**	+41 76 622 00 12
		**Email Address**	valentin.benzing@ispw.unibe.ch

**Title**	Dr. med.	**Address Line 1**	Freiburgstrasse
**Forename(s)**	Sebastian	**Address Line 2**	
**Surname**	Grunt	**Address Line 3**	
**Nationality**	Swiss	**City/Town**	Bern
**Institution**	Inselspital Bern	**Country**	Switzerland
**Department**	Pediatrics	**Postcode**	3010
		**Telephone No.**	+41 31 632 31 10
		**Email Address**	sebastian.grunt@insel.ch

**Title**	Dr.	**Address Line 1**	Pfingstweidstrasse 96
**Forename(s)**	Anna Lisa	**Address Line 2**	Postfach
**Surname**	Martin-Niedecken	**Address Line 3**	
**Nationality**	German	**City/Town**	Zurich
**Institution**	Zurich University of the Arts	**Country**	Switzerland
**Department**	Department Design	**Postcode**	8031
		**Telephone No.**	+41 78 656 04 45
		**Email Address**	anna.martin@zhdk.ch

**Title**	Prof. Dr.	**Address Line 1**	Freiburgerstr.
**Forename(s)**	Jochen	**Address Line 2**	
**Surname**	Rössler	**Address Line 3**	
**Nationality**	German	**City/Town**	Bern
**Institution**	Inselspital Bern	**Country**	Switzerland
**Department**	Pediatric Hematology and Oncology	**Postcode**	3010
		**Telephone No.**	+41 31 632 94 95
		**Email Address**	jochen.roessler@insel.ch

Co-applicant attachments

CO-APPLICANT ATTACHMENT SET 1

Co-applicant Name
Valentin Benzing

(Co-applicant CV - Benzing_CV.pdf) is included as an appendix within this file.
(Co-applicant Publications List - Benzing_Publications.pdf) is included as an appendix within this file.

CO-APPLICANT ATTACHMENT SET 2

Co-applicant Name
Sebastian Grunt

(Co-applicant CV - Grunt_CV.pdf) is included as an appendix within this file.
(Co-applicant Publications List - Grunt_Publications.pdf) is included as an appendix within this file.

CO-APPLICANT ATTACHMENT SET 3

Co-applicant Name
Jochen Rössler

(Co-applicant CV - Roessler_CV.pdf) is included as an appendix within this file.
(Co-applicant Publications List - Roessler_Publications.pdf) is included as an appendix within this file.

CO-APPLICANT ATTACHMENT SET 4

Co-applicant Name
Anna Lisa Martin-Niedecken

(Co-applicant CV - Martin-Niedecken_CV.pdf) is included as an appendix within this file.
(Co-applicant Publications List - Martin-Niedecken_Publications.pdf) is included as an appendix within this file.

---

4. External reviewers

Excluded Reviewers
If you wish to exclude certain reviewers from the evaluation process, please provide their exact names, work affiliations, email addresses and brief reason for exclusion below.

**Title**	Prof.
**First Name**	Claudia
**Last Name**	Roebers
**Expertise**	-
**Department**	Institute of Psychology
**Institution**	University of Bern
**Email**	claudia.roebers@psy.unibe.ch
**Reason**	Prof. Roebers investigates metacognition in experimental research designs and repeatedly strongly critizied the methods of our clinical research.

Recommended Reviewers
Please provide us with names, work affiliations and email addresses of 2-5 external reviewers below.

**Title**	Prof.
**First Name**	Peter
**Last Name**	Anders
**Expertise**	He is an expert in studying the impact brain injury and brain development has on cognitive, motor, educational and behavioural outcomes in high-risk children. Part of his research focuses on the efficacy of cognitive interventions during childhood.
**Department**	Monash Institute of Cognitive and Clinical Neurosciences, Monash University
**Institution**	Murdoch Children's Research Institute
**Email**	peter.anderson@mcri.edu.au

**Title**	Prof.
**First Name**	Vicki
**Last Name**	Anderson
**Expertise**	She is an expert on the investigation of developmental alterations in early childhood and how to improve cognitive and motor development in children at risk
**Department**	Royal Children's Hospital, Melbourne, Australia

**Institution**	Murdoch Children's Research Institute
**Email**	vicki.anderson@mcri.edu.au

**Title**	Prof.
**First Name**	Stefan
**Last Name**	Göbel
**Expertise**	He is an expert on the development of serious games in the domain of health sciences
**Department**	Multimedia comminucations lab
**Institution**	Technical University Darmstadt, Germany
**Email**	stefan.goebel@kom.tu-darmstadt.de

**Title**	Prof.
**First Name**	Tom
**Last Name**	Baranowski
**Expertise**	He is an expert in creation of computer adapted tests promoting physical activity, exergames in children
**Department**	Children's Nutrition Research Center
**Institution**	Baylor College of Medicine, Houston, USA
**Email**	tbaranow@bcm.edu

**Title**	Dr.
**First Name**	Fiona
**Last Name**	Streckmann
**Expertise**	She is an expert in physical and movement science and investigates around the effect of physical acticity in children with cancer
**Department**	Departement für Sport, Bewegung und Gesundheit
**Institution**	Universität Basel Schweiz
**Email**	fiona.streckmann@unibas.ch

5. Research summary

Research summary / Abstract<br>Provide a summary of your proposed research, including key goals. This information is for a scientific expert audience.
To prevent and counteract cognitive and physical difficulties in children with cancer, effective and appealing interventions are needed. Currently, only few evidence-based interventions tailored to the needs of children with cancer are available. Our previous research is in accordance with other literature that gives valuable insights into what would be necessary and what can be neglected in future interventions for children with cancer (Siegwart et al., 2020; Benzing et al., 2020; Benzing et al., 2021; AbdMajid et al., 2020). In summary, computerized cognitive trainings often yield only limited improvements in cognition and a lack of transfer effects to non-trained cognitive domains, despite the manifold use of such programs in everyday clinical care. However, a transfer effect can be accomplished through cognitive strategy training, such as the teaching of mnemonic strategies (Everts et al., 2017). During and after the acquisition of mnemonic strategies, individuals develop metacognitive skills ("thinking about one's own thinking") that transfer to many tasks and can be incorporated into a variety of everyday situations. In addition to cognitive training, physical exercise and motor training is highly recommended for children with cancer during clinical care (Benzing et al., 2020). The literature shows that if the physical tasks include cognitive engagement, there is a supplementary positive effect on cognition.<br>Based on the scientific literature and our own research, we will develop and evaluate an e-health application at the exercise-cognition intersection that stimulates physical abilities and executive functions in a playful manner using a serious game. Interventions, which do not require additional visits to a facility reduce the participant's burden, yield higher compliance rates and are highly attractive to children with cancer and their families. The training will incorporate aspects known to be crucial for the efficacy of training programs, including an adaptive difficulty level, motivating feedback, a rich variety of training tasks that transfer into everyday life, fostering self-efficacy and self-confidence, enabling social exchange and including booster sessions that support long-term efficacy.<br>We will evaluate the efficacy of the training App in a randomized controlled trial including 40 children with cancer from the University Hospital Bern, Inselspital. We hypothesize that our 10-12 weeks lasting training program will lead to improved metacognitive abilities (primary outcome) and improved executive functions, motor ability, and physical self-concept (secondary outcomes) in the training group compared to the control group. Further, the positive effect of the training program on the primary and secondary outcomes will be measurable shortly after termination of the training (short-term effect). The improvement in metacognitive abilities (primary outcome) will remain at three months follow-up.<br>Metacognitive skills have been neglected so far in the research on intervention methods that support cognitive and physical development of children with cancer. Our new, combined training approach is therefore closing a gap by incorporating cognitive demands into motor tasks while touching upon metacognitive skills, rather than separating different training approaches as unique entities. We are convinced that the integration of training approaches is the most promising approach to enhance cognitive reserves efficiently in our vulnerable patient group.

Lay Titles & Lay Abstracts

Lay Title (English)<br>Please provide a lay title of your research project in English.
Training the body and the brain of children with cancer using an E-health tool

**Lay Abstract (English)**<br>Please provide a lay abstract (150-250 words) of your research project in English<br>In an effort to help raise public awareness, the lay abstract must be written in a **clear and non-technical language** and explain the content of the research project understandable to the general public.<br>It should include the following aspects: a brief project outline, the project aim(s), the methods and approach and the potential benefit for patients.
**Background**: Children with cancer frequently experience negative long-term effects including cognitive deficits and reduced motor abilities that affect their school performance and quality of life. To prevent and counteract cognitive and physical difficulties of children with cancer, easily accessible, evidence-based, playful interventions are needed. Metacognitive abilities ("thinking about ones` own thinking") have been neglected in training programs so far, despite their central role for cognitive and physical functioning, for school success and quality of life.<br>**Aim**: In this interdisciplinary project, we will evaluate a serious game (game with a purpose) consisting of cognitive and physical tasks that alsostimulate metacognitive thinking. We hypothesize that the training program can improve metacognitive abilities and thereby, foster the cognitive and motor development of children with cancer.<br>**Methods**: In a randomized controlled trial, the serious game will be developed as an App with a thematic framework (a journey around the world) that leads through the intervention. We will evaluate the efficacy of the App in 40 patients with cancer aged 8-12 years. We will assess their metacognitive abilities, cognitive functioning and motor abilities over three time points, namely before and immediately after the training and at three months follow-up.<br>**Benefit for the patients**: This research project supports the development of children with cancer by offering an attractive, gamified e-health application at the exercise-cognition interface. The serious game will have the power to prevent or reduce cognitive and physical problems and improve metacognitive skills. Thus, school achievements and the quality of life of this vulnerable patient group may be improved as well.

**Lay Title (German or French)**<br>Please provide a lay title of your research project in German or French.
Spielbasiertes Training für Kinder mit Krebs an der Schnittstelle zwischen Sport und Kognition

**Lay Abstract (German or French)**<br>Please provide a lay abstract (150-250 words) of your research project in German or French.<br>In an effort to help raise public awareness, the lay abstract must be written in a **clear and non-technical language** and explain the content of the research project understandable to the general public.<br>It should include the following aspects: a brief project outline, the project aim(s), the methods and approach and the potential benefit for patients.
**Hintergrund**: Kinder mit einer Krebserkrankung leiden oft an negativen Langzeitfolgen, wie kognitiven und motorischen Schwierigkeiten, die ihre schulischen Leistungen und ihre Lebensqualität beeinträchtigen. Um kognitive und motorische Probleme bei krebskranken Kinder vorzubeugen und entgegenzuwirken, sind leicht zugängliche, spielbasierte und evidenz-basierte Interventionen an der Schnittstelle zwischen Sport und Kognition notwendig. Metakognitive Fähigkeiten (das Nachdenken über die eigenen Gedanken) sind in bisherigen Trainingsprogrammen vernachlässigt worden und sollten aufgrund ihrer zentralen Rolle für kognitive und körperliche Leistungen bei Kinder mit Krebs gezielt gestärkt werden.<br>**Ziel**: In diesem interdisziplinären Projekt werden wir ein "Serious Game" (zweckgerichtetes Spiel) mit kognitiven und körperlichen Modulen, welche die Metakognition stimulieren, evaluieren. Wir gehen davon aus, dass das Trainingsprogramm die metakognitiven

Fähigkeiten verbessert und dadurch die kognitive und motorische Entwicklung von Kindern mit Krebs nachhaltig begünstigt.
Methoden: In einem randomisierten, kontrollierten Studiendesign wird ein "Serious Game" bei 40 Kindern mit Krebsdiagnose im Alter von 8 bis 12 Jahren auf seine Wirksamkeit überprüft. Über eine App werden metakognitive Fähigkeiten, kognitive Funktionen und motorische Fertigkeiten über drei Zeitpunkte erfasst, nämlich vor und unmittelbar nach dem Training und im 3-monatigen Follow-up.
Benefit: Das vorliegende Forschungsprojekt unterstützt die Entwicklung krebskranker Kinder durch eine einfach verfügbare, attraktive, spielbasierte App. Das Trainingsprogramm wird nicht nur die Kognition und die motorischen Fähigkeiten, sondern auch die Metakognition verbessern und dadurch den Schulerfolg und die Lebensqualität dieser vulnerablen Patientengruppe begünstigen.

**Address of the ethics committee that will be evaluating this application**
Kantonale Ethikkommission Bern (KEK)<br>Hörsaaltrakt Pathologie, Eingang 43A, Büro H372<br>Murtenstrasse 31<br>3010 Bern<br>Tel. +41 31 633 70 70

**Please note the name of the registry where this study is or will be registered**<br>(Mandatory for clinical research applications only)
clinicaltrial.gov and kofam.ch

6. Research plan

Research Plan Information
Please include the following information in one **pdf file** not to exceed a **maximum of 12 pages** (Arial font 10). Be sure to include:

1. a brief description of the current status of research in the field in which the work is planned referring to the most important studies by others
2. a brief description of the status of the applicant's own research in the field in which the work is planned, referring to the most important relevant studies
3. the research plan
4. time-frame for the overall project
5. the significance of the planned work
6. the references.

Questions?
Contact us.
scientific-office@swisscancer.ch
(Research Plan - Research Protocol.pdf) is included as an appendix within this file.
1. Revised Version Research Plan
As you selected **Revised** under **Project summary** of this application form, you will need to attach an updated Research Plan above. We ask that you:

- use **blue and bold font** to distinguish changes and new information in comparison to your initial application. This makes it easier for the reviewers to follow what you have changed.
- Refer to the help text (click on the blue symbol with a question mark) for an example.

(Point to point response - Point to Point Reply(2).pdf) is included as an appendix within this file.

**Futher funding**<br>How is the project under consideration connected to work you are carrying out which is being funded by other bodies (e.g. university, Swiss National Foundation for Scientific Research, cantonal cancer leagues)?
Currently, no other study is carried out at our Department in this thematic domain.

7. Resources

What level of total funding is currently available to you; how much of this total can you use for the project under consideration (not including the funding requested in this application)?

The following details must be supplied:

Personnel
To set up a revised version of the study protocol, we have received the financial support of Prof. Jochen Rösser (Co-applicant) who paied 20% of the salary of a PhD student (Anja Vossenkaul) for 6 months until the submission date (31.01.2022). At the time of the expected project start (August 2022) we have no money available for the development of the present research project.<br><br>The salary of the main applicants' research in the field of pediatric cancer is partly financed by the Division of Neuropediatrics, Development and Rehabilitation (50%) and the Berner Stiftung für krebskranke Kinder (Inselspital, 10%). The main applicant has further research employments at the University Hospital for Diabetology, Endocrinology & Metabolism (10%) and receives a Grant from the Stiftung von Tobel (10%) for another clinical trial (PICO-Study).<br><br>Relating to the research project under consideration, we currently run a study for which a PhD (Kirstin Schürch) analyzes existing data on the effect of physical interventions in children with cancer, funded by the Krebsstiftung Thun-Berner Oberland (co-financing of a PhD position for three years (2020-2023) with 15'000 CHF/year).

Existing equipment
n.a.

Funding required for new equipment, consumables and other expenses
The development of the App will ask for respective devices to run the game-based e-health solution (e.g. mobile phone, PC, TV screen, etc.). We plan to include around 20 patients in the intervention group and assume that we will have to offer either a mobile phone, a computer or a TV screen to these patients (20 x 500 CHF = 10'000 CHF). Furthermore we expect own expenses in regard to the technical equipment of around 2000.-.

Infrastructure (rooms, workshops, computer centres, libraries etc.)
The Divison of Pediatric Oncology/Hematology (Prof. Jochen Roessler), the Division of Neuropediatrics and the Zürcher Hochschule der Künste (Dr. des. Martin-Niedecken) provides the research team with working space, IT-support and access to the library of the University of Bern. The Children´s University<br>Hospital, Inselspital offers its research staff members financial support to travel abroad and present research data at national and international conferences (1500 CHF / year).

**Funding requested from other sources**<br>What level of total funding is currently granted from other sources (amounts, sponsors)?

Unfortunately, no money is available to pay salaries related to the present project.

Applications for financial assistance have also been / will be submitted to the following organisations
We currently plan the submission of the present research protocol to other funding bodies such as the Foundation von Tobel and the Anna Müller Grocholski Foundation. In case of a rejection from the Swiss Cancer Research we will consider the submission to the Swiss National Science Foundation in October 2022 (Project Grant).

8. Funding

What funding will be required during the period of credit?

Name: Kirstin Schürch
Social Security Number: NN
Number of children: 0
Doctoral Candidate?: Yes
Marital status: not married
Nationality: Swiss and Danish
Academic title or diploma: MSc **Since:** 11.8.2021
Type: PhD Student
Function: Study Coordinator and Specialist with cognitive training tasks
% working time spent on project: 100.0
% Social security contributions: 18.0

	Year 1	Year 2	Year 3	Total Cost (CHF)
Basic Salary	55625.00	57395.00	59047.00	172067.00

Name: unknown unknown
Social Security Number:
Number of children:
Doctoral Candidate?: No
Marital status:
Nationality:
Academic title or diploma: Since:
Type: Scientist
Function: Game Developer
% working time spent on project: 60.0
% Social security contributions: 18.0

	Year 1	Year 2	Year 3	Total Cost (CHF)
Basic Salary	65477.00	65477.00	0.00	130954.00

Expenses

Cost Item:	Year 1	Year 2	Year 3	Total Cost (CHF)
Technical Equipment	5000.00	5000.00	2000.00	12000.00

Justification:
**Technical Equipment:** The development of the App will ask for respective devices to run the game-based e-health solution (e.g. mobile phone, PC, TV screen, etc.). We plan to include around 20 children and adolescents with cancer and assume that we will have to offer either a mobile phone, a computer or a TV screen to these patients (5 x 500 CHF = 10'000 CHF).

	Year 1	Year 2	Year 3	Total Cost (CHF)
Expenses Total	5000.00	5000.00	2000.00	12000.00

Totals (CHF)	Year 1	Year 2	Year 3	Overall (CHF)
	126,102.00	127,872.00	61,047.00	315,021.00

9. Declaration

Please confirm by marking the box below:

The institution (organisation, university, hospital, study group etc.) where the project will be carried out, is not active in the field of fund raising for this research work.
☒Confirmed

Please confirm by selecting the appropriate option below:

I hereby declare that I have read the Ethical Principles and Guidelines for Experiments on Animals and will carry out the animal studies approved for my research in accordance with these instructions.
I have read the above and I will comply.

I hereby declare that I have read the Declaration of Helsinki and the Guidelines of Good Clinical Practice (GCP) issued by ICH, Swiss and European regulatory authorities and will carry out the clinical studies approved for my research in accordance with these instructions.
I have read the above and I will comply.

I hereby declare that I will request authorization from the ethics committee or a veterinary office mentioned under Research Summary.
I have read the above and I will comply.

I hereby declare that I will request the clinical trial insurance.	I have read the above and I will comply.

To facilitate communication and increase efficiency within our organization, we would like to transmit information on your project to the cantonal league in your area.<br>Please state whether you agree that the following information about your project (even for projects not approved by the Scientific Committee) can be transmitted: main and co-applicants, title, domain and lay abstracts.
Information about this project can be transmitted to cantonal leagues.

In order to increase the financial resources for funding, we would like to be able to present positively evaluated projects to other foundations.<br>Please state whether you agree that the following information on your project can be transmitted to foundations: main and co-applicants, title, domain and lay abstract.
Information about this project can be transmitted to foundations.

10. Attachments

Supportive documents

Add manuscripts, estimates, support letters and/or other documents which you consider to be supportive of this application.

For clinical trials, please **attach** the clinical study protocol.

Files attached in this section **will not appear** in the application summary pdf (a system generated document used for application submission and review) but **will be accessible online to the assigned panel members and reviewers** during the review process.

11. Appendices

1) Applicant CV - Everts_CV.pdf
2) Applicant Publications List - Everts_Publications.pdf
3) Co-applicant CV - Benzing_CV.pdf
4) Co-applicant Publications List - Benzing_Publications.pdf
5) Co-applicant CV - Grunt_CV.pdf
6) Co-applicant Publications List - Grunt_Publications.pdf
7) Co-applicant CV - Roessler_CV.pdf
8) Co-applicant Publications List - Roessler_Publications.pdf
9) Co-applicant CV - Martin-Niedecken_CV.pdf
10) Co-applicant Publications List - Martin-Niedecken_Publications.pdf
11) Research Plan - Research Protocol.pdf
12) Point to point response - Point to Point Reply(2).pdf

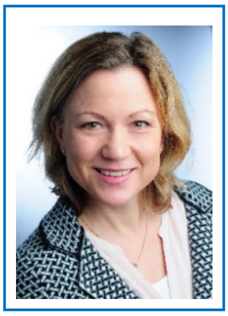

Prof. Dr. phil. Regula Everts

Personal Details

Function	Research Group Leader, Neuropsychology
Address	Pediatric Oncology/Hematology, Children's University Hospital, Inselspital, 3010 Bern
Date of Birth	12.8.1975
E-Mail	regula.everts@insel.ch

Education

2017	**Associate Professor**, Faculty of Medicine, University of Bern, Switzerland
2011	**Habilitation**, Venia Docendi for Neuropsychology, Faculty of Human Science, University of Bern, Switzerland
2006	**Dissertation**, Institute of Psychology, University of Bern, Switzerland
2003	**Master of Psychology**, Institute of Psychology, University of Bern
1996	**Master of Education**, Lehrerseminar Biel, Switzerland

Professional Experience

2018 – today	**Research Group Leader PICO-Study**, UDEM, University Hospital Bern, Switzerland
2016 – today	**Research Group Leader Brainfit-Study**, Hematology/Oncology, Children's University Hospital, Inselspital Bern , Switzerland
2012 – today	**Co-Research Group Leader HERO-Study**, Neuropediatrics, Children's University Hospital, Inselspital Bern, Switzerland
2009 – 2017	**Research Group Leader NEMO-Study**, Neuropediatrics, Children's University Hospital, Inselspital Bern, Switzerland
2009 – today	**Lecturer**, e.g. University of Bern, University of Zürich, University of Melbourne, Australia
2010 – 2014	**Post-doctoral Research Fellow**, Institute of Diagnostic and Interventional Neuroradiology, University Hospital Bern, Switzerland
2008	**Post-doctoral Research Fellow**, Murdoch Children's Research Institute, Melbourne, Australia
2008	**Honorary Research Fellow**, Australian Brain Research Institute, Melbourne, Australia
2007	**Clinical Neuropsychologist & Research Assistant**, Department of Neurosurgery, Neurocentre, Freiburg im Breisgau, Germany
2005 – 2007	**Clinical Neuropsychologist**, Neuropediatrics, Children's University Hospital, Inselspital Bern, Switzerland
2004 – 2006	**Doctoral Student**, Neuropediatrics, Children's University Hospital, Inselspital Bern, Switzerland
2002 – 2004	**Research Assistant**, KIKOM, Medical Institute, University of Bern, Switzerland
1997 – 2002	**High School Teacher**, various public schools, Switzerland

Grants and Fellowships (PI = Principle Investigator; Co-PI = Co-Investigator)

2021	**Stiftung Batzebär, Financial Support of the VR Study** (11'000.-)
2021	**Stiftung Rolf Gaillard, Financial Support of the PICO-Study**, PI, Round II (25`000.-)
2021	**Anna Müller Grocholski Stiftung**, PI, Financial Support of the HERO-Long Study (21'000.-)
2020	**Swiss National Science Foundation (SNSF)**, PI, Financial Support of the PICO-Study (519'000.-)
2020	**Stiftung Rolf Gaillard**, PI, Financial Support of the PICO-Study, Round I (25`000.-)
2020	**Krebsstiftung Thun-Berner Oberland**, PI, Financial Support of the Brainfit-Study (45`000.-)
2019	**Anna Müller Grocholski Stiftung**, PI, Financial Support of the Onset-Study (2600.-)
2019	**Stiftung Batzebär**, PI, Financial Support of the Onset-Study (2600.-)
2018	**Von Tobel Stiftung**, PI, Financial Support of the PICO-Study (150´000.-)
2018	**Beatrice Borer Stiftung**, PI, Financial Support of the Brainfit-Study (113´000.-)

2017	**Jacobs Foundation**, PI, Center for Cognition, Learning, and Memory, CCLM, University of Bern (150´000.-)
2016	**Beatrice Borer Stiftung**, PI, Financial Support of the NEMO longitudinal study (55´000.-)
2015	**Bangerter Rhyner Stiftung**, PI, Financial Support of the NEMO longitudinal study (50´000.-)
2014	**Swiss National Science Foundation**, Co-PI, Project Grant 124114, (2'200'000 CHF)
2014	**Stiftung Batzebär**, PI, Kinderklinik Bern, NEMO-Langzeitstudie (23´000.-)
2014	**Stiftung Vinetum**, PI, Financial Support of the HERO-Study (5´000.-)
2014	**Center for Cognition, Learning and Memory**, CCLM, University of Bern, PI, Financial Support of PhD student (17´000.-)
2013 – 2014	**Ambizione Fellowship**, PI, Prolongation, SNSF (178´000.-)
2013 - 2016	**Swiss National Science Foundation**,Co-PI, Project Grant 146894 (300´000.-)
2010	Grant for **International Short Visit**, SNSF, PI (8000.-)
2009 – 2012	**Ambizione Stipendium**, PI, SNSF (574´000.-)
2008 – 2009	**Fellowship for Prospective Researchers**, PI, SNSF (60´000.-)
2005 – 2009	**Swiss National Science Foundation**, Co-PI, Project Grant 112517 (173'000.-)

Scientific Awards

2016	**Research Prize** of the Adrian und Simone Frutiger Foundation
2012	**Scientific Award**, Annual Meeting of the Swiss Society of Pediatrics, Luzern
2012	**Scientific Award**, Meeting of the Swiss Society of Neuroradiology, Bern
2007	**Certificate of Merit**, 9th Congress of the European Society for MRI in Neuropediatrics, Tübingen
2007	**Scientific Award**, 33rd Meeting of the Society of Neuropediatrics, Passau
2005	**Cum Laude Award**, 30th Conference of the European Society of Neuroradiology, Barcelona
2003	**Certificate of Merit**, 31st Meeting of the Neuropediatric Society, Erlangen

Teaching

Since 2019	**Lecturer**, Institute of Psychology, University of Bern
Since 2017	**Lecturer in Neuropsychology**, Faculty of Medicine, University of Bern
Since 2011	**Supervision and scientific education of 10 PhD & 20 Master Students**
Since 2009	**Lecturer**, Schweizerischer Verein für Gedächtnistraining (SVGT)
2014 – 2015	**Mentor**, Swiss Academic Mentoring Program
2010 – 2011	**Lecturer in Developmental Psychology**, Faculty of Medicine, University of Bern
2008	**Lecturer in Developmental Neuropsychology**, Institute of Psychology, Monash University, Melbourne
2002 –2004	**Lecturer in Psychology**, Wirtschafts- und Kaderschule (WKS), Bern

Reviewer Activities

Public Library of Science, Epilepsia, Epilepsy & Behavior, Brain & Language, European Journal of Neurology, NeuroImage, European Journal of Developmental Psychology, Journal of Child Psychology and Psychiatry, Developmental Neuropsychology, Child Neuropsychology, Developmental Psychology, Journal of the International Neuropsychological Society, Brain Structure and Function, Biological Engeneering, Neuropsychologia, European Journal of Developmental Psychology, Assessment, NeuroImage Clincial and others

Expert Activities

European Research Council, Swiss National Science Foundation, French National Research Agency, Estonian Research Council, Elsevier Academic Press

Memberships

International Neuropsychological Society, INS, Federation for Cognitive Neuroscience, FLUX, Arbeitskreis Kinder & Jugend der Gesellschaft für Neuropsychologie, GNP, Schweizerischer Verein für Neuropsychologie im Kindes- und Jugendalter, VNKJ

Bern, 25.1.2022

Publications Prof. Dr. Regula Everts

Scientific articles as first or last author (peer-reviewed, last 5 years)

2021

Abgottspon, S., Thaqi, Q., Steiner, L., Slavova, N., Grunt, S., Steinlin, M., & **Everts, R.** (2021). Impact of Age at Pediatric Stroke on Long-term Cognitive Outcome. *Neurology*. epub ahead of print

Wehrle, F. M., Stöckli, A., Disselhoff, V., Schnider, B., Grunt, S., Mouthon, A. L., ... & **Everts, R**. (2021). Effects of Correcting for Prematurity on Executive Function Scores of Very Preterm Children at School Age. *The Journal of Pediatrics*. doi.org/10.1016/j.jpeds.2021.06.070

Siegwart, V., Steiner, L., Pastore-Wapp, M., Benzing, V., Spitzhuttl, J., Schmidt, M., ... & **Everts, R**. (2021). The Working Memory Network and Its Association with Working Memory Performance in Survivors of non-CNS Childhood Cancer. *Developmental neuropsychology*, *46*(3), 249-264.

Spitzhüttl J, Kronbichler M, Kronbichler L, Benzing B, Siegwart V, Pastore-Wapp M, Kiefer C, Slavova N, Grotzer M, Roebers C, Steinlin M, Leibundgut K, **Everts R** (2021). Cortical morphometry and its relationship with cognitive functions in children after non-CNS cancer. *Developmental Neurorehabilitation*, *24*(4), 266-275, doi10.1080/17518423.2021.1898059

Benzing, V., Siegwart, V., Spitzhüttl, J., Schmid, J., Grotzer, M., Roebers, C. M., **Everts R** & Schmidt, M. (2021). Motor ability, physical self-concept and health-related quality of life in pediatric cancer survivors. *Cancer medicine*. 10:1860–1871, doi10.1002/cam4.3750

Everts R, Muri R, Leibundgut K, Siegwart V, Wiest R, Steinlin M (2021). Fear and discomfort of children and adolescents during MRI – ethical consideration on research MRIs in children. *Pediatric Research*, 1-4

Siegwart V, Benzing V, Spitzhuettl J, Schmidt M, Grotzer M, Steinlin M, Leibundgut K, Roebers C, **Everts R** (2021). Cognition, Psychosocial Functioning, and Health-related Quality of Life among Childhood Cancer Survivors. *Neuropsychological Rehabilitation*, *46*(3), 249-264, doi10.1080/09602011.2020.1844243

Spitzhüttl J, Kronbichler M, Kronbichler L, Benzing B, Siegwart V, Pastore-Wapp M, Kiefer C, Slavova N, Grotzer M, Roebers C, Steinlin M, Leibundgut K, **Everts R** (2021). Impact of non-CNS childhood cancer on resting-state connectivity and its association with cognition. Brain and Behavior, *11*(1), e01931, doi10.1002/BRB3.1931

Abgottspon, S., Steiner, L., Slavova, N., Steinlin, M., Grunt, S., & **Everts, R.** (2021). Relationship between motor abilities and executive functions in patients after pediatric stroke. *Applied Neuropsychology: Child*, 1-11.

Steiner, L., Homan, S., **Everts, R**., Federspiel, A., Kamal, S., Rodriguez, J. A. D., ... & Grunt, S. (2021). Functional connectivity and upper limb function in patients after pediatric arterial ischemic stroke with contralateral corticospinal tract wiring. *Scientific reports*, *11*(1), 1-11.

2020

Steiner, L., Federspiel, A., Slavova, N., Wiest, R., Grunt, S., Steinlin, M., & **Everts, R**. (2020). Functional topography of the thalamo-cortical system during development and its relation to cognition. NeuroImage, 117361.

Benzing, V. J., Spitzhüttl, J. S., Siegwart, V., Schmid, J., Grotzer, M., Heinks Maldonado, T., ... & **Everts, R.** (2020). Effects of Cognitive Training and Exergaming in Pediatric Cancer Survivors–A Randomized Clinical Trial. Medicine & science in sports & exercise, 52(11), 2293.

Trepp R, Muri R, Abgottspon S, Bosanska L, Hochuli M, Slotboom J, Rummel C, Kreis R, **Everts R.**

(2020). Impact of phenylalanine on cognitive, cerebral, and neurometabolic parameters in adult patients with phenylketonuria (the PICO study): a randomized, placebo-controlled, crossover, noninferiority trial. Trials. Feb 13;21(1):178. doi: 10.1186/s13063-019-4022-z.

2019

Everts, R., Schöne, C. G., Mürner-Lavanchy, I., & Steinlin, M. (2019). Development of executive functions from childhood to adolescence in very preterm-born individuals-A longitudinal study. *Early human development*, 129, 45-51

Slavova, N., Shojai, M. P., **Everts, R**., Wiest, R., Steinlin, M., & Grunt, S. (2019). Is Asymmetry of the Pons Associated with Hand Function and Manual Ability after Arterial Ischemic Stroke in Children?. *Neuropediatrics*, *50*(03), 138-145.

Leistner, R., **Everts, R**., Federspiel, A., Kornfeld, S., Slavova, N., Steiner, L., ... & Grunt, S. (2019). Cerebral blood flow imbalance is associated with motor outcome after pediatric arterial ischemic stroke. *PloS one*, *14*(10).

2018

Mürner-Lavanchy I, Rummel C, Steinlin M, **Everts R**. (2018). Cortical morphometry and cognition in very preterm and term-born children at early school age. *Early Hum Dev.* 116:53-63.

Benzing V, Eggenberger N, Spitzhüttl J, Siegwart V, Pastore-Wapp M, Kiefer C, Slavova N, Grotzer M, Heinks T, Schmidt M, Conzelmann A, Steinlin M, **Everts R,** Leibundgut K. (2018) The Brainfit study: efficacy of cognitive training and exergaming in pediatric cancer survivors - a randomized controlled trial. BMC Cancer. 2018 Jan 3;18(1):18.

2017

Kornfeld S, Yuan R, Biswal BB, Grunt S, Kamal S, Delgado Rodríguez JA, Regényi M, Wiest R, Weisstanner C, Kiefer C, Steinlin M, **Everts R**. (2017). Resting-state connectivity and executive functions after pediatric arterial ischemic stroke. Neuroimage Clin. Oct 17;17:359-367.

Everts R, Mürner-Lavanchy I, Schroth G, Steinlin M. (2017). Neural changes after different memory training approaches in children born very preterm. Developmental Neurorehabilitation, 20(1) 14-24

Published books, book chapters and other articles

Lidzba K, **Everts R,** Reuner G. Neuropsychologie bei Kindern und Jugendlichen, ISBN: 9783801728359, 1. Aufl. 2019, 114 Seiten, Hogrefe Verlag

Everts R, Ritter B. Das Memo-Training. Memo, der vergessliche Elefant – Mit Gedächtnistraining spielerisch zum Lernerfolg, ISBN: 9783456856971, 2., überarb. u. erg. Aufl. 2017, 304 Seiten, Hogrefe Verlag

Everts R, Spitzhüttl J, Benzing V, Siegwart V, Wiest R, Grotzer M, Leibundgut K (2017). The Brainfit Study: Efficacy of Cognitive Training and Exergaming in Pediatric Cancer Survivors – a Randomized Controlled Trial. Schweizer Krebsbulletin / Bulletin suisse du cancer, 37(4), S. 373-374.

Everts R, & Ritter B (2013). Memo, der vergessliche Elefant: Mit Gedächtnistraining spielerisch zum Lernerfolg. Hans Huber Verlag, Bern

Dr. VALENTIN BENZING

CURRICULUM VITAE

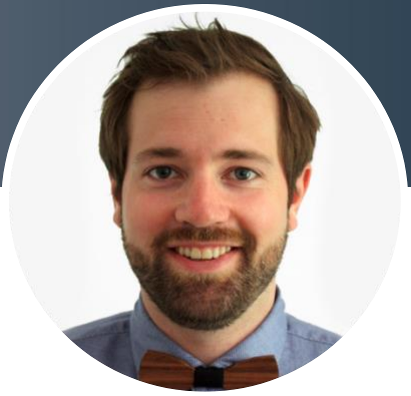

PERSONALIEN

Name	Dr. Valentin Benzing
Nationalität	Deutschland
Geburtsdatum	15.06.1988

KONTAKT

Murtenstrasse 215, CH-3027 Bern
M: +41 76 622 00 12
E: valentin.benzing@ispw.unibe.ch

AKADEMISCHER WERDEGANG

**seit 09/2021**	**Kommissarisch operative Leitung (krankheitsbedingt) der Abteilung für Sportpädagogik**, Institut für Sportwissenschaft, Universität Bern, Schweiz
**04/2020**	**Abschluss des Doktorandenausbildungsprogrammes der Swiss Graduate School for Cognition, Learning and Memory**, Universität Bern, Schweiz
**seit 05/2019**	**Assistent I (Post-doc) in der Abteilung Sportpädagogik**, Institut für Sportwissenschaft, Universität Bern, Schweiz (70%)
**seit 10/2019**	**Postdoc in der Abteilung für Hämatologie und Onkologie**, Universitätsklinik für Kinderheilkunde, Inselspital, Universität Bern, Schweiz (30%)
**04/2019**	**Promotion**, Institut für Sportwissenschaft, Universität Bern, Schweiz<br>Doktorarbeit: „Acute and Chronic Physical Activity and Executive Functions in Children and Adolescents: Benefits of Exergaming in ADHD?"
**09/2017-10/2017**	**Forschungsaufenthalt am „Graduate Institute of Athletics and Coaching Science" bei Prof. Dr. Yu-Kai Chang**, National Taiwan Sport University, Taoyuan City, Taiwan
**07/2017-08/2017**	**Forschungsaufenthalt am „Emotions and Quantitative Psychophysiology Lab" bei Prof. Dr. Julian Thayer**, Ohio State University, Columbus, OH, USA
**05/2015 -05/2019**	**Assistent III am Institut für Sportwissenschaft (Abteilung I)**, Universität Bern, Schweiz
**11/2014-03/2015**	**Wissenschaftlicher Assistent in der Abteilung systemische Neurowissenschaften der Psychopathologie, Zentrum für Translationale Forschung,** Universitätsklinik für Psychiatrie und Psychotherapie, Universität Bern, Schweiz
**09/2012-03/2015**	**Master of Science in Psychologie**, Spezialisierung: Klinische Psychologie und Gesundheitspsychologie, Universität Fribourg, Schweiz
**09/2010-09/2013**	**Bachelor of Science in Psychologie**, Universität Fribourg, Schweiz

GUTACHTERTÄTIGKEITEN

BMC Pediatrics; Educational Psychology Review; Frontiers in Psychology; Frontiers in Psychiatry; Games for Health; German Journal of Exercise and Sport Research; International Journal of Environmental Research and Public Health; International Review of Sport and Exercise Psychology; Journal of Aging and Physical Activity; Journal of Clinical Medicine; Journal of Physical Activity and Health; Journal of Sport and Health Science; Medicine and Science in Sports & Exercise; Mental Health and Physical Activity; Pediatrics; Psychology of Sport & Exercise; Psychological Science; Scandinavian Journal of Medicine & Science in Sports; Sport, Exercise, and Performance Psychology; Sports Medicine

ABSCHLÜSSE UND EXAMINA

**02/2016**	Good Clinical Practice Aufbaukurs – Sponsor Investigator
**10/2015**	Good Clinical Practice
**04/2014**	Ausbildung in „NEUROGES coding system for hand movements" (Modul 1)
**09/2012-04/2013**	Ausbildung zum Lerntrainer, Freiburger Training für Leistungsstress

WISSENSCHAFTLICHE AUSZEICHNUNG

Dissertationspreis der Sportwissenschaftlichen Gesellschaft der Schweiz (SGS) 2020

Benzing, **V**. Acute and Chronic Physical Activity and Executive Functions in Children and Adolescents: Benefits of Exergaming in ADHD?

Nominiert für den PhD Academy Award 2020 des British Journal of Sports Medicine

Benzing, **V.** (2020). Exergaming–a physical activity intervention to benefit children with ADHD? (PhD Academy Award). *British Journal of Sports Medicine*. bjsports-2019-101920, https://doi.org/10.1136/bjsports-2019-101920

Anna Müller Grocholski Preis der Schweizerischen Gesellschaft für Kinder mit Behinderung

Benzing, **V.**, Spitzhüttl, J., Siegwart, V., Kiefer, C., Slavova, N., Grotzer, M., Steinlin, M. S., Leibundgut, K., Everts, R., Schmidt, M. (2018). *Executive functions and motor abilities in pediatric cancer survivors – on the importance of motor coordination.* 18. Symposium der Schweizerischen Akademie für Kinder mit Behinderung. Zürich, Schweiz.

NASPSPA Outstanding Student Paper Award

Benzing, **V.**, & Schmidt, M. (2017). *Exergaming intervention to foster executive functions in children with attention deficit hyperactivity disorder: preliminary results from a clinical trial*. 50. Jahrestagung der nordamerikanischen Gesellschaft für Sportpsychologie (NASPSPA). San Diego, USA.

3. Preis: SGS Young Investigator Award

Benzing, **V.**, & Schmidt, M. (2018). *Exergaming intervention to foster executive functions in children with attention deficit hyperactivity disorder: Results from a randomized controlled trial*. 10. Jahrestagung der Sportwissenschaftlichen Gesellschaft der
Schweiz (SGS). Magglingen, Schweiz.

3. Preis FEPSAC Young Researcher Award

Benzing, **V.**, Spitzhüttl, J., Siegwart, V., Kiefer, C., Slavova, N., Grotzer, M., Steinlin, M. S., Leibundgut, K., Everts, R., Schmidt, M. (2019). *Executive functions, motor abilities and quality of life in pediatric cancer survivors – the crucial role of motor coordination*. **15. Europäischer Kongress für Sportpsychologie (FEPSAC). Münster, Deutschland.**

AUSGEWÄHLTE DRITTMITTELPROJEKTE

**2021**	**UniBE Initiator Grant: Benefits of exergaming for children with ADHD**
Gesuchsstellender:	Valentin Benzing
Gesuchsbewilligung:	Universität Bern, Bern, Schweiz: 28´910 CHF
**2020**	**Bewegungsbarrieren bei Kindern und Jugendlichen: Eine sportpsychologische Untersuchung im Rahmen des sozial-kognitiven Prozessmodell**
Gesuchsstellender:	Valentin Benzing
Gesuchsbewilligung:	Stiftung Suzanne und Hans Biäsch: 16´754 CHF
**2020**	**Sportliche Aktivität und soziale Interaktion – Einfluss auf die Emotionsregulation und soziale Kognition**
Gesuchsstellender:	Valentin Benzing
Gesuchsbewilligung:	Philosophisch-humanwissenschaftliche Fakultät, Universität Bern: Hilfsassistentin für 6 Monate zu 20%
	Burger Stiftung: 3´000 CHF
**2016-2018**	**Exergames und ADHS**
Gesuchsstellende:	Mirko Schmidt & Valentin Benzing
Gesuchsbewilligung:	Stiftung Suzanne und Hans Biäsch: 26´000 CHF
**2016-2018**	**Sportintervention bei Kindern mit ADHS**
Gesuchsstellende:	Mirko Schmidt & Valentin Benzing
Gesuchsbewilligung:	Stiftung Hans und Annelies Swierstra: 4´656 CHF
**2015-2018**	**Efficacy of Cognitive and Physical Remediation Training in Pediatric Cancer Survivors**
Gesuchsstellende:	Prof. Dr. med. Kurt Leibundgut, Dr. Theda Heinks, Prof. Dr. Achim Conzelmann, Prof. Dr. Maja Steinlin, Prof. Dr. med. Michael Grotzer, Dr. med. Christian Weisstanner, Prof. Dr. Barbara Plecko
Gesuchsbewilligung:	Fondation Gaydoul, Krebsliga Schweiz, Dietmar Hopp Stiftung, Berner Stiftung für krebskranke Kinder, Hans und Annelies Swierstra Stiftung: 750´000 CHF (Involviert als Mitarbeiter und Gesuchssteller bei der Hans und Annelies Swierstra Stiftung)
**2015-2018**	**Workshops und Reisestipendien**
Gesuchsstellende:	Valentin Benzing
Gesuchsbewilligung:	Mittelbauvereinigung Universität Bern & Center for Cognition, Learning & Memory: 19´436 CHF

PUBLICATIONS

SCIENTIFIC ARTICLES

Benzing, V., Nosrat, S., Aghababa, A., Barkoukis, V., Bondarev, D., Chang, Y.-K., Cheval, B., Çiftçi, M. C., Elsangedy, H. M., Guinto, M. L. M., Huang, Z., Kopp, M., Kristjánsdóttir, H., Kuan, G., Mallia, L., Rafnsson, D., Oliveira, G. T. A., Pesola, A. J., Pesce, C., … Brand, R. (2021). Staying active under restrictions: Changes in type of physical exercise during the initial COVID-19 lockdown. *International Journal of Environmental Research and Public Health*, *18*(22), 12015. https://doi.org/10.3390/ijerph182212015

Benzing, V., Siegwart, V., Spitzhüttl, J., Schmid, J., Grotzer, M., Roebers, C. M., Steinlin, M., Leibundgut, K., Everts, R., & Schmidt, M. (2021). Motor ability, physical self-concept and health-related quality of life in pediatric cancer survivors. Cancer Medicine, cam4.3750. https://doi.org/10.1002/cam4.3750

Chueh, T.-Y., Hsieh, S.-S., Tsai, Y.-J., Yu, C.-L., Hung, C.-L., **Benzing, V.**, Schmidt, M., Chang, Y.-K., Hillman, C. H., & Hung, T.-M. (2021). Effects of a single bout of moderate-to-vigorous physical activity on executive functions in children with attention-deficit/hyperactivity disorder: A systematic review and meta-analysis. *Psychology of Sport and Exercise*, 102097. https://doi.org/10.1016/j.psychsport.2021.102097

Stodden, D., Lakes, K. D., Côté, J., Aadland, E., **Benzing, V.**, Brian, A., Draper, C. E., Ekkekakis, P., Fumagalli, G., Laukkanen, A., Mavilidi, M. F., Mazzoli, E., Neville, R. D., Niemistö, D., Rudd, J., Sääkslahti, A., Schmidt, M., Tomporowski, P. D., Tortella, P., Vazou, S., & Pesce, C. (2021). Exploration: an overarching focus for holistic development. *Brazilian Journal of Motor Behavior*, *15*(5), 301–320. https://doi.org/10.20338/bjmb.v15i5.254

Pesce, C., Ballester, R., & **Benzing, V.** (2021). *Giving physical activity and cognition research ‘ some soul ’: focus on children and adolescents*. *1*. https://doi.org/10.21134/eurjhm.2021.47.1

Pesce, C., Vazou, S., **Benzing, V.**, Álvarez-Bueno, C., Anzeneder, S., Mavilidi, M.-F., Leone, L., & Schmidt, M. (2021). Effects of chronic physical activity on cognition across the lifespan: a systematic meta-review of randomized controlled trials and realist synthesis of contextualized mechanisms. *International Review of Sport and Exercise Psychology*. https://doi.org/10.1080/1750984X.2021.1929404

Siegwart, V., Steiner, L., Pastore-Wapp, M., **Benzing, V**., Spitzhuttl, J., Schmidt, M., Kiefer, C., Slavova, N., Grotzer, M., Roebers, C., Steinlin, M., Leibundgut, K., & Everts, R. (2021). The working memory network and its association with working memory performance in survivors of non-CNS childhood cancer. Developmental Neuropsychology, 46(3), 249–264. https://doi.org/10.1080/87565641.2021.1922410

Spitzhüttl, J. S., Kronbichler, M., Kronbichler, L., **Benzing, V.**, Siegwart, V., Pastore-Wapp, M., Kiefer, C., Slavova, N., Grotzer, M., Roebers, C. M., Steinlin, M., Leibundgut, K., & Everts, R. (2021). Impact of non-CNS childhood cancer on resting-state connectivity and its association with cognition. Brain and Behavior, September 2020, 1–16. https://doi.org/10.1002/brb3.1931

Spitzhüttl, J. S., Kronbichler, M., Kronbichler, L., **Benzing, V.**, Siegwart, V., Schmidt, M., Pastore-Wapp, M., Kiefer, C., Slavova, N., Grotzer, M., Steinlin, M., Roebers, C. M., Leibundgut, K., & Everts, R. (2021). Cortical morphometry and its relationship with cognitive functions in children after non-CNS cancer. Developmental Neurorehabilitation. https://doi.org/10.1080/17518423.2021.1898059

Benzing, V., Spitzhüttl, J., Siegwart, V., Schmid, J., Grotzer, M., Heinks, T., Roebers, C. M., Steinlin, M., Leibundgut, K., Schmidt, M., & Everts, R. (2020). Effects of cognitive training and exergaming in pediatric cancer survivors – a RCT. *Medicine & Science in Sports & Exercise*. Published ahead of Print (5), 2293–2302. https://doi.org/10.1249/MSS.0000000000002386

Benzing, V. (2020). Exergaming-a physical activity intervention to benefit children with ADHD? (PhD Academy Award). *British Journal of Sports Medicine*, 1–2. https://doi.org/10.1136/bjsports-2019-101920

Siegwart, V., **Benzing, V.,** Spitzhuettl, J., Schmidt, M., Grotzer, M., Steinlin, M., Leibundgut, K., Roebers, C., & Everts, R. (2020). Cognition, psychosocial functioning, and health-related quality of life among childhood cancer survivors. Neuropsychological Rehabilitation, 0(0), 1–24. https://doi.org/10.1080/09602011.2020.1844243

Benzing, V., & Schmidt, M. (2019). The effect of exergaming on executive functions in children with ADHD: A randomized clinical trial. *Scandinavian Journal of Medicine & Science in Sports*, 29(8), sms.13446. https://doi.org/10.1111/sms.13446

Egger, F., **Benzing, V.**, Conzelmann, A., & Schmidt, M. (2019). Boost your brain, while having a break! The effects of long-term cognitively engaging physical activity breaks on children’s executive functions and academic achievement. *PLOS ONE*, 14(3), e0212482. http://doi.org/10.1371/journal.pone.0212482

Schmidt, M., **Benzing, V.**, Wallman-Jones, A., Mavilidi, M.-F., Lubans, D. R., & Paas, F. (2019). Embodied learning in the classroom: Effects on primary school children’s attention and foreign language vocabulary learning. *Psychology of Sport and Exercise*, 43, 45–54. http://doi.org/10.1016/j.psychsport.2018.12.017

PUBLICATIONS

Benzing, V., & Schmidt, M. (2018). Exergaming for children and adolescents: strengths, weaknesses, opportunities and threats. *Journal of Clinical Medicine*, 7(11), 422. http://doi.org/10.3390/jcm7110422

Benzing, V., Chang, Y.-K., & Schmidt, M. (2018). Acute physical activity enhances executive functions in children with ADHD. *Scientific Reports*, 8(1), 12382. http://doi.org/10.1038/s41598-018-30067-8

Benzing, V., Schmidt, M., Jäger, K., Egger, F., Conzelmann, A., & Roebers, C. M. (2018). A classroom intervention to improve executive functions in late primary school children: Too 'old' for improvements? *British Journal of Educational Psychology*, 1–14. http://doi. org/10.1111/bjep.12232

Benzing, V., Eggenberger, N. Spitzhüttl, J. S., Siegwart, V., Pastore-Wapp, M., Kiefer, C., Slavova, N. B., Grotzer, M., Heinks, T., Schmidt, M., Conzelmann, A., Steinlin, M., Everts, R. & Leibundgut, K. (2018). The Brainfit study: efficacy of cognitive training and exergaming in pediatric cancer survivors - a randomized controlled trial. *BMC Cancer*, 18, 18. 10.1186/s12885-017-3933-x

Benzing, V., & Schmidt, M. (2017). Cognitively and physically demanding exergaming to improve executive functions of children with attention deficit hyperactivity disorder: a randomised clinical trial. *BMC Pediatrics*, 1–8. doi.: 10.1186/s12887-016-0757-9

Everts, R., Spitzhüttl, J., **Benzing, V.**, Siegwart, V., Wiest, R., Grotzer, M. & Leibundgut, K. (2017). The Brainfit Study: Efficacy of Cognitive Training and Exergaming in Pediatric Cancer Survivors – a Randomized Controlled Trial. *Schweizer Krebsbulletin*, 37, 373-374.

Schmidt, M., Egger, F., **Benzing, V.**, Jäger, K., Conzelmann, A., Roebers, C.M., & Pesce, C. (2017). Disentangling the relationship between children's motor ability, executive function and academic achievement. *PLOS ONE*, 12(8): e0182845. https://doi.org/10.1371/ journal.pone.0182845

Mueller, D., Khalesi, Z., **Benzing, V.**, Castiglione, C., & Roder, V. (2017). Does Integrated Neurocognitive Therapy (INT) reduce severe negative symptoms in schizophrenia outpatients? Schizophrenia Research, 10.1016/j.schres.2017.01.037

POPULAR SCIENCE ARTICLES

Anzeneder, S. & **Benzing, V.** (2019). Macht sportliche Betätigung schlau?! *Zeitschrift für Sportpsychologie*, 26(1). 10.1026/1612-5010/a000252

Benzing, V., & Schmidt, M. (2017). Move! – Because exercise can boost your brainpower. *Frontiers for young minds*. Doi.: 10.3389/frym.2017.00022

BOOK CHAPTERS

Benzing, V., Pesce, C. & Wegner, M. (in press). Ganzheitlich gesund aufwachsen: Wie der Zusammenhang von körperlicher Aktivität mit motorischer und kognitiver Entwicklung zum Aufbau körperlich-psychischer Gesundheit beiträgt [Growing up healthy: how the link between physical activity and motor and cognitive development contributes to building physical-mental health]. In S. Klatt, B. Strauß (Eds.), Kognition und Motorik – Sportpsychologische Grundlagenforschung und Anwendungen im Sport. Hogrefe: Göttingen.

Schmidt, M., Egger, F., Anzeneder, S. & **Benzing, V.** (2020). Acute cognitively challenging physical activity to promote children's cognition. In R. Bailey, J. A. Agans, J. Cote, A. Daly-Smith, P. Tomporowski (Eds.), *Physical activity and sport during the first ten years of life*. London: Routledge.

CURRICULUM VITAE

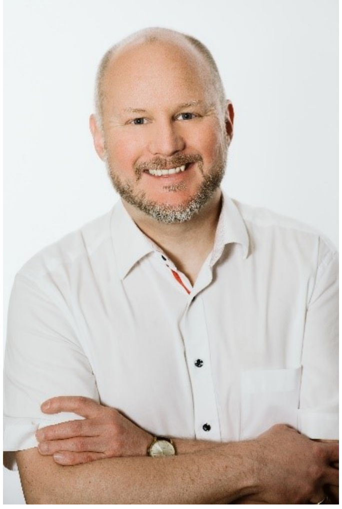

Personal Data

Name	Sebastian Grunt
Nationality	Swiss
Date of birth	25. January 1972
Place of birth	Liestal
Tel.	++ 41 31 632 94 24
E-mail	sebastian.grunt@insel.ch

Current Position

Senior physician (since 2013) for pediatric rehabilitation medicine at the Division of Neuropediatrics, Development and Rehabilitation, University Children's Hospital, Inselspital, 3010 Bern.

Diploma and Certifications

1997	Diploma of Physician, Swiss confederation
2003	MD, Faculty of Medicine, University of Zürich
2006	Pediatric and adolescent medicine specialist title
2011	PhD, Faculty of Medicine, VU Medical Centre, Amsterdam, the Netherlands
2018	Habilitation, Faculty of Medicine, Berne, Switzerland

Training

1991 to 1997	Study in Medicine in Basel, Paris (F) and Brisbane (Aus)
1998	Junior Doctor; Swiss Army
1999	Geriatrics and internal medicine; Felix Platter Hospital, Basel
2000 to 2001	Child and adolescent psychiatry; Child Psychiatry Service, Solothurn
2001 to 2002	Neonatal medicine; University Children's Hospital, Berne
2003 to 2004	General pediatrics; Clinic for Pediatrics, Canton Hospital, Fribourg
2004 to 2006	General pediatrics and neuropediatrics; University Children's Hospital, Berne
2002 to 2003	General pediatrics; University Children's Hospital, Berne
2006 to 2008	Clinical and research fellowship (PhD) in pediatric rehabilitation medicine; Department of Rehabilitation Medicine, VU Medical Center, Amsterdam, the Netherlands

2008 to 2011	Completing training in pediatric rehabilitation medicine as senior registrar, Department of Neuropediatrics, Development and Rehabilitation; University Children's Hospital, Berne
2011 to 2013	Post doc research fellowship (50%) and consultant for pediatric rehabilitation medicine (50%). Division of Neuropediatrics, Development and Rehabilitation; University Children's Hospital, Berne

Memberships and work on the Executive Board

Since 2003	Swiss Society of Pediatrics (membership)
Since 2006	European Academy of Childhood Disability (membership)
Since 2008	Swiss Society of Neuropediatrics (membership)
Since 2008	Swiss Neonatal Network and Follow Up Group (board membership)
Since 2008	Swiss Neuropediatric Stroke Registry (board membership)
Since 2010	Swiss Academy of Childhood Disability (current Co-President)
Since 2011	Swiss Society of Neurorehabilitation (membership)
Since 2016	Swiss Cerebral Palsy Registry (current president)

Current Clinical Work

Head of the pediatric early rehabilitation group and head of the outpatient clinic for development and rehabilitation. Responsible for the rehabilitation treatment of inpatients and outpatients with acute and chronic physical and/or mental disability. Responsible for the follow up of children born prematurely. Acute care and follow-up of patients with neuro-oncological diseases. Pediatric neurology call duty.

Scientific Work

Sebastian Grunt's research focuses on neuroplasticity after early brain lesions in relation to motor function, diagnosis and the treatment of movement disorders, early diagnosis of movement disorders and motor rehabilitation of children. Sebastian Grunt is involved in various registries and epidemiological research of children with neurological conditions, such as the Swiss Neuropediatric Stroke Registry, the Swiss Cerebral Palsy Registry and the Swiss Neonatal Network and Follow Up Group.

Reviewing Activities and **Editorial Duties**

Academic Editor PLOS One. Reviewer for various neurological and rehabilitation medicine journals such as Developmental Medicine and Child Neurology, Neuropediatrics, Disability and Rehabilitation, American Journal of Physical Medicine and Rehabilitation, Gait & Posture, Neurorehabilitation & Neural Repair, Acta Paediatrica, Brain Imaging & Behavior, New England Journal of Medicine, Human Brain Mapping, BMC Pediatrics, Child Neuropsychology.

Sebastian Grunt, 29.1.2022

Publications Sebastian Grunt

Original Articles of the last 5 years

2021

Abgottspon S, Steiner L, Slavova N, Steinlin M, **Grunt S**, Everts R. Relationship between motor abilities and executive functions in patients after pediatric stroke. Appl Neuropsychol Child. 2021 May 27:1-11. doi: 10.1080/21622965.2021.1919111. Online ahead of print.

Abgottspon S, Thaqi Q, Steiner L, Slavova N, **Grunt S**, Steinlin M, Everts R. Impact of Age at Pediatric Stroke on Long-term Cognitive Outcome. Neurology. 2021 Dec 16:10.1212/WNL.0000000000013207. doi: 10.1212/WNL.0000000000013207. Online ahead of print.

Simon-Martinez C, Kamal S, Frickmann F, Steiner L, Slavova N, Everts R, Steinlin M, **Grunt S**. Participation after childhood stroke: Is there a relationship with lesion size, motor function and manual ability? Eur J Paediatr Neurol. 2021 Nov;35:16-26. doi: 10.1016/j.ejpn.2021.09.010. Epub 2021 Sep 23.

Steiner L, Homan S, Everts R, Federspiel A, Kamal S, Rodriguez JAD, Kornfeld S, Slavova N, Wiest R, Kaelin-Lang A, Steinlin M, **Grunt S**. Functional connectivity and upper limb function in patients after pediatric arterial ischemic stroke with contralateral corticospinal tract wiring. Sci Rep. 2021 Mar 9;11(1):5490. doi: 10.1038/s41598-021-84671-2.

Steiner L, Federspiel A, Jaros J, Slavova N, Wiest R, Steinlin M, **Grunt S**, Everts R. Cerebral blood flow and cognitive outcome after pediatric stroke in the middle cerebral artery.Sci Rep. 2021 Sep 30;11(1):19421. doi: 10.1038/s41598-021-98309-w.

Grunt S. Looking beyond motor function-adaptive behaviour in children with unilateral spastic cerebral palsy. Eur J Paediatr Neurol. 2022 Jan;36:A1. doi: 10.1016/j.ejpn.2021.12.016. Epub 2021 Dec 30.

Wehrle FM, Stöckli A, Disselhoff V, Schnider B, **Grunt S**, Mouthon AL, Latal B, Hagmann CF, Everts R. Effects of Correcting for Prematurity on Executive Function Scores of Children Born Very Preterm at School Age. J Pediatr. 2021 Nov;238:145-152.e2. doi: 10.1016/j.jpeds.2021.06.070. Epub 2021 Jul 1.

Zürcher K, Dupont C, Weber P, **Grunt S**, Wilhelm I, Eigenmann DE, Reichmuth ML, Fankhauser M, Egger M, Fenner L. Use and caregiver-reported efficacy of medical cannabis in children and adolescents in Switzerland. Eur J Pediatr. 2022 Jan;181(1):335-347. doi: 10.1007/s00431-021-04202-z. Epub 2021 Jul 26.

2020

Steiner L, Federspiel A, Slavova N, Wiest R, **Grunt S**, Steinlin M, Everts R.Functional topography of the thalamo-cortical system during development and its relation to cognition. Neuroimage. 2020 Dec;223:117361

Mackay MT, Slavova N, Pastore-Wapp M, **Grunt S**, Stojanovski B, Donath S, Steinlin M. Pediatric ASPECTS predicts outcomes following acute symptomatic neonatal arterial stroke. Neurology. 2020 Mar 24;94(12)

Picotti E, Bechtel N, Latal B, Borradori-Tolsa C, Bickle-Graz M, **Grunt S**, Johnson S, Wolke D, Natalucci G; Swiss Neonatal Network & Follow-Up Group. Performance of the German version of the PARCA-R questionnaire as a developmental screening tool in two-year-old very preterm infants. PLoS One. 2020 Sep 3;15(9):e0236289.

Wiedemann A, Pastore-Wapp M, Slavova N, Steiner L, Weisstanner C, Regényi M, Steinlin M, **Grunt S**; Swiss Neuropediatric Stroke Registry Group. Impact of stroke volume on motor outcome in neonatal arterial ischemic stroke. Eur J Paediatr Neurol. 2019 Nov 1. pii: S1090-3798(19)30395-2. doi: 10.1016/j.ejpn.2019.10.006. [Epub ahead of print] Eur J Paediatr Neurol. 2020 Mar;25:97-105

2019

Adams, M., Berger, T.M., Borradori-Tolsa, C., Bickle-Graz, M., **Grunt, S**., Gerull, R., Bassler, D., Natalucci, G., 2019. Association between perinatal interventional activity and 2-year outcome of Swiss extremely preterm born infants: a population-based cohort study. BMJ Open. 2019 Mar 15;9(3):e024560

Fink, M., Slavova, N., **Grunt, S**., Perret, E., Regényi, M., Steinlin, M., Bigi, S., 2019. Posterior Arterial Ischemic Stroke in Childhood: Clinical Features and Neuroimaging Characteristics. Stroke. 2019 Sep;50(9):2329-2335

Caspar-Teuscher, M., Studer, M., Regényi, M., Steinlin, M., **Grunt, S**., Bigi, S., Mori, A.C., Datta, A., Fluss, J., Hackenberg, A., 2019. Health related quality of life and manual ability 5 years after neonatal ischemic stroke. Eur J Paediatr Neurol. 2019 Sep;23(5):716-72

Leistner R., Everts R., Federspiel A., Kornfeld S., Slavova N., Steiner L., Wiest R., Steinlin M., **Grunt S**., 2019 Cerebral blood flow imbalance is associated with motor outcome after pediatric arterial ischemic stroke. PLoS One. 2019 Oct 11;14(10):e0223584

Pittet-Metrailler, M.P., Mürner-Lavanchy, I., Adams, M., Bickle-Graz, M., Pfister, R.E., Natalucci, G., **Grunt, S**., Borradori Tolsa, C., 2019. Neurodevelopmental outcome at early school age in a Swiss national cohort of very preterm children. Swiss Med Wkly. 2019 Jun 2;149:w20084

Slavova, N., Shojai, M.P., Everts, R., Wiest, R., Steinlin, M., **Grunt, S**., 2019. Is Asymmetry of the Pons Associated with Hand Function and Manual Ability after Arterial Ischemic Stroke in Children? Neuropediatrics. 2019 Jun;50(3):138-145

2018

Chan, A., Lensing, A.W., Kubitza, D., Brown, G., Elorza, D., Ybarra, M., Halton, J., **Grunt, S**., Kenet, G., Bonnet, D. Clinical presentation and therapeutic management of venous thrombosis in young children: a retrospective analysis. Thromb J. 2018 Nov 1;16:29

Kornfeld, S., Yuan, R., Biswal, B.B., **Grunt, S**., Kamal, S., Rodríguez, J.A.D., Regényi, M., Wiest, R., Weisstanner, C., Kiefer, C., 2018. Resting-state connectivity and executive functions after pediatric arterial ischemic stroke. Neuroimage Clin. 2017 Oct 17;17:359-367

2017

Datta, A.N., Furrer, M.A., Bernhardt, I., Hüppi, P.S., Borradori-Tolsa, C., Bucher, H.U., Latal, B., **Grunt, S**., Natalucci, G., Group, G., 2017. Fidgety movements in infants born very preterm: predictive value for cerebral palsy in a clinical multicentre setting. Dev Med Child Neurol. 2017 Jun;59(6):618-624

Grunt, S., Newman, C.J., Saxer, S., Steinlin, M., Weisstanner, C., Kaelin-Lang, A., 2017. The mirror illusion increases motor cortex excitability in children with and without hemiparesis. Neurorehabil Neural Repair. 2017 Mar;31(3):280-289

Weisstanner, C., Saxer, S., Wiest, R., Kaelin-Lang, A., Newman, C.J., Steinlin, M., **Grunt, S**., 2017. The neuronal correlates of mirror illusion in children with spastic hemiparesis: a study with functional magnetic resonance imaging. Swiss Med Wkly. 2017 Feb 17;147

Verma, R.K., Keller, D., **Grunt, S**., Bigi, S., Weisstanner, C., Wiest, R., Gralla, J., Hutter, D., Wagner, B., 2017. Decreased oxygen saturation levels in neonates with transposition of great arteries: Impact on appearance of cerebral veins in susceptibility-weighted imaging. Sci Rep. 2017 Nov 13;7(1):15471

Review Articles

Tscherter A, Kuehni C, **Grunt S**. Kinder mit Cerebralparese: neue Kollaboration zur Optimierung der Versorgung. Kinderärzte Schweiz 02.2018

Case reports

Waldmeier-Wilhelm, S., Studer, M., Weisstanner, C., Heinks, T., **Grunt, S**., Steinlin, M., 2019. Mild traumatic brain injury: striking postconcussion symptoms due to inadequate management. Pediatr Emerg Care. 2019 Jun;35(6):e99-e103

Carlhan-Ledermann A, Laubscher B, Steinlin M, Ulrich CT, Verma RK, Rizzi M, Maduri R, **Grunt S**. Spinal epidural hematoma without significant trauma in children: two case reports and review of the literature. BMC Pediatr. 2020 Feb 19;20(1):77

Jochen Rößler CURRICULUM VITAE January 2022

(1) PERSONAL INFORMATION

Name, Firstname: Rößler, Jochen
Languages: German, English, French (all spoken and written), Italian and Greek (both very good command)
Professional address: Head, Pediatric Hematology/Oncology Unit
University Hospital of Berne, Inselspital
Freiburgstrasse 15, 3010 Bern
e-mail: jochen.roessler@insel.ch

(2) PERSONAL STATEMENT ON CONTRIBUTION TO PROJECT

Senior Pediatric Hematologist/Oncologist with interest in clinical and preclinical drug development for cancer. Pre-clinical research activity in the field of angiogenesis, tyrosine kinase inhibitors, drug delivery and nanomedicine. Clinical research focuses on personalized medicine for vascular malformations and pediatric tumors. Competent knowledge in designing and executing national and international phase I-IV clinical studies in pediatric patients.

(3) PREVIOUS AND PRESENT APPOINTMENTS

Since 11/2017 **Head of Division of Pediatric Hematology/Oncology, University Hospital of Berne, Switzerland**

- Head of a team of 6 full time pediatric hematology/oncology consultants running a unit of 8 beds, an outpatient and day care clinic
- Offering diagnostics and treatment for the complete spectrum of pediatric hematology and oncology diseases, including hemophilia as well as stem cell apharesis and high dose chemotherapy
- Head of a research lab with a senior scientist, a post doc and two PhD students
- Teaching activity at the University Bern Medical School

2008–2017 **Consultant Physician in Pediatric Hematology/Oncology, University Children's Hospital Freiburg, Germany**

- Pediatric hematologist and oncologist taking care of in- and outpatient treatment including representation of Pediatric Oncology in the Comprehensive Cancer Center Freiburg
- Medical and operational responsibility for clinical research and clinical studies with focus on early phase (I/II) studies
- Director of the interdisciplinary vascular anomaly center Freiburg for diagnostics and therapies of children and adults with vascular malformations and tumors
- Member of the clinical trial committee of the European ITCC consortium
- Director of a preclinical research team focusing on new therapy approaches for pediatric solid tumors (angiogenesis, tyrosine kinase inhibitors, drug delivery, nanomedicine)
- (Principal) Investigator and care for patients with experimental therapies in cancer and dermatology

Since 09/2008 **Adjunct Professor in Pediatric Hematology/Oncology, University of Freiburg, Germany**

- Lectures and seminars in Pediatric Hematology/Oncology
- Bed side teaching
- Supervision of clinical and pre-clinical (laboratory) MD thesis
- Principle Investigator of the Sperman Graduate School

2014 – 2016 **Consultant Physicians in Pediatric Hematology/Oncology**, **University Hospital Montpellier (CHU), France**

- Direction of the pediatric hematology/oncology ward, including hematopoietic stem cell transplantation
- Clinical focus and responsibility for brain tumor and vascular anomaly patients
- Investigator in clinical trials phase I-IV

2014 **University Children's Hospital Freiburg, Germany, Deputy Director of the Department of Pediatric Hematology /Oncology**

- Medical and administrative responsibilities of the Department, including budget supervision and member of the centers managing board

2006 – 2008 **University Paris XI and Institute Gustave Roussy, France, Associate Professor in Oncology and Clinical Pharmacology**

- Principle investigator in the pre-clinical research group "Pharmacology and New Treatments in Cancer"
- Investigator in clinical trials phase I-II

2001 – 2006 **University Children's Hospital Freiburg, Germany**
2005 – 2006 **Consultant Physicians in Pediatric Hematology/Oncology**
2001 – 2006 **Clinical Fellow in Pediatric Hematology/Oncology**
1994 – 2001 **University Children's Hospital, Essen, Germany**
1994 – 2001 **Resident in General Pediatrics**
1996 – 1998 **Research Fellow in Pediatric Hematology/Oncology**

(4) ACADEMIC AND PROFESSIONAL QUALIFICATIONS

2019 *Associate Professor*, University of Berne, Switzerland
2010 *Adjunct Professor*, University of Freiburg, Germany
2007 *Board certificate*, Pediatric Hematology-Oncology
2006 *Associate Professor*, University of Paris XI, France
2005 *Habilitation (Privatdozent)*, University of Freiburg, Germany
2002 *Board certificate*, General Pediatrics
1994 *Medical Doctor*, University of Heidelberg, Germany

(5) MEMBERSHIP IN PROFESSIONAL SOCIETIES

- Swiss Pediatric Oncology Group (SPOG), since 2017
- International Society on the Studies of Vascular Anomalies (ISSVA), since 2006
- European Society for Pediatric Haematology and Immunology (ESPHI), since 2000
- Gesellschaft für Pädiatrische Onkologie und Hämatologie (GPOH), since 1999
- Deutsche Gesellschaft für Kinderheilkunde und Jugendmedizin (DGKJ), 1994

(6) ACTIVE RESEARCH GRANTS

1. Cancer league of both Basel (KLbB-5060-02-2020): How to mitigate the consequences of cancer and cancer related treatments in childhood cancer survivors (CHF 45'800; 10/2020 – 09/2022: Co-applicant)
2. Cancer Research Switzerland (KFS 5027-02-2020): Prospective Multicentre Cohort Study for Diagnosing Cardiac Dysfunction in Swiss Childhood Cancer Survivors (CHF 336'950; 01/2021-12/2023; Co-applicant)
3. Swiss National Fonds (CRSII5_193694/1): Disease-targeted next-generation sequencing panel (VASCSequ) for detection of somatic-mosaic mutations in congenital vascular malformations to enable further advances in personalized therapeutic decision making (CHF 230'000 of CHF 2'255'011 total budget; 01/2021 – 12/2024; Co-applicant)
4. Cancer Research Switzerland (KFS-4157-02-2017): Pulmonary dysfunction after childhood cancer: assessing early stage disease (Phase 2) (CHF 369'153; 10/2021 – 09/2024; Co-applicant)

JOCHEN RÖSSLER
SELECTED PUBLICATIONS JANUARY 2017-JANUARY 2022

ORIGINAL ARTICLES (PEER-REVIEWED JOURNALS)

2021

1. **Rössler JK**, Baumgartner I, Kremer Hovinga JA. EHA Endorsement of the Second International Guidelines for the Diagnosis and Management of Hereditary Hemorrhagic Telangiectasia. *HemaSphere, 2021, 5(11), e647.*
2. He J, Zhang Y, Zhu J, Tan H, **Rössler J.** Editorial: Molecular Diagnostics of Pediatric Cancer. *Front Oncol, 2021, Oct 11;11:777662.*
3. Bernhard SM, Tuleja A, Laine JE, Haupt F, Häberli D, Hügel U, **Rössler J**, Schindewolf M, Baumgartner I. Clinical presentation of simple and combined or syndromic arteriovenous malformations. *Journal of Vascular Surgery, 2021, Oct 11:S2213-333X(21)00508-4.*
4. Holm A, Te Loo M, Schultze K, Leo S, Päivi CV, Baselga E, Duignan S, Dvorakova V, Irvine AD, Boon LM, Vikkula M, Ghaffarpour N, Niemeyer CM, **Rössler J***, Kapp FG*. Efficacy of Sirolimus in Patients Requiring Tracheostomy for Life-Threatening Lymphatic Malformation of the Head and Neck: A Report From the European Reference Network. *Frontiers in Pediatrics,* 2021, 9(697960), p. 697960.
5. Koenig C, Ammann RA, Kuehni CE, **Roessler J**, Brack E. Continuous recording of vital signs with a wearable device in pediatric patients undergoing chemotherapy for cancer-an operational feasibility study. *Supportive care in cancer (2021), 29(9), 5283-5292.*
6. Liniger S, Dantonello T, Diepold M, Aebi C, Brodard J, Kremer Hovinga JA, **Rössler JK**, Kartal-Kaess M. Severe acquired purpura fulminans in a child. *Archives of Disease in Childhood, 2021, Aug 20:archdischild-2021-322379.*
7. Bernhard SM, Adam L, Atef H, Häberli D, Bramer WM, Minder B, Döring Y, Laine JE, Muka T, **Rössler JK**, Baumgartner I. A systematic review of the safety and efficacy of currently used treatment modalities in the treatment of patients with PIK3CA-related overgrowth spectrum. *Journal of Vascular Surgery: Venous and lymphatic disorders,* 2021, Aug 3:S2213-333X(21)00391-7.
8. **Rössler J**, Baselga E, Davila V, Celis V, Diociaiuti A, El Hachem M, Mestre S, Haeberli D, Prokop A, Hanke C, Loichinger W, Quéré I, Baumgartner I, Niemeyer CM, Kapp FG. Severe adverse events during sirolimus "off-label" therapy for vascular anomalies. *Pediatric Blood & Cancer (2021), 68(8), e28936.*
9. Bell, LM, Holm A, Matysiak U, Driever W, **Rössler J**, Schanze D, Wieland I, Niemeyer CM, Zenker M, Kapp FG. Functional assessment of two variants of unknown significance in TEK by endothelium-specific expression in zebrafish embryos. *Human Molecular Genetics, 2021, Jul 12:196.*
10. Karow A, Haubitz M, Oppliger Leibundgut E, Helsen I, Preising N, Steiner D, Dantonello TM, Ammann RA, **Roessler J**, Kartal-Kaess M, Röth A, Baerlocher GM. Targeting Telomere Biology in Acute Lymphoblastic Leukemia. *International Journal of Molecular Sciences, 2021, 22(13). Jun 22;22(13):6653.*
11. Brill R, Uller W, Huf V, Müller-Wille R, Schmid I, Pohl A, Häberle B, Perkowski S, Funke K, Till AM, Lauten M, Neumann J, Güttel C, Heid E, Ziermann F, Schmid A, Hüsemann D, Meyer L, Sporns PB, Schinner R, Schmidt VF, Ricke J, **Rössler J**, Kapp FG, Wohlgemuth WA, Wildgruber M. Additive value of transarterial embolization to systemic sirolimus treatment in kaposiform hemangioendothelioma. *International Journal of Cancer. 2021, 148(9), 2345-2351.*
12. Waespe N, Belle FN, Redmond S, Schindera C, Spycher BD, **Rössler J**, Ansari M, Kuehni, CE. Cancer predisposition syndromes as a risk factor for early second primary neoplasms after childhood cancer - A national cohort study. *European Journal of Cancer. 2021, 145, 71-80.*
13. Lavieri L, Koenig C, Bodmer N, Agyeman PKA, Scheinemann K, Ansari M, **Roessler J**, Ammann RA. Predicting fever in neutropenia with safety-relevant events in children undergoing chemotherapy for cancer: The prospective multicenter SPOG 2015 FN Definition Study. *Pediatr Blood Cancer. 2021 Dec;68(12):e29253.*
14. De Gaudenzi M, Kremer Hovinga JA, **Rössler J**, Kaess M, Kartal-Kaess M. Psychische Gesundheit bei chronischen Erkrankungen am Beispiel der Hämophilie. *Paediatrica. 2021, 32(2), 13-16.*
15. Quéré I, Stasi E, Mestre S, **Roessler J**, Roccatello D, Moffatt C. International Camps for Children with Lymphedema and Lymphatic Anomalies: When Education Links with Psychosocial Research *Lymphat Res Biol. 2021 Feb;19(1):36-40.*
16. Dantonello TM, Kartal-Kaess M, Aebi C, Suter-Riniker F, Busch JD, Kubetzko S, Bourquin JP, **Roessler J**. SARS-CoV-2 Infection During Induction Chemotherapy in a Child With High-risk T-Cell Acute Lymphoblastic Leukemia *J Pediatr Hematol Oncol. 2021 Aug 1;43(6):e804-e807.*

2020

17. Janzarik WG, Sander M, **Rößler J**, Sommerlade L, Allignol A, Reinhard M. Cerebral Autoregulation and Neurovascular Coupling after Craniospinal Irradiation in Long-Term Survivors of Malignant Pediatric Brain Tumors of the Posterior Fossa. *Neuropediatrics. 2020 Oct 16. Online ahead of print.*

18. Schindera C, Kuehni CE, Pavlovic M, Haegler-Laube ES, Rhyner D, Waespe N, **Roessler J**, Suter T, von der Weid NX. Single-Center Cohort Study for Diagnosing Preclinical Cardiac Dysfunction in Swiss Childhood Cancer Survivors: A Study Protocol. *JMIR Res Protoc. 2020 Apr 7. doi: 10.2196/17724.*

19. Alijaj N, Moutel S, Gouveia ZL, Gray M, Roveri M, Dzhumashev D, Weber F, Meier G, Luciani P,**Rössler JK**, Schäfer BW, Perez F, Ber-nasconi M. Novel FGFR4-Targeting Single-Domain Antibodies for Mul-tiple Targeted Therapies against Rhabdomyosarcoma. *Cancers. 2020; 12:3313.*

20. Brill R, Uller W, Huf V, Müller-Wille R, Schmid I, Pohl A, Häberle B, Per-kowski S, Funke K, Till AM, Lauten M, Neumann J, Güttel C, Heid E, Ziermann F, Schmid A, Hüsemann D, Meyer L, Sporns PB, Schinner R, Schmidt VF, Ricke J**, Rössler J**, Kapp FG, Wohlgemuth WA, Wildgruber M. Additive value of transarterial embolization to systemic sirolimus treatment in kaposiform hemangioendothelioma. *Int J Cancer. 2020 Nov 24. doi: 10.1002/ijc.33406. Online ahead of print.*

21. Koenig C, Bodmer N, Agyeman PKA, Niggli F, Adam C, Ansari M, Eisenreich B, Keller N, Leibundgut K, Nadal D, **Roessler J**, Scheinemann K, Simon A, Teuffel O, von der Weid NX, Zeller M, Zimmermann K, Ammann RA. 39·0°C versus 38·5°C ear temperature as fever limit in children with neutropenia undergoing chemotherapy for cancer: a multicentre, cluster-randomised, multiple-crossover, non-inferiority trial. *The Lancet Child & Adolescent Health 2020; 4 (7) : 495–502.*

22. Dantonello TM, Kartal-Kaess M, Aebi C, Suter-Riniker F, Busch JD, Kubetzko S, Bourquin JP, **Roessler J**. SARS-CoV-2 Infection During Induction Chemotherapy in a Child With High-risk T-Cell Acute Lymphoblastic Leukemia (T-ALL). *J Pediatr Hematol Oncol. 2020 Sep 11. Online ahead of print.*

23. Daurat M , Nguyen C , Dominguez Gil S , Sol V , Chaleix V , Charnay C , Raehm L , El Cheikh K , Morère A , Bernasconi M , Timpanaro A , Garcia M , Cunin F , **Roessler J*** , Durand JO* , Gary-Bobo M*. The mannose 6-phosphate receptor targeted with porphyrin-based periodic mesoporous organosilica nanoparticles for rhabdomyosarcoma theranostics. *Biomater Sci. 2020 Jul 7;8(13):3678-3684.*

24. Léauté-Labrèze C, Baselga Torres E, Weibel L, Boon LM, El Hachem M, van der Vleuten C, **Roessler J**, Troilius Rubin A. The Infantile Hemangioma Referral Score: A Validated Tool for Physicians. *Pediatrics. 2020 Apr;145(4).*

25. Morland B, Kepak T, Dallorso S, Sevilla J, Murphy D, Luksch R, Yaniv I, Bader P, **Rößler J**, Bisogno G, Maecker-Kolhoff B, Lang P, Zwaan CM, Sumerauer D, Kriván G, Bernard J, Liu Q, Doyle E, Locatelli F. Plerixafor combined with standard regimens for hematopoietic stem cell mobilization in pediatric patients with solid tumors eligible for autologous transplants: two-arm phase I/II study (MOZAIC). *Bone Marrow Transplant. 2020 Sep;55(9):1744-1753.*

26. Sparber-Sauer M, Koscielniak E, Vokuhl C, Schmid I, Bien E, Seitz G, Hallmen E, von Kalle T, Scheer M, Münter M, Bielack SS, Niggli F, Ljungman G, Fuchs J, Hettmer S, **Rössler J**, Klingebiel T; CWS Study Group. Endothelial cell malignancies in infants, children and adolescents: Treatment results of three Cooperative Weichteilsarkom Studi*engruppe (CWS) trials and one registry. Pediatr Blood Cancer. 2020 Mar;67(3):e28095.*

2019

27. Lagrèze WA, Joachimsen L, Gross N, Taschner C, **Rössler J**. Sirolimus-induced regression of a large orbital lymphangioma. *Orbit. 2019 Feb;38(1):79-80.*

2018

28. Blesinger H, Kaulfuß S, Aung T, Schwoch S, Prantl L, **Rößler J**, Wilting J, Becker J. PIK3CA mutations are specifically localized to lymphatic endothelial cells of lymphatic malformations. *PLoS One. 2018 Jul 9;13(7).*

2017

29. Ferlemann FC, Menon V, Condurat AL, **Rößler J**, Pruszak J. Surface marker profiling of SH-SY5Y cells enables small molecule screens identifying BMP4 as a modulator of neuroblastoma differentiation. *Sci Rep. 2017 Oct 19;7(1):13612*.

30. Hettmer S, Andrieux G, Hochrein J, Kurz P, **Rössler J**, Lassmann S, Werner M, von Bubnoff N, Peters C, Koscielniak E, Sparber-Sauer M, Niemeyer C, Mentzel T, Busch H, Boerries M. Epithelioid hemangioendotheliomas of the liver and lung in children and adolescents. *Pediatr Blood Cancer. 2017 Dec;64(12).*

31. Seiffert A, Schneider M, **Roessler J**, Larisch K, Pfeiffer D. Incidence, Treatment Patterns, and Health Care Costs of Infantile Hemangioma: Results of a Retrospective German Database Analysis. *Pediatr Dermatol. 2017 Jul;34(4):450-457*
32. **Rössler J**, Geiger J, Földi E, Adams DM, Niemeyer CM. Sirolimus is highly effective for lymph leakage in microcystic lymphatic malformations with skin involvement. *Int J Dermatol. 2017 Apr;56(4):e72-e75.*

Curriculum Vitae – Dr. Anna Lisa Martin-Niedecken

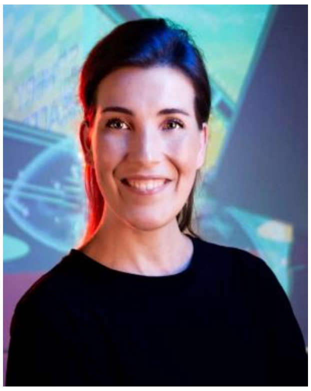

Professional Competences:

- **Director of Institute**
  Department of Design, Institute for Design Research
  Zurich University of the Arts, Pfingstweidstrasse 96, Postfach, 8031 Zürich
  anna.martin@zhdk.ch

- **Senior Researcher, R&D Project Manager & Lecturer**
  Department of Design, Subject Area in Game Design
  Zurich University of the Arts, Pfingstweidstrasse 96, Postfach, 8031 Zürich

- **Professor for esports Management**, HAM University of Applied Management

- **CEO, Co-Founder & Member Board of Directors**, Sphery AG

Personal Profiles:
Google Scholar Profile, ResearchGate academic profile, and LinkedIn business profile.

Education

2021	Dr. rer. nat. Sports Science (Serious Games, HCI in Sports), Technical University of Darmstadt, Interdisciplinary DFG-Graduate School Topology of Technology & Institute of Sports Science, Germany
2009	M.A. (Magistra Artium) Sports Science & Pedagogy, Technical University of Darmstadt, Institute of Sports Science, Germany

Advanced training in: Good Clinical Practice Module 1-3 (Clinical Trial Center, University Hospital Zurich), Research Management (TU Darmstadt), Working Techniques in Science (TU Darmstadt), and Entrepreneurship (ZHdK-Inkubator and Z-Kubator).

Employment history including current position(s)

02/2021 – p/t	Director of the Institute of Design Research, Department of Design, Zurich University of the Arts
09/2021 – p/t	Professor for esports Management, Faculty of Sports Management, University of Applied Management
01/2013 – p/t	Senior Researcher, R&D Project Manager & Lecturer, Subject Area in Game Design, Department of Design, Zurich University of the Arts
03/2018 – p/t	CEO & Co-Founder, Sphery AG (SpinOff of the Zurich University of the Arts)
01/2009 – 12/09	Head of Medical Strengthening Therapy, Kieser Training Wiesbaden
12/2005 – 12/08	Instructor, Kieser Training Wiesbaden
08/2003 – 07/04	Voluntary Social Year, Orthopeadic Clinic, St. Josefs-Hospital Wiesbaden

Approved research projects (as Main Applicant)

2021 – 2023:	**DIZH Innovation Project** (408'062 CHF, as co-applicant and Co-PI), Project: "ExerUp! – *Design and Evaluation of a Digital Exergame-Based Solution for Effective and Attractive Sports Rehabilitation*"
2021 – 2023:	**AAL Grant** (1'181'193.30 €, as main applicant and Co-PI), Project: "ExerG: An innovative digital solution to individually improve physical and cognitive functions using an exergame (video game-based) training in an ecologically valid and safe setting for the geriatric population".
2020 – 2022:	**Innosuisse Grant** (652'794.96 CHF, as main applicant), Innovation Project: "Home-Based Fitness Gaming for Diabetes Prevention: Effects of Game-Based Training on Cardio-Metabolic and Cerebrovascular Health in Individuals at Risk of Type 2 Diabetes".
2019 – 2021:	**Innosuisse Grant** (636'393 CHF, as main applicant and PI), Follow-up Project: "A playful exergame training in multiple sclerosis patients".
2018 – 2019:	**CTI Grant** (291'615 CHF, as main applicant and PI), Project: "A playful motor-cognitive training intervention in multiple sclerosis patients".
2015 – 2018:	**Stiftung Mercator Schweiz** (330'000 CHF, as main applicant), Project: "Design with Social Impact", Master of the Department of Design, Zurich University of the Arts.
2014 – 2015:	**Sportfonds of the Kanton Zurich**, Swisslos (20'000 CHF, as main applicant and PI), Project: (Pilot) "Psychophysiologisch adaptives Exergame Fitnesstraining für Kinder und Jugendliche".
2014 – 2017:	**EU-Project** (968'697 €, Senior Researcher) "JamToday – The European Game Jam Learning Hub"

2013 – 2016: **Mäxi Stiftung** (Senior Researcher) "IMIC – Innovative Movement Therapy in Childhood"

2011: **Guest Researcher**, Sensory-Motor Systems Lab, ETH Zurich

2015, 2016, 2017, 2018, 2020: **R&D Grants of the Department of Design**, Zurich University of the Arts

Teaching activities (analogue and digital)
- Since 2020: Training Module on "Stärkung physischer Leistungsressourcen im E-Sport", ESBD, GER
- Since 2020: Teaching Modules in eSports Management (e.g., "Foundations of Sports Science in eSports", "Foundations of Sports Management and Scientific Work", University of Applied Management, GER
- Since 2018: Annual CAS Module: "Motivation Design and Serious Games" (CAS series "Design Technology"), Zurich University of the Arts
- Since 2015: Recurring Advanced Training Modules: "Gamification" and "Gamify Agency"
- Since 2014: Annual Teaching Nodule: "Interdisciplinary Game Development and Research: Serious & Applied Games", Zurich University of the Arts
- Since 2014: Recurring Workshops and Consulting on HCI, Gamification and (Serious) Game Design for interdisciplinary professionals in the field of health, training, sports, education and management.
- Since 2013: Recurring Workshops for schools and in advanced training programs with a specific focus on science, technology and knowledge transfer

Supervision of junior researchers at graduate and postgraduate level
- 3 doctoral (ETH, USZ, TU Chemnitz), 9 master (ZHdK, ETH, HAM), and 7 bachelor students (ZHdK & ETH)

Memberships in scientific societies, panels, boards, etc.
- Associate Member of the Serious Games group, etit, TU Darmstadt
- Founding Member and Member of the Board of the RAL Quality Mark for Serious Games e.V.
- Chairwoman and Founding Member of International Mixed Reality Sports Association (XRS)
- Expert and Member of DIN (German Institute for Standards) SPEC 91380 – Serious Games Metadata Format
- Expert, Entrepreneur, Consultant: "WTT – Serious Games", Science and Technology Tansfer, Initiative funded by European Funding for Regional Development; Technical University of Darmstadt, GER)
- PC Member of the Annual Joint Conference of Serious Games
- Associate Chair of ACM CHI Conference, ACM CHI Play Conference
- Professional Member of ACM Association for Computing Machinery
- Member Board of Directors, Sphery AG

Scientific reviewing activities
- Reviewer for ACM CHI Conference, ACM CHI Play Conference, Intern. Journal of Games for Health, ACM IMWUT Journal, Elsvesier "Heliyon" Journal, Joint Conference on Serious Games, GamiFIN.

Organisation of conferences
- Co-Curator and Organizer of the Conference "Interactive Experiences: Playgrounds of Life. Animacy, Embodiment and Empathy in the Context of Game Design, 02.07.2020, Zurich, CH
- Co-Curator and Organizer of the Conference "Interactive Experiences: Crossing Boundaries for Serious Reasons" at Ludicious – Zürich Game Festival, 26.1.2017, Zurich, CH
- Associate Chair & Member of Program Committee: ACM CHI 19 and ACM CHI Play 2020
- Session Chair of the Auxiliary Track - Vision Papers at the 2015 IEEE Conference on Computational Intelligence and Games (CIG 15), Tainan, TWN
- Member of Program Committee, Annual Joint Conference on Serious Games

Prizes, awards, honors, fellowships (Selection)
- Nominee (Shortlist) for the Women in Games Global Award 2021 in the category "Hall of Fame Awards" & "Video Games Advocacy Awards"
- Winner (Gold) of German Design Award 2021
- Digital Shaper 2020 (Creatives; 100 Digital Shaper of Switzerland)
- Winner of FIBO Innovation & Trend Award 2019 and 2020
- Winner (Gold) German Innovation Award 2020 "Gold"
- ISPO 2020 Brandnew Selection
- Best Paper Award JCSG 2018
- TEDx Speaker at TEDxZurich "Connections" 2018
- Nominated for Best Video Award at CHI 2018
- Winner of the Best Paper Award Joint Conference on Serious Games 2018
- Special Recognition: Reviewer for ACM CHI Play Conference 2018 and ACM CHI 2020
- Winner of the Falling Walls Lab Audience Award 2016
- DFG PhD-Scholarship (100'000 €)

Publication List (Selection: 2015 – 2020) – Dr. Anna Lisa Martin-Niedecken

Peer-reviewed publications

- Kriglstein, S., **Martin-Niedecken, A.L.**, Spjut, J., Damen, N.B., Turkay, S., Drachen, A. (accepted) Esports Meets Human Computer Interaction. Interactions
- Kircher, E., Ketelhut, S., Ketelhut, K., Röglin, L., **Martin-Niedecken, A.L.**, Hottenrott, K. and Ketelhut, R.G. (2022). Acute Effects of Heart Rate-Controlled Exergaming on Vascular Function in Young Adults. Games for Health Journal Research, Development, and Clinical Applications, Volume 11, Number 1.
- Ketelhut, S., Ketelhut, R.G., Kircher, E., Röglin, L., Hottenrott, K., **Martin-Niedecken, A.L.**, and Ketelhut, K. (2022). Gaming instead of training? Exergaming induces high-intensity exercise stimulus and reduces cardiovascular reactivity to cold pressor test. Frontiers in Cardiovascular Medicine, section Hypertension
- Kircher, E., Ketelhut, S., Ketelhut, K., Röglin, L., Hottenrott, K., **Martin-Niedecken, A. L.**, & Ketelhut, R. G. (2022). A Game-Based Approach to Lower Blood Pressure? Comparing Acute Hemodynamic Responses to Endurance Exercise and Exergaming: A Randomized Crossover Trial. International Journal of Environmental Research and Public Health, 19(3), 1349.
- Röglin, L. Ketelhut, S., Ketelhut, K., Kircher, E., Ketelhut, R., **Martin-Niedecken, A.L.**, Hottenrott, K., and Stoll, O. (2021). Adaptive High-Intensity Exergaming – The More Enjoyable Alternative to Conventional Training Approaches Despite Working Harder. Games for Health Journal Research, Development, and Clinical Applications, Volume 10, Number 6.
- Ketelhut, S., **Martin-Niedecken, A.L.**, Zimmermann, P. and Nigg, C.R. (2021). Physical activity and health promotion in esports and gaming – discussing unique opportunities for an unprecedented cultural phenomenon. Front. Sports Act. Living - Movement Science and Sport Psychology
- **Martin-Niedecken, A.L.**, Schwarz, T. and Schättin, A. (2021). Comparing the Impact of Different Heart-Rate-Based In-Game Adaptations in an Exergame-Based Functional High-Intensity Interval Training on Training Intensity and Experience in Healthy Young Adults. Front. Psychol.
- Ketelhut, S., Röglin, L., Kircher, E., **Martin-Niedecken, A.L.**, Ketelhut, R., Hottenrott, K. and Ketelhut, K. (2021). The New Way to Exercise? Evaluating an Innovative Heart-rate-controlled Exergame. International Journal of Sports Medicine 2021, 42, pp. 1–6
- Schättin, A., Häfliger, St., Früh, B., Meyer, A., Böckler, S., Hungerbühler, Y., de Bruin, E.D., Frese, S., Steinlin Egli, R., Götz, U., Bauer, R., and **Martin-Niedecken A.L.** (2021). «Senso Exploria»: Design and Evaluation of User-Centered Exergames for Multiple Sclerosis Patients. JMIR Serious Games
- Ketelhut, S. , Bodman, A., Kubica, C., **Martin-Niedecken, A.L.**, and Nigg, C.R. (2021). Health Status In Competitive E-sports Athletes- A Cross Sectional Study. ERN Conference 2021.
- Nigg, C.R., **Martin-Niedecken, A.L.**, Bodman, A., Kubica, C., Zimmermann, P. Schmidt, M., and Ketelhut, S. (2021). Rationale and Perspective of the eSports Health And PErformance Network. ERN Conference 2021.
- Kriglstein, S., **Martin-Niedecken, A.L.**, Turmo Vidal, L., Klarkowski, M., Rogers, K., Turkay, S., Seif El-Nasr, M., Marquez Segura, E., Drachen, A., and Perttu Hämäläinen (2021). The Present and Future of eSports in HCI. ACM CHI 2021
- Márquez Segura, E., Rogers, K., **Martin-Niedecken, A.L.**, Niedecken, S., Turmo Vidal, L. (2021) Exploring the Design Space of Immersive Social Fitness Games: The ImSoFit Games Model. ACM CHI 2021.
- Hug, D., Papetti, S. **Martin-Niedecken, A.L.**, Rogers, K. and Graf, E. (2021). Sound Design for Exergames - A design research pilot project, Research Symposium of Zurich University of the Arts.
- **Martin-Niedecken A.L.**, Mahrer, A., Rogers, K., De Bruin, E.D., and Schättin, A. (2020). «HIIT» the ExerCube: Comparing the Effectiveness of Functional High-Intensity Interval Training in Conventional vs. Exergame-Based Training. Frontiers in Computer Science.
- **Martin-Niedecken A.L.** and Schättin A. (2020) Let the Body'n'Brain Games Begin: Toward Innovative Training Approaches in eSports Athletes. Front. Psychol. 11:138. doi: 10.3389/fpsyg.2020.00138
- Patibanda, R., Semertzidis, N., Scary, M., La Delfa, J., Andres, J., Baytaş, M.A., **Martin-Niedecken, A.L.**, et al. (2020). Motor Memory in CHI, In Proceedings of the 2020 CHI Conference on Human Factors in Computing Systems. ACM.
- Ketelhut, S. , **Martin-Niedecken, A.L.**, Kircher, E., Röglin, L., Ketelhut, R.G., Hottenrott, K., Ketelhut, K. (2020). Evaluating the exercise intensity during an exergame session in the ExerCube. ECSS Congress Sevilla 2020. Sevilla, ESP.
- Zwingmann, K., **Martin-Niedecken, A. L**., Schättin, A. and Voelcker-Rehage, C. (2020). Short physical-cognitive (work) breaks to improve cognitive flexibility via exergaming. ECSS Congress Sevilla 2020. Sevilla, ESP.
- **Martin-Niedecken A.L.**, Márquez Segura E., Rogers K., Niedecken, S., and Turmo Vidal L. (2019). Towards Socially Immersive Fitness Games: An Exploratory Evaluation Through Embodied Sketching. In Proceedings of the Annual Symposium on Computer-Human Interaction in Play Companion Extended Abstracts (pp. 525-534). ACM.
- **Martin-Niedecken A.L.**, Rogers K., Turmo Vidal L., Mekler E. D., and Márquez Segura E. (2019). ExerCube vs. Personal Trainer: Evaluating a Holistic, Immersive, and Adaptive Fitness Game Setup. In ACM Conference on Human Factors in Computing Systems Proceedings (CHI 2019), ACM, New York, NY, USA.
- Kocher, M., **Martin-Niedecken, A.L.**, Li, Y., Kinzelbach, W., Wang, H., Bauer, R. and Lunin, L. (2019). "Save the Water" – A China Water Management Game Project. In Proceedings of the 3rd International GamiFIN Conference, GamiFIN 2019. 1-12, Springer.

- Schmidt, M., Benzing, V., Bonadimann, P., **Martin-Niedecken, A.L.** (2019). Kognitiv-anspruchsvolle sportliche Aktivität und kognitive Leistung: Profitieren denkfaule Menschen weniger?. In: Proceedings der 11. SGS-Jahrestagung «Sport und Gehirn», Freiburg. CH.
- Schmidt, M., Benzing, V., Bonadimann, P., **Martin-Niedecken, A.L.** (2019). Cognitively engaging physical activity and cognitive performance: Is there a dose response relationship?. In: Proceedings of Health and Active Children, Verona, ITA.
- **Martin-Niedecken A.L.**, Mekler E.D. (2018). The ExerCube: Participatory Design of an Immersive Fitness Game Environment. In Göbel, S., Garcia-Agundez, A., Tregel, Th., Ma, M., Baalsrud Hauge, J., Oliveira, M., Marsh, T., Caserman, P. (eds) Serious Games. JCSG 2018. Lecture Notes in Computer Science, vol 11243. Springer, Cham.
- **Martin-Niedecken, A.L.** (2018). Designing for Bodily Interplay: Engaging with the Adaptive Social Exertion Game "Plunder Planet". In Proceedings of the 17th ACM Conference on Interaction Design and Children (IDC'18), pp. 19-30.
- McCaskey, M.A., Schättin, A., **Martin-Niedecken, A.L.**, and de Bruin, E.D. (2018). "Making More of IT: Enabling Intensive Motor Cognitive Rehabilitation Exercises in Geriatrics Using Information Technology Solutions," BioMed Research International, vol. 2018, Article ID 4856146, 17 pages.
- Florian "Floyd" Mueller, Josh Andres, Joe Marshall, Dag Svanæs, m. c. schraefel, Kathrin Gerling, Jakob Tholander, **Anna Lisa Martin-Niedecken**, Elena Márquez Segura, Elise van den Hoven, Nicholas Graham, Kristina Höök, and Corina Sas. 2018. Body-centric computing: results from a weeklong Dagstuhl seminar in a German castle. Interactions 25, 4 (June 2018), 34-39. DOI 10.1145/3215854
- Mela Kocher, Livio Lunin, Wolfgang Kinzelbach, René Bauer, Yu Li, Haijing Wang, and **Anna Lisa Martin-Niedecken** (2018). Save the Water! Serious Game for Water Management in Chinese Farmers. In Proceedings of 3rd Gamification and Serious Games Symposium (GSGS'18).
- Kinzelbach, W., Li, Y., **Martin-Niedecken, A.L.**, Bauer, R., Lunin, L., Kocher, M. and Wang, H. (2018). Developing a board game for chinese farmers. In Geophysical Research Abstracts, Vol. 20, EGU2018-15336-1, EGU General Assembly 2018.
- Leu, A., **Martin-Niedecken, A.L.**, Wepf, H., Berger, F., Weber, A. & Frech, M. (2018). Serious Games für Young Carers. Digitale Spiele als Mediator zwischen Patienten, Angehörigen und Fachpersonen. Workshopleitung. *1. Nationaler Patientenkongress.* Bern, 14.09.2018.
- **Martin-Niedecken, A.L.** & Götz, U. (2017). Go with the Dual Flow: Evaluating the Psychophysiological Adaptive Fitness Game Environment "Plunder Planet". In Alcañiz M., Göbel S., Ma M., Fradinho Oliveira M., Baalsrud Hauge J., Marsh T. (Eds). Serious Games. JCSG 2017. Lecture Notes in Computer Science, vol. 10622, pp. 32-43, Springer International Publishing. DOI: 10.1007/978-3-319-70111-0_4
- **Martin-Niedecken, A.L.** (2017). Exploring Spatial Experiences of Children and Young Adolescents While Playing the Dual Flow-based Fitness Game "Plunder Planet". In Proceedings of the International Conference on Computer-Human Interaction Research and Application (CHIRA '17), pp. 218-229, SCITEPRESS – Science and Technology Publications. ISBN: 978-989-758-267-7. DOI: 10.5220/0006587702180229
- **Martin-Niedecken, A.L.** (2017). Plunder Planet – A Psychophysiologically Adaptive Fitness Game Environment for Children and Young Adolescents. In Proceedings of the 2nd Gamification & Serious Game Symposium (GSGS '17), pp. 21-22. ISBN: 978-2-940387-19-9. ISSN: 2297-914X.
- **Martin-Niedecken, A.L.** & Götz, U. (2016). Design and Evaluation of a dynamically adaptive Fitness Game Environment for Children and Young Adolescents. In Proceedings of the ACM SIGCHI Annual Symposium on Computer-Human Interaction in Play (CHI PLAY '16), pp. 205-212, ACM: New York, NY, USA. ISBN 978-1-4503-4458-6. DOI 10.1145/2968120.2987720
- **Martin-Niedecken, A.L.**, Bauer, R., Mauerhofer, R. & Götz, U. (2015). RehabConnex - A Middleware for the Flexible Connection of Multimodal Game Applications with Input Devices used in Movement Therapy and Physical Exercising. In Proceedings of the 2015 IEEE Conference on Computational Intelligence and Games (CIG), pp. 496-502, IEEE Xplore Digital Library. ISBN 978-1-4799-8621-7 (Print). DOI 10.1109/CIG.2015.7317671
- **Martin-Niedecken, A.L.** (2015). Computer Games - Genres, Devices und deren (Transfer-) Effekte. In Könecke, Th., Preuß, H. & Schöllhorn, W. I. (Eds.), Moving Minds - Crossing Boundaries in Sport Science. In Abstracts of the 22. dvs Hochschultags in Mainz, p. 50, Feldhaus: Edition Czwalina (Schriften der Deutschen Vereinigung für Sportwissenschaft: Band 251). ISBN 978-3-88020-692-8 (Print).

Books & Book Chapters

- Dörner, R., **Martin-Niedecken, A.L.**, Kocher, M., Baranowski, T., Kickmeier-Rust, M., Göbel, S., Wiemeyer, J. & Gebelein, P. (2016). Contributing Disciplines. In Dörner, R., Göbel, S., Effelsberg, W. & Wiemeyer, J. (Eds.). Serious Games – Foundations, Concepts and Practice, pp. 35-55, Springerlink, ISBN 978-3-319-40611-4 (Print), 978-3-319-40612-1 (Online).
- Göbel S, Caserman P, Hansen J, Bruder R, Abels S, Behrmann M, Hauge JB, Limpach O, Reichert M, Junge J, Vogt S, Raubold O, Nowarra N, Kruse R, Roller W, Streicher A, Walter T, Wacker M, Flory C, Hugo OA, Grimm P, Dörner R, Breitlauch L, Steinicke M, Bergmeyer W, Thiele-Schwez M, Althoff US, Wunsch H, Mildner P, Hofstaetter J, Kickmeier-Rust M, Rösch A, Schmoldt D, Stock R, Tackenberg S, Katzky U, Konrad R, Wiemeyer J, Kruse L, Hobmeier G, Herkersdorf M, Kalbe E, Brach M, Müller-Lietzkow J, Unger T, **Martin-Niedecken, A.L.** (2018). DIN SPEC 91380:2018-06 – Serious Games Metadata Format. 1. Ed, Berlin: Beuth Verlag.

Training the body and the brain of children with cancer using an E-health tool

1. The current state of research

To prevent and counteract cognitive and physical difficulties in children with cancer, feasible, effective, appealing and sustainable interventions are needed. Currently, only few evidence-based interventions for strengthening the cognitive and physical development of children with cancer are available (Benzing et al., 2020; Braam et al., 2018; Castellino et al., 2014; Conklin et al., 2015; Hardy et al., 2013; Singh et al., 2019; for an overview see Spitzhüttl & Roebers 2020).

Our previous research has yielded valuable insights into what would be necessary and and where to specifically focus on in future interventions for children with cancer (Benzing et al., 2021). Computerized cognitive training of a core brain function can improve the trained function but fails to yield noticeable improvements of non-trained areas and no transfer occurred to academic achievements, family functioning or quality of life (Benzing et al., 2020; Everts et al., 2015, 2017; Schmidt et al., 2016). Our findings are in concordance with meta-analyses finding little evidence for a transfer effect from trained to untrained functions after computerized cognitive trainings (Melby-Lervåg & Hulme, 2013; Shipstead et al., 2012; Spencer-Smith & Klingberg, 2015). In summary, the low benefit and general lack of a transfer effect make us very hesitant to recommend existing computerized cognitive training programs to children with or recovering from cancer, despite the manifold use of such programs in daily clinical care.

Rather we believe that there are better ways to improve cognition, for instance by using cognitive strategy training. Mnemonic strategies are often used in adult neurorehabilitation in order to enhance learning and the memory after brain injury. In accordance with previous studies (Brehmer et al. 2007; Turley-Ames & Whitfield, 2003), our own investigation with children aged 7-12 years suggests that teaching mnemonic strategies not only lead to improvements in memory functions but also in untrained cognitive functions such as reading and attention in the short- and long-term (Everts & Ritter, 2017; Everts et al., 2015). During and after the acquisition of mnemonic strategies, individuals develop metacognitive thinking ("thinking about one's own thinking"). Metacognitive skills can be transferred to many tasks and can be incorporated into a variety of everyday situations (Veenman et al., 2005). In general, learning metacognitive skills and how to apply them autonomously can give individuals a sense of agency over their rehabilitation journey (Carruthers, 2012).

A recent review suggests another component to strengthen children with cancer, namely physical exercise and motor training. Physical activity is recommended from the Society for Pediatric Oncology and Hematology and supported in specific guidelines ('S2k-Leitlinie Bewegungsförderung und Bewegungstherapie in der pädiatrischen Onkologie') since such trainings improve fatigue during acute chemotherapy, strength, bone mineral density, and coordination (Coombs et al. 2012). The effect of physical exercise on cognition is particularly high if the physical tasks includes cognitive engagement (Benzing et al., 2018a; Benzing et al., 2020; Schmidt et al., 2016). Meta-analyses show that physical activities with cognitive engagement or thoughtful reflections (i.e. metacognitive thinking) support the development of executive functions, i.e. the ability to inhibit interference, routine thoughts and impulsive behaviors (ÁlvarezBueno et al., 2017; Vazou et al., 2019). Our collaborators have focused on the combined training of cognitive and motor functions in in patients with multiple sclerosis, in the elderly adults and in healthy children (see CV of Anna Niedecken-Martin) and in school children and children with ADHD (see publications of Valentin Benzing).

Derived from a review and our clinical experience, interventions, which do not require additional visits to a facility reduce participants burden, yield higher compliance rates and are highly attractive to children with cancer and their families (Brier et al., 2015). Thus, in our study the training will be delivered via a mobile e-health tool, specifically an App using a serious game (game with a purpose) - an important child-friendly aspect to increase compliance and motivation (Caserman et al., 2020; Göbel et al., 2018). A recent review demonstrates the positive effects of serious games as an intervention for paediatric cancer patients (Abdmajid et al., 2020). Furthermore, a meta-analysis comparing serious games versus conventional instruction methods revealed that serious games were more effective in terms of learning and retention than conventional instruction methods (Wouters et al., 2013).

The overall aim of our research project is to evaluate a training program that combines physical and cognitive training approaches while stimulating metacognitive abilities via a serious game. Metacognitive skills have so far been neglected in the research on intervention methods that support cognitive and physical development of children with cancer. Our new combined training approach is therefore closing a gap by incorporating cognitive tasks into motor tasks while touching upon metacognitive skills, rather than separating different training approaches as unique entities. The combination of training approaches is suggested to be the most promising approach to efficiently enhance cognitive reserve (Herold et al., 2018).

2. Applicants own research in the field

In a first cognitive training study, I have investigated the effects of two different cognitive training programs on cognitive and cerebral neuroplasticity in school-aged children born very preterm in the year 2009. This was a double-blinded, randomized, placebo-controlled, longitudinal clinical trial funded by a SNSF Ambizione grant (SNSF 126309 and 143173) and entailed several publications (Everts et al., 2017; Everts & Ritter, 2013; Mürner-Lavanchy et al., 2014a, 2014b; Ritter et al., 2013, 2014; Spencer-Smith et al., 2013). One of the two cognitive training methods under analysis was made available to the public as a training manual (Everts & Ritter, 2013, 2017). In summary, results of this trial showed that a memory strategy training had the highest impact on cognitive performance in the short- and long-term when compared to a computer-based, functional training of working memory. We explain this effect through improvements in metacognition, self-efficacy and cognitive flexibility, which were achieved with the acquisition and application of memory strategies. I have then extended my field of research by investigating neuroplasticity during and after different types of surgical and nutritional interventions in adult populations with carotid stenosis (Everts et al., 2014; Wapp et al., 2015) and phenylketonuria (Trepp et al., 2020).

During the past five years, the focus of my research has been on the training and outcome of children with cancer, among a few other projects. As a principle investigator of the Brainfit-Study (Benzing et al., 2018b), I investigated the cognitive and neural effects of physical and cognitive trainings in pediatric cancer survivors (Benzing et al., 2020). The Swiss Cancer Foundation and several other foundations financed this randomized intervention study. The findings of the BrainfitStudy suggest that children after cancer show cognitive (Siegwart et al., 2020) and physical (Benzing et al., 2021) differences to healthy controls - even years after cancer and its treatment. Further, cerebral alterations on the level of brain structure (Spitzhüttl et al., 2021) and brain function (Spitzhüttl et al., 2020; Siegwart et al., 2021) were found that likely explain the variance of the cognitive and physical problems of this population. The main finding of the Brainfit-Study was that neither a specific, computer-based, functional training of working memory nor physical activity in the framework of exergaming achieved performance increases beyond an improvement of the intensively trained cognitive function (Benzing et al., 2020).

With the set-up of a new training approach at the exercise-cognition intersection including metacognitive skills in an e-health framework, I now strive for a consequential progression and extension of my previous research. We aim to include what we have learned from our previous studies and from the studies of our research partners (Benzing et al., 2018a; Benzing & Schmidt, 2018, 2019; Martin-Niedecken, 2017, 2018; Martin-Niedecken & Götz, 2016; Martin-Niedecken & Schättin, 2020) in order to develop a new, innovative intervention method.

This project is supported by four excellent partners with complementing areas of expertise. Dr. Valentin Benzing is a Sport Scientist with special expertise in the areas of sports psychology, neuropsychology and exergames. Dr. Anna Lisa Martin-Niedecken gives her inputs as a Sports Scientist and Game Researcher with special expertise in the areas of exergames and serious games. PD Dr. Sebastian Grunt adds to the team as a Specialist in Neurorehabilitation and Prof. Dr. Jochen Roessler supports our project in his function as Head of Pediatric Oncology, Inselspital Bern. The coapplicants, Sebastian Grunt and Jochen Rössler, have permanent positions at the University Hospital
Bern with dedicated research time that will support the project. Because of the productive and stimulating cooperations with Jochen Rössler, Sebastian Grunt and Valentin Benzing in the past and the promising new collaboration with Anna Lisa Martin-Niedecken (who already collaborates with

Valentin Benzing), I am firmly convinced and very optimistic that our interdisciplinary collaboration will be prosperous, efficient and generating a lasting impact.

3. Research Plan

Background

Due to early diagnosis and improved treatment options, long-term survival rates for pediatric cancer patients have increased up to 80% (Xu et al., 2016). However, cancer and its treatments can impede the rapid development of children and adolescents, Consequently, there is a growing number of pediatric cancer survivors in need of effective long-term rehabilitation strategies. Cancer and its treatment places the child at increased risk for motor and cognitive problems (Moore, 2005), the latter often occurring in complex, higher-order cognitive domains such as executive functions (Hardy et al., 2018). Physical abilities (the capacity for a variety of movements) of children with cancer are typically impaired during and after treatment and can last for years after the treatment (Benzing et al., 2021). Diminished coordination, cardiorespiratory fitness, muscle strength and a decreased ability to perform activities of daily living have been reported (Van Brussel et al., 2006). Such physical struggles can negatively affect psychosocial development, wellbeing and quality of life (Benzing et al., 2021; Siegwart et al., 2020; Morales et al., 2018).

The tight relationship between physical abilities (e.g. motor abilities) and cognitive functions (i.e. executive functions) has increasingly been studied in healthy children and adolescents (Diamond, 2015; Donnelly et al., 2016; Hillman et al., 2008) and seems to be based on shared cognitive and neural mechanisms between motor and cognitive processes (Roebers & Kauer, 2009; Serrien et al., 2007). Consequently, studies with pediatric cancer survivors show that physical activities can have a positive effect on cognition and even on quality of life (Braam et al., 2016).

However, recent empirical evidence indicate that the type of physical exercise matters to obtain largest benefits for cognitive functions (Singh et al., 2019; Vazou et al., 2019; Ludyga et al., 2022). Pure endurance and strength training is thought to be less suitable for promoting cognitive development (Benzing et al., 2018a; Diamond & Ling, 2019; Egger et al., 2019). Instead, physical exercise that includes both, cognitive and motor demands is more likely to touch upon and thus improve cognition, such as executive functions (ÁlvarezBueno et al., 2017; Pesce, 2012; Schmidt et al., 2016; Tomporowski et al., 2015; Tomporowski & Pesce, 2019; Vazou et al., 2019). Thus, our planned study follows the strong recommendation to expose children to physical activities that combine physical effort and cognitive engagement, especially executive functions (García-Hermoso et al., 2021; Pesce et al., 2012; Herold et al., 2018).

Our previous training research revealed important findings regarding the efficacy of training interventions for pediatric cancer survivors (Benzing et al., 2021) and other clinical populations (Benzing et al., 2020; Everts et al., 2015, 2017; Schmidt et al., 2016). However, for computerized training programs compliance rates were limited, drop-out rates rather high (Benzing et al., 2020; Shipstead et al., 2012; Spencer-Smith & Klingberg, 2015) and computerized trainings were difficult to integrate into everyday family life. Not only cognitive but also physical trainings face challenges in motivating and engaging adolescents, which results in low usage, non-adherence or early drop-out (Brouwer et al., 2011) .

Serious games (game with a purpose) may help to reduce these challenges by being intrinsically motivating, highly engaging, and accessible whenever there is time. Serious games are games that are both intended for entertainment and educational purposes (Dijaouti et al., 2011) and have shown to be effective in promoting a variety of health behaviors (DeSmet et al., 2014). A recent review demonstrates the positive effects of serious games as an intervention for pediatric cancer patients and provides key guidelines that will help future game design processes for this audience. (Abdmajid et al., 2020). Furthermore, a meta-analysis comparing serious games versus conventional instruction methods revealed that serious games were more effective in terms of learning and retention than conventional instruction methods (Wouters et al., 2013).

In summary, most physical and cognitive trainings currently available for children with cancer have only limited efficacy and lack a transfer effect to other tasks, domains and especially to everyday living (Spitzhüttl & Roebers, 2020). We build up on this and will focus on a rather neglected component in the rehabilitation of children with cancer, namely metacognitive thinking.

Metacognitive thinking (”thinking about your thinking”) is the ability to supervise and manage cognitive processes (Lai, 2011) and to use knowledge to regulate behavior (Donnelly et al., 2017), cognition and physical activities. Metacognitive thinking is a cross-functional ability that has a positive effect on school success, social behavior and well-being (Veenman et al., 2005). Metacognitive thinking becomes more and more important at higher levels of education as the complexity of tasks and the responsibility for one's own performance increase. Unfortunately, metacognitive skills tend to be neglected in most rehabilitation or education programs. Helping children to improve their metacognitive skills can hold enormous benefits that are valuable across disciplines, such as skillful planning, considering alternatives, and evaluating one's own perspective. Various models exist to describe possible applications of metacognitive skills (Pintrich, 2000; Winne & Hadwin, 1998), all of them sharing the importance of engaging in a variety of processes to monitor and control performance (Zimmerman, 2001). These models often take the form of a cycle; see Figure 1 (adapted from Ambrose et al., 2010).

To implement metacognitive thinking in the framework of our training approach, we refer to the model of life skill transfer (Gould & Carson, 2008) and the identical elements theory (Thorndike & Woordworth, 1901). These models suggest that a sports skill should ideally be transferred and applied beyond sports. For example, a physical activity can be complemented with thoughtful reflections on the physical action (evaluation, planning, monitoring etc.). Through these thoughtful reflections - also referred to as metacognitive thinking - physical activity and cognitive performance can be tailored to enhance skills such as goal-setting, monitoring of behavior and self-regulation.

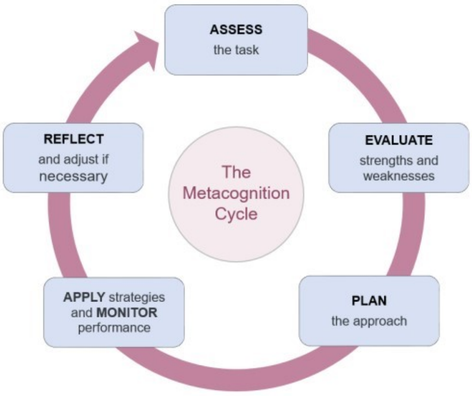

Figure 1. The metacognitive cycle

Based on the given background, we conclude that the combined training of physical and cognitive functions with metacognitive reflections can improve the training success and help to transfer the learned skills to everyday situations. We are convinced that a strengthening of metacognitive abilities can support the child's cognitive and physical development and increase school success and quality of life after cancer and its treatment.

The present project

Based on the scientific literature and our own research experience, we will develop and evaluate an ehealth application at the exercise-cognition intersection that stimulates physical abilities and executive functions in a playful manner using a serious game (game with a purpose). The aim of the random controlled trial is to evaluate effectivity of the developed training program. The training will incorporate aspects known to be crucial for the efficacy of training programs such as being adaptive, giving motivating feedback, being rich in variety, foster self-efficacy and self-confidence, including parents and caregivers, presenting training tasks, which transfer into everyday life, allowing for social exchange, stimulations with booster sessions that support long-term efficacy. For details about the training, see Figure 3 (“Cognitive and physical modules”).

Hypotheses

A) A 10-12 weeks lasting serious game intervention at the exercise-cognition intersection will lead to improved metacognitive abilities (primary outcome) and improved executive functions, motor ability, and physical self-concept (secondary outcomes) in pediatric cancer patients.

B) A positive effect of the training program on the primary and secondary outcomes will be measurable shortly after termination of the training (short-term effect). The improvement in metacognitive abilities (primary outcome) will remain at three months follow-up.

C) The serious game intervention will be perceived as acceptable, feasible, and also enjoyable by the patients and their parents.

Methods

Study Design. After the definition of needs (based on evidence-based literature and our research experience), we will develop a game-based App in an iterative user centered co-design process including prototyping, testing, refining, evaluating and finalizing. Then, we will evaluate the App for its short- and long-term efficacy in a randomized, stratified, controlled trial (see Figure 2). Children after cancer diagnosis will be randomly assigned to either the training group or the waiting control group. Randomization will be stratified according to age and sex.

Children of the training group will train for 10-12 weeks, three times per week for 20 minutes per training session, resulting in 30 – 36 training sessions in total (18 strategy training sessions, 18 physical activity sessions). Children will start to train in the inpatient clinic and will progress with the training during and after the transition back home. Children of the waiting control group will not receive any intervention for 10-12 weeks but will receive the App after the three months follow-up and can use it as long as they want. The procedure of the waiting control group is in line with the standard in clinical care where children do not receive any cognitive or physical training in the first months after the diagnosis. Investigators assessing participants at T2 and T3 will be blinded with regard to treatment assignment of the participants. Cognitive and physical assessment as well as assessment of physical self-concept will be carried out before the intervention and the waiting period (T1) and will be reperformed with all participants immediately after the intervention (T2). At 3-month follow-up only the primary outcome, namely metacognitive abilities, will be re-assed (T3; see Figure 2).

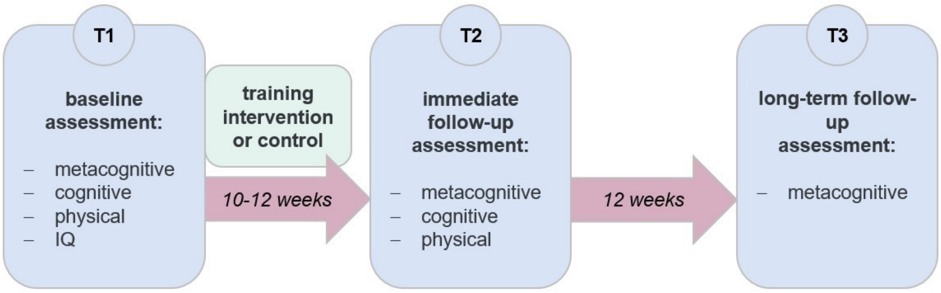

Figure 2. Study Design

Participants. We will include patients aged 8 – 12 years who are either diagnosed with cancer or patients who are undergoing cancer treatment at the University Hospital Bern Inselspital. We will start the intervention in the early phase after diagnosis or during the treatment phase. The sample size was calculated for the primary outcome measure (parent-rated metacognition index, BRIEF) that will be assessed in both groups (intervention vs. waiting control) before and after the training and waiting period and at three months follow-up. The sample size calculation was performed using G*Power 3: repeated measures ANOVA (within-between subjects interaction); small effect size (Cohen's f^2 = 0.12); α = 0.0125 (Bonferroni correction of α = 0.05 for conducting 4 tests), power = 0.8; retest correlation = 0.87 (Drechsler & Steinhausen, 2013). The resulting minimal sample size equals 22 participants per group, thus N = 44 in total. To compensate for losses and drop outs, sample size was defined at patients per group, with a total sample size of N=50.

Framework of the Training App. The theoretical framework of the App is inspired by our own training research, our experience (Benzing et al., 2018b, 2020; Everts et al., 2015, 2017) and findings from the existing literature on cognitive training (Cicerone et al., 2011) and on physical training (Benzing & Schmidt, 2018; Benzing et al., 2018a; Egger et al., 2019). Furthermore, we will follow the suggestions on web-based interventions for children with brain injuries or cancer (Abdmaji et al., 2020; Kurowski et al., 2013; Raj et al., 2018; Wade et al., 2010) and the quality criteria listed by the Swiss ehealth consortium (www.e-healthsuisse.ch).

Cognitive and Physical Modules

A) The Cognitive Training is based on the Memo-Training (Everts et al. 2017). Five mnemonic strategies will be taught and practiced in the framework of the App (inner rehearsal, chaining, visualization, finding similarities, symbolic coding). Additionally, functional training of core cognitive domains (working memory, inhibition, and cognitive flexibility) will be presented in an adaptive manner.

B) The Physical Training is based on Sphery (Martin-Niedecken et al.,2019; Martin-Niedecken & Schättin, 2020) - adapted for children and to the App format. Physical activities will be integrated playfully into the journey around the world. Tasks include cognitively challenging physical activities such as coordination, strength, endurance and physical perception.

The training of Metacognition will be integrated in A) & B) as follows: upon completion of a task, guided questions will prompt introspective investigation about changes in physical and cognitive states (i.e. "Where in my body do I feel a change now? How would I describe the feeling (warm, expanding, etc.)?"), about the evaluation of one's task performance (i.e. "How much effort did I exert for this task? How closely did I follow the instructions"?), about the recognition of one's own physical and cognitive strengths and weaknesses (i.e. "What about this task was easy for me; what was difficult?"), about future adaptations for better task performance (i.e. "What could I do to get better at this task"?) and about the planning of future tasks (i.e. "Which strategies could help me in the future?"). Such guided questions will increase the children's consciousness about their thoughts, actions and self-efficacy. Specific feedback on their performance and changes thereof will shed light on their strengths and learning strategies and will further support their 'thinking about their own thinking'.

Figure 3. Cognitive and physical modules with the integration of metacognitive thinking

The thematic framework of the serious game is planned as a journey around the world. Serious games are suggested to be especially promising when they contain a narrative, or story. Story immersion is a mechanism through which a narrative influences the childs` cognition, affect, and, potentially, health behavior. Immersion enables the story experience as a personal experience, and creates the player's affection for the narrative protagonists (Lu et al., 2012). Whether and how a player can dive into the immersion (i.e. engagement) depends on individual preferences, game control and the willingness to invest time, effort and attention during gameplay, as well as on the feedback from the game (Martin-Niedecken et al., 2017).

The details of the thematic framework will be set up in a co-creation workshop of all involved research partners. In this workshop, we will discuss practicability and technical feasibility, search for common interfaces of existing programs, focus on how to track performance of patients and adjust components of the training program accordingly. All cognitive and physical modules will be set in this journey around the world:

- Starting from the childs' geographical location, the child will travel on his/her route around the world. Several stopovers will take place around the globe: in Paris, in the Sahara, in the national parks of Botswana, in the Brasilian Rain Forest, in Californian Disneyland, on the ice of Alaska, in the heart of New York, on a snorkel tour at the Great Barrier Reef and many more.
- During the stopovers, the child will master different challenges in the form of serious games requiring cognitive and physical efforts (see cognitive and physical modules below).
- All challenges will be adaptive, hence personalized to the individual performance level of the trainee.
- The child will collect rewards for completed challenges to buy gadgets, which help to render the journey more comfortable or more adventurous (i.e. a hot-air balloon to cross the ocean, a ticket to climb the Liberty State or some gummy bears for the further journey).
- Additional tasks can be solved with higher complexity levels and higher rewards to make the game more adaptable to the individual needs of the trainees.

The children will largely be able to decide when, where and what they want to practice, which will hopefully empower them with a sense of agency over their own recovery process.

Cognitive and Physical Modules (Including Metacognitive Thinking). Since the detailed development of the physical and cognitive modules is part of the present study, we are unable to present all of the tasks in detail at this point in time. Cognitive and physical modules will be developed in an iterative process including intermediary stages (prototyping, testing, refining). Below, we present our vision of what the tasks will be like with some examples.

The Cognitive Module. Some of the cognitive tasks will be based on the principles of the well-known strategy training "Memo-Training" that will be implemented in the serious game in 12 of the training sessions. The Memo-Training, which was developed by the principle investigator in 2013 (Everts et al. 2013) was revised in 2017 and again re-revised 2022 (Everts & Ritter, 2017, to come 2022), includes a journey around the world and will therefore be easily integrated into the thematic framework of the new serious game. The Memo-Training teaches five different mnemonic strategies (so called memory tricks; see Figure 3 A) in an adaptive manner over six training sessions. In the remaining six training sessions, the mnemonic strategies will be applied in different task settings and will be practiced intensively. The level of difficulty will automatically be adapted to the child's performance level so that neither over- nor underload will occur. In addition, core cognitive functions will be trained with functional training, namely intensive practice of executive functions using adaptive games.
The training procedure is the same for all participants, with a standardized order of instructional rounds and training tasks. After the teaching of the mnemonic strategies, the child will be encouraged to apply the metacognitive cycle step-by-step (see Figure 1) to choose between the five memory strategies, to allow for autonomous memory strategy use, to evaluate and monitor the strategy and the performance and to adjust it in accordance with individual strengths and weaknesses. This will stimulate and improve the child's metacognitive abilities.

The Physical Module. The physical module will be inspired by the principles of Plunder Planet (Martin-Niedecken & Götz, 2016, 2017; Martin-Niedecken, 2017) and Sphery Racer (Martin-Niedecken & Mekler, 2018; Martin-Niedecken et al., 2019), adapted to children and to the two dimensional framework of the App. On the journey around the world, physical tasks will be implemented whenever the trainee enters the air space or the underwater world. Compared to the conventional cognitive modules, the physical modules combine the training of motor abilities with a training of specific cognitive functions. Cognitive challenges are simultaneously embedded within the physical activity and critical for successful task performance. According to the scientific literature, this seems critical to promote transfer on every day functions. There will be several options to train motor abilities, executive functions (working memory, inhibition and cognitive flexibility), attention (divided and selective attention) and processing speed during the physical tasks.
Physical activities will include:

- full-body functional movements with a focus on strength training (e.g. holding musculature, trunk musculature, and strength endurance),
- flexibility elements (e.g. stretching)
- coordination (e.g. arm-leg coordination)
- cardio-vascular training elements (e.g. high intensity interval training)
- calming and active relaxation training concepts (e.g. yoga or breathing exercises)

Participants will receive feedback on a continual basis by incorporating real-time heart rate data and cognitive performance metrics into the software's adaptive algorithms. During training, task difficulty will be adjusted trial by trial, ensuring that participants are being optimally challenged and engaged during training. The physical tasks will be adapted to the personal physical abilities and can start-off on a very low intensity level so that inpatients can train already in the clinic when lying in bed. A systematic review suggests that the individualization of a serious game to the characteristics of its players is a major contributing factor to its effectiveness (Saijadi et al., 2022); hence we follow this suggestion and make tasks adaptive. After each physical module, there will be a metacognitive cluster allowing to evaluate task performance (strength and weakness), plan how to improve the task using different strategies and reflect on the task demands and possible adjustments according to the

metacognitive cycle see Fig. 1). Metacognitive thinking comprises i.e. assessing the demands and complexity of the task at hand, planning the performance, applying and monitoring strategies, evaluating own strengths and weaknesses, and reflecting on the degree to which the used strategies are working.

Motivation, Reward & Social Interaction. Motivating feedback on the individual progress will be given continuously and rewards will be implemented after successful task completion. Rewards will constitute of coins that will allow new gadgets and accessories for the continued personalization of the avatar. The personal avatar will guide the children through the training, provide them with feedback, motivate and stimulate the children where it is most needed. Caregivers such as parents, doctors, psychologists and nurses will have access to the App and will therefore be able to follow the progress, monitor the child's cognitive and physical activities, give feedback and motivate. Further, the App will educate caregivers on how to improve metacognition in accordance with the metacognitive cycle. Advice will include suggestions for guided questions, which the caregivers can ask their children at home before and after physical exercise or cognitive activities (i.e. homework). The aim of the caregivers' involvement is a transfer of metacognitive abilities to the persons who closely monitor the child's development. Trainees will be able to interact via the app using comparing elements free of competition, i.e. comparing the development of their personalized avatars, which change in appearance.

Outcome Measures. Background variables including height, weight, and socioeconomic status (income, highest parental education, family affluence scale; Boudreau & Poulin, 2008).
The following domains will be assessed at T1 and T2:
Cognitive outcomes:

- Executive functions, namely inhibition and cognitive flexibility (Color-Word Interference Test of the Delis–Kaplan Executive Function System™, D–KEFS™; Delis et al., 2001),
- Working memory (letter-number sequencing and digit span of the German Wechsler Intelligence Scale for Children fifth edition, WISC-V; Wechsler, 2003)
- Processing speed (Coding, Cancellation, and Symbol search tests subtests of the German Wechsler Intelligence Scale for Children fifth edition, WISC-V; Wechsler, 2003).
- Executive functions, namely inhibition, cognitive flexibility and working memory (Self- and parental ratings of the German Behaviour Rating Inventory of Executive Functions, BRIEF; Drechsler & Steinhausen, 2013) Physical abilities:
- Strength and coordination (German Motor Performance Test, DMT; Bös et al., 2009).
- Physical self-concept (short version of the Physical Self-Description Questionnaire, PSDQ-S; Marsh et al., 2010)
- Physical activity behavior (Bewegungs- und Sportaktivität Fragebogen, BSA; Fuchs et al., 2015) Metacognitive abilities:
- Metacognition (Self- and parental ratings of the Cognitive Regulation Index of the German Behaviour Rating Inventory of Executive Functions, BRIEF; Drechsler & Steinhausen, 2013)

Game experience:

- Game experience questionnaire (Ijsselseijn et al. 2013)

Only at T1, IQ will be assessed as a control variable (Test of Nonverbal Intelligence, fourth edition, TONI-4; Brown et al., 2010). In addition, the acceptability, the feasibility and the enjoyment of the intervention will be assessed by means of questionnaires at T2. At T3, executive functions (BRIEF), metacognitive abilities (BRIEF) and physical self-description (PSDQ) will be assessed again.

Statistical Analysis. Raw scores of cognitive tests will be transformed to standard scores, T-scores or Intelligence quotient (IQ)- scores using age norms from the respective test manuals. Subsequently, to make results comparable among different tests, z-scores will be computed. Normally distributed background variables will be compared between the groups (training vs. control group) using two-sample t-tests and variables without normal distribution will be tested using Wilcoxon rank-sum test. Chi-squared test will be used for categorical variables. To examine training effects, primary and

secondary outcome variables of T1, T2 and T3 will be analyzed using repeated measures Analysis of Variance (ANOVA) comparing the two groups over the three time points.
Data will be stored in Redcap, which is supervised by the Clinical Trials Unit of the Faculty of Medicine of the University of Bern, Inselspital, Bern. Data will be monitored regularly. Interim data analyses will be performed by a designee, which will allow for a regular verification of the quality of the data. In case of adverse events, the following information will be collected: time of onset, duration, resolution, actions taken, assessment of the severity and of the relationship with the study intervention.

4. Time Frame of the Planned Project

Activities	Months					
	1-6	7-12	13-18	19-24	25-30	31-36
• Description of needs based on own previous research, clinical experience and the scientific literature	X					
• Development of the first app prototype<br>• Writing the application for ethical approval<br>• Preparation of the first test runs with patients	X	X				
• Pilot test the app with patients<br>• Implementation of the patients` feedback to improve the app (focus: feasibility, aesthetic appeal, compliance, motivation)		X	X			
• Conduct the random control trial.<br>• Describe the results in at least one scientific article and present the findings at national and international conferences.			X	X	X	X

5. Significance of the planned project

Research on the effects of cognitive and physical trainings in children with cancer is still in its infancy and the relationship between physical abilities and cognitive training is not yet well understood. Moreover, a core cognitive ability known to be associated with cognitive functioning, namely metacognition, has so far been neglected in the development of training programs but deserves to be included. Our game-based intervention at the exercise-cognition intersection will offer an attractive, playful, interactive and multi component tool to support physical and cognitive development in children with cancer through the stimulation of metacognitive abilities. This easily accessible, well designed, and individually adaptable training program will have a powerful impact on clinical care by contributing to prevent a decline in cognitive and physical functions during and after the cancer treatment.

An interdisciplinary research approach (merging applied and clinical science and digitalization) is necessary to develop such a program. Our collaboration among research partners will provide new insights into the best possible ways to counteract cognitive and physical long-term difficulties. Our vision is to implement the training App as a standard of care not only in Bern but also across pediatric oncology centers in Switzerland, Germany and Austria. This training program will hopefully support the cognitive and physical development of children with cancer and thereby positively affect the scholastic achievements and the well-being of this vulnerable population.

6. References

Abdmajid, E. S., Garcia, J. A., Nordin, A. I., & Raffe, W. (2020). Staying Motivated During Difficult Times: A Snapshot of Serious Games for Paediatric Cancer Patients. *IEEE Transactions on Games*.

Álvarez-Bueno, C., Pesce, C., Cavero-Redondo, I., Sánchez-López, M., Martínez-Hortelano, J. A., & Martínez-Vizcaíno, V. (2017). The Effect of Physical Activity Interventions on Children’s Cognition and Metacognition: A Systematic Review and Meta-Analysis. *Journal of the American Academy of Child and Adolescent Psychiatry*, *56*(9), 729–738.

Ambrose, S. A., Bridges, M. W., DiPietro, M., Lovett, M. C., & Norman, M. K. (2010). *How learning works: Seven researchbased principles for smart teaching*. John Wiley & Sons.

Benzing, V., Chang, Y. K., & Schmidt, M. (2018a). Acute Physical Activity Enhances Executive Functions in Children with ADHD. *Scientific Reports*, *8*(1), 1–10.

Benzing, V., Eggenberger, N., Spitzhüttl, J., Siegwart, V., Pastore-Wapp, M., Kiefer, C., Slavova, N., Grotzer, M., Heinks, T., Schmidt, M., Conzelmann, A., Steinlin, M., Everts, R., & Leibundgut, K. (2018b). The Brainfit study: efficacy of cognitive training and exergaming in pediatric cancer survivors - a randomized controlled trial. *BMC Cancer*, *18*(1), 18.

Benzing, V., & Schmidt, M. (2018). Exergaming for Children and Adolescents: Strengths, Weaknesses, Opportunities and Threats. *Journal of Clinical Medicine*, *7*(11), 422.

Benzing, V., & Schmidt, M. (2019). The effect of exergaming on executive functions in children with ADHD: a randomized clinical trial. *Scandinavian journal of medicine & science in sports*, *29*(8), 1243-1253.

Benzing, V. Siegwart, J. Spitzhüttl, J. Schmid, M. Grotzer, C. Roebers, M. Steinlin, K. Leibundgut, R. Everts, & M. S. (2021). Motor ability, physical self-concept and health-related quality of life in pediatric cancer survivors. *Cancer Medicine.*
Benzing, V., SpitzhÜttl, J., Siegwart, V., Schmid, Jü., Grotzer, M., Heinks, T., Roebers, C. M., Steinlin, M., Leibundgut, K., Schmidt, M., & Everts, R. (2020). Effects of Cognitive Training and Exergaming in Pediatric Cancer Survivors-A Randomized Clinical Trial. *Medicine and Science in Sports and Exercise*, *52*(11), 2293–2302.
Bös, K., Schlenker, L., Büsch, D., Lämmle, L., Müller, H., Oberger, J., & Tittelbach, S. *Deutscher Motorik-Test 6-18 (DMT 6-18) [German Motor Test 6-18].* Feldhaus Verlag, 2009.
Boudreau B & Poulin C. (2008). An examination of the validity of the family affluence scale II (FAS II) in a general adolescent population of Canada. Soc Indic Res.;94:29–42.
Braam, K. I., van Dijk-Lokkart, E. M., Kaspers, G. J. L., Takken, T., Huisman, J., Bierings, M. B., Merks, J. H. M., van de HeuvelEibrink, M. M., van Dulmen–den Broeder, E., & Veening, M. A. (2016). Cardiorespiratory fitness and physical activity in children with cancer. *Supportive Care in Cancer*, *24*(5), 2259–2268.
Braam, K. I., van Dijk-Lokkart, E. M., Kaspers, G. J., Takken, T., Huisman, J., Buffart, L. M., ... & van Dulmen-den Broeder, E. (2018). Effects of a combined physical and psychosocial training for children with cancer: a randomized controlled trial. BMC cancer, 18(1), 1-12.
Brehmer, Y., Li, S.C., Muller, V., von Oertzen, T., Lindenberger, U. (2007). Memory plasticity across the life span: uncovering children’s latent potential. Dev Psychol; 43:465-78.
Brier, M. J., Schwartz, L. A., & Kazak, A. E. (2015). Psychosocial, health-promotion, and neurocognitive interventions for survivors of childhood cancer: A systematic review. *Health Psychology*, *34*(2), 130–148.
Brouwer, W.; Kroeze, W.; Crutzen, R.; de Nooijer, J.; de Vries, N.K.; Brug, J.; Oenema, A. (2011). Which intervention characteristics are related to more exposure to internet-delivered healthy lifestyle promotion interventions? A systematic review. J. Med. Internet Res., 13, e2.
Brown L, Sherbenou RJ, Johnsen SK. TONI-4, test of nonverbal intelligence. Austin: Pro-ed; 2010.
Caserman, P., Hoffmann, K., Müller, P., Schaub, M., Straßburg, K., Wiemeyer, J., Bruder, R., & Göbel, S. (2020). Quality Criteria for Serious Games: Serious Part, Game Part, and Balance. *JMIR Serious Games*, *8*(3), e19037.
Castellino, S. M., Ullrich, N. J., Whelen, M. J., & Lange, B. J. (2014). Developing interventions for cancer-related cognitive dysfunction in childhood cancer survivors. *Journal of the National Cancer Institute*, *106*(8), 1–16.
Carruthers, G. (2012). A metacognitive model of the sense of agency over thoughts. *Cognitive Neuropsychiatry*, *17*(4), 291-314.
Cicerone, K. D., Langenbahn, D. M., Braden, C., Malec, J. F., Kalmar, K., Fraas, M., Felicetti, T., Laatsch, L., Harley, J. P., Bergquist, T., Azulay, J., Cantor, J., & Ashman, T. (2011). Evidence-based cognitive rehabilitation: Updated review of the literature from 2003 through 2008. *Archives of Physical Medicine and Rehabilitation*, *92*(4), 519–530.
Conklin, H. M., Ogg, R. J., Ashford, J. M., Scoggins, M. A., Zou, P., Clark, K. N., Martin-Elbahesh, K., Hardy, K. K., Merchant, T. E., Jeha,S., Huang, L., & Zhang, H. (2015). Computerized cognitive training for amelioration of cognitive late effects among childhood cancer survivors: A randomized controlled trial. *Journal of Clinical Oncology*, *33*(33), 3894–3902.
Coombs, A., Schilperoort, H., & Sargent, B. (2020). The effect of exercise and motor interventions on physical activity and motor outcomes during and after medical intervention for children and adolescents with acute lymphoblastic leukemia: A systematic review. In *Critical Reviews in Oncology/Hematology* (Vol. 152). Elsevier Ireland Ltd.
Delis D.C., Kaplan E., Kramer J.H. Delis-Kaplan executive function system (D-KEFS). San Antonio: Psychological Corporation; 2001.
DeSmet, A.; Van Ryckeghem, D.; Compernolle, S.; Baranowski, T.; Thompson, D.; Crombez, G.; Poels, K.; Van Lippevelde, W.; Bastiaensens, S.; Van Cleemput, K… (2014). A meta-analysis of serious digital games for healthy lifestyle promotion. Prev. Med., 69, 95–107
Diamond, A. (2015). Effects of Physical Exercise on Executive Functions: Going beyond Simply Moving to Moving with Thought. *Annals of Sports Medicine and Research*, *2*(1), 1011.
Diamond, A., & Ling, D. S. (2019). Aerobic-Exercise and resistance-training interventions have been among the least effective ways to improve executive functions of any method tried thus far. *Developmental Cognitive Neuroscience*, *37*(January), 1–14.
Djaouti, D.; Alvarez, J.; Jessel, J.-P. (2011). Classifying serious games: The G/P/S model. In Handbook of Research on Improving Learning and Motivation through Educational Games: Multidisciplinary Approaches; IGI Global: Hershey, PA, USA; pp. 118–136.
Donnelly, J. E., Ed, D., Co-chair, F., Hillman, C. H., Co-chair, P. D., Ph, D., Etnier, J. L., Ph, D., Lee, S., Ph, D., Tomporowski, P., Ph, D.,Lambourne, K., Ph, D., Szabo-reed, A. N., & Ph, D. (2017). Physical activity, fitness, cognitive function, and academic achievement in children: A systematic review. In *Medicine and Science in Sports and Exercise; 48*(6).
Donnelly, J. E., Hillman, C. H., Castelli, D., Etnier, J. L., Lee, S., Tomporowski, P., Lambourne, K., & Szabo-Reed, A. N. (2016). Physical Activity, Fitness, Cognitive Function, and Academic Achievement in Children: A Systematic Review. *Medicine and Science in Sports and Exercise*, *48*(6), 1197–1222.
Drechsler R. & Steinhausen H.C. Verhaltensinventar zur Beurteilung exekutiver Funktionen - German version [behavior rating inventory of executive function - German version]. Bern: Hogrefe & Huber Publishers, 2013.
Egger, F., Benzing, V., Conzelmann, A., & Schmidt, M. (2019). Boost your brain, while having a break! The effects of longtermcognitively engaging physical activity breaks on children’s executive functions and academic achievement. *PLoS ONE*, *14*(3), 1–20.
Everts, R., Mürner-Lavanchy, I., Schroth, G., & Steinlin, M. (2017). Neural change following different memory training approaches in very preterm born children–A pilot study. *Developmental Neurorehabilitation*, *20*(1), 14–24.
Everts, R., & Ritter, B. (2013). *Memo, der vergessliche Elefant* (1. Auflage). Hans Huber.
Everts, R., & Ritter, B. (2017). *Das Memo-Training: Memo, der vergessliche Elefant: mit Gedächtnistraining spielerisch zum Lernerfolg*. Hogrefe.
Everts, R., Wapp, M., Burren, Y., Kellner-Weldon, F., El-Koussy, M., Janne, K., Lenoir, J. D., Michel, P., & Schroth, G. (2014). Cognitive and emotional effects of carotid stenosis. *Swiss Medical Weekly*, *144*(July), 1–8.
Everts, R., Wapp, M., Ritter, B. C., Perrig, W., & Steinlin, M. (2015). Effects of two different memory training approaches in very pretermborn children. *Advances in Pediatric Research*.
Fuchs R, Klaperski S, Gerber M, Seelig H. (2015). Messung der Bewegungs- und Sportaktivität mit dem BSA-Fragebogen. Zeitschr Gesundheitspsychol.;23:60–76.

García-Hermoso A., Ramírez-Vélez R., Lubans D.R., et al. (2021). Effects of physical education interventions on cognition and academic performance outcomes in children and adolescents: a systematic review and meta-analysisBritish Journal of Sports Medicine;55:1224-1232.

Göbel S, Caserman P, Hansen J, Bruder R, Abels S, Behrmann M, Hauge JB, Limpach O, Reichert M, Junge J, Vogt S, Raubold O, Nowarra N, Kruse R, Roller W, Streicher A, Walter T, Wacker M, Flory C, Hugo OA, Grimm P, Dörner R… & Bergm, A. L. (2018). *DIN SPEC 91380:2018-06 – Serious Games Metadata Format*. Beuth Verlag.

Gould, D., & Carson, S. (2008). Life skills development through sport: Current status and future directions. *International Review of Sport and Exercise Psychology*, *1*(1), 58–78.

Hardy, K. K., Willard, V. W., Allen, T. M., & Bonner, M. J. (2013). Working memory training in survivors of pediatric cancer: A randomized pilot study. *Psycho-Oncology*, *22*(8), 1856–1865.

Hardy, S. J., Krull, K. R., Wefel, J. S., & Janelsins, M. (2018). Cognitive Changes in Cancer Survivors. *American Society of Clinical Oncology Educational Book. American Society of Clinical Oncology. Annual Meeting*, *38*, 795–806.

Herold, F., Hamacher, D., Schega, L., and Mueller, N. G. (2018). Thinking while Moving or Moving while Thinking–Concepts of motor-cognitive training for cognitive performance enhancement. Front. Aging Neurosci. 10:228.

Hillman, C. H., Erickson, K. I., & Kramer, A. F. (2008). Be smart, exercise your heart: exercise effects on brain and cognition. *Nature Reviews. Neuroscience*, *9*(1), 58–65.

Ijsselsteijn, W. A., de Kort, Y. A., & Poels, K. (2013). The game experience questionnaire. *Eindhoven: Technische Universiteit Eindhoven*, *46*(1).

Kurowski, B. G., Wade, S. L., Kirkwood, M. W., Brown, T. M., Stancin, T., & Taylor, H. G. (2013). Online problem-solving therapy for executive dysfunction after child traumatic brain injury. *Pediatrics*, *132*(1), e158-66.

Lai, E. R. (2011). Metacognition: A Literature Review Research Report. *Research Reports*, *April*, 41.

Lu, A. S., Baranowski, T., Thompson, D., & Buday, R. (2012). Story immersion of videogames for youth health promotion: A review of literature. *GAMES FOR HEALTH: Research, Development, and Clinical Applications*, *1*(3), 199-204.

Ludyga, S., Gerber, M., & Kamijo, K. (2022). Exercise types and working memory components during development. Trends in Cognitive Sciences, 1–13.

Marsh HW, Martin AJ, Jackson S. Introducing a short version of the physical self-description questionnaire: new strategies, short-form evaluative criteria, and applications of factor analyses. J Sport Exerc Psychol. 2010; 32:438–82.),

Martin-Niedecken, A. L. (2017). Exploring spatial experiences of children and young adolescents while playing the dual flowbased fitness game ⇜plunder planet⇝. *CHIRA 2017 - Proceedings of the International Conference on ComputerHuman Interaction Research and Applications*, *Chira*, 218–229.

Martin-Niedecken, A. L. (2018). Designing for bodily interplay: engaging with the adaptive social exertion game" plunder planet". *Proceedings of the 17th ACM Conference on Interaction Design and Children*, 19–30.

Martin-Niedecken, A. L., & Götz, U. (2016). Design and evaluation of a dynamically adaptive fitness game environment for children and young adolescents. *CHI PLAY 2016 - Proceedings of the Annual Symposium on Computer-Human Interaction in Play Companion*, 205–212.

Martin-Niedecken, A.L. & Götz, U. (2017). Go with the Dual Flow: Evaluating the Psychophysiological Adaptive Fitness Game Environment Plunder Planet - M. Alcañiz et al. (Eds.): Serious Games, LNCS 10622, Proceedings of the Joint Conference on Serious Games (JCSG '17), pp. 32-43, Springer International Publishing.

Martin-Niedecken, A. & Mekler, E. (2018). The ExerCube: Participatory Design of an Immersive Fitness Game Environment - *In book: Serious Games, Proceedings of the 4th Joint International Conference, JCSG 2018, pp.263-275.*

Martin-Niedecken, A. L., Rogers, K., Turmo Vidal, L., Mekler, E. D., & Márquez Segura, E. (2019). Exercube vs. personal trainer: evaluating a holistic, immersive, and adaptive fitness game setup. *Proceedings of the 2019 CHI Conference on Human Factors in Computing Systems*, 1–15.

Martin-Niedecken, A. L., & Schättin, A. (2020). Let the Body'n'Brain Games Begin: Toward Innovative Training Approaches in eSports Athletes. *Frontiers in Psychology*, *11*, 138.

Melby-Lervåg, M., & Hulme, C. (2013). Is working memory training effective? A meta-analytic review. *Developmental Psychology*, *49*(2), 270–291.

Moore, B. D. (2005). Neurocognitive outcomes in survivors of childhood cancer. *Journal of Pediatric Psychology*, *30*(1), 51–63.

Morales, J. S., Valenzuela, P. L., Rincón-Castanedo, C., Takken, T., Fiuza-Luces, C., Santos-Lozano, A., & Lucia, A. (2018). Exercise training in childhood cancer: A systematic review and meta-analysis of randomized controlled trials. *Cancer Treatment Reviews*, *70*(April), 154–167.

Mürner-Lavanchy, I., Ritter, B. C., Spencer-Smith, M. M., Perrig, W. J., Schroth, G., Steinlin, M., & Everts, R. (2014a). Visuospatial working memory in very preterm and term born children--impact of age and performance. *Developmental Cognitive Neuroscience*, *9*, 106–116.

Mürner-Lavanchy, I., Steinlin, M., Nelle, M., Rummel, C., Perrig, W. J., Schroth, G., & Everts, R. (2014b). Delay of cortical thinning in very preterm born children. *Early Human Development*, *90*(9), 443–450.

Pesce, C. (2012). Shifting the focus from quantitative to qualitative exercise characteristics in exercise and cognition research. *Journal of Sport and Exercise Psychology*, *34*(6), 766–786.

Pintrich, P. R. (2000). The role of goal orientation in self - regulated learning. In M. Boekaerts, P. R. Pintrich, & M. Zeider (Eds.), Handbook of self - regulation (pp. 451 – 502). San Diego Academic Press.

Raj, S. P., Narad, M. E., Salloum, R., Platt, A., Thompson, A., Baum, K. T., & Wade, S. L. (2018). Development of a Web-Based Psychosocial Intervention for Adolescent and Young Adult Survivors of Pediatric Brain Tumor. *Journal of Adolescent and Young Adult Oncology*, *7*(2), 187–195.

Ritter, B. C., Nelle, M., Perrig, W., Steinlin, M., & Everts, R. (2013). Executive functions of children born very preterm--deficit or delay? *European Journal of Pediatrics*, *172*(4), 473–483.

Ritter, B. C., Perrig, W., Steinlin, M., & Everts, R. (2014). Cognitive and behavioral aspects of executive functions in children born very preterm. *Child Neuropsychology : A Journal on Normal and Abnormal Development in Childhood and Adolescence*, *20*(2), 129–144.

Roebers, C. M., & Kauer, M. (2009). Motor and cognitive control in a normative sample of 7-year-olds. *Developmental Science*, *12*(1), 175–181.

Sajjadi, P., Ewais, A.,& De Troyer, O. (2022). Individualization in serious games: A systematic review of the literature on the aspects of the players to adapt to. *Entertainment Computing*, *41*, 100468.

Schmidt, M., Benzing, V., & Kamer, M. (2016). Classroom-based physical activity breaks and children's attention: Cognitive engagement works! *Frontiers in Psychology*, *7*(OCT), 1–13.

Serrien, D. J., Ivry, R. B., & Swinnen, S. P. (2007). The missing link between action and cognition. *Prog Neurobiol*, *82*(2), 95–107.

Shipstead, Z., Redick, T. S., & Engle, R. W. (2012). Is working memory training effective? *Psychological Bulletin*, *138*(4), 628–654.

Siegwart, V., Benzing, V., Spitzhuettl, J., Schmidt, M., Grotzer, M., Steinlin, M., Leibundgut, K., Roebers, C., & Everts, R. (2020). Cognition, psychosocial functioning, and health-related quality of life among childhood cancer survivors. *Neuropsychological Rehabilitation*, 1–24.

Siegwart, V., Steiner, L., Pastore-Wapp, M., Benzing, V., Spitzhuttl, J., Schmidt, M., ... & Everts, R. (2021). The Working Memory Network and Its Association with Working Memory Performance in Survivors of non-CNS Childhood Cancer. Developmental neuropsychology, 46(3), 249-264.

Singh, A. S., Saliasi, E., Van Den Berg, V., Uijtdewilligen, L.,... & Chinapaw, M. J. (2019). Effects of physical activity interventions on cognitive and academic performance in children and adolescents: a novel combination of a systematic review and recommendations from an expert panel. British journal of sports medicine, 53(10), 640-647.

Spencer-Smith, M., & Klingberg, T. (2015). Benefits of a working memory training program for inattention in daily life: A systematic review and meta-analysis. *PLoS ONE*, *10*(3), 1–18.

Spencer-Smith, M., Ritter, B. C., Mürner-Lavanchy, I., El-Koussy, M., Steinlin, M., & Everts, R. (2013). Age, sex, and performance influence the visuospatial working memory network in childhood. *Developmental Neuropsychology*, *38*(4), 236–255.

Spitzhüttl, J. S., Kronbichler, M., Kronbichler, L., Benzing, V., Siegwart, V., Pastore-Wapp, M., Kiefer, C., Slavova, N., Grotzer, M., Roebers, C. M., Steinlin, M., Leibundgut, K., & Everts, R. (2020). Impact of non-CNS childhood cancer on restingstate connectivity and its association with cognition. *Brain and Behavior*, *April*, 1–16.

Spitzhüttl, J.S., Kronbichler, M., Kronbichler, L., Benzing, V., Siegwart, V., Schmidt, M., Pastore-Wapp, M., Kiefer, C., Slavova, N., Grotzer, M., Steinlin, M., Roebers, C. M., Leibundgut K. & Everts R. (2021) Cortical Morphometry and Its Relationship with Cognitive Functions in Children after non-CNS Cancer, Developmental Neurorehabilitation, 24:4, 266-275

Spitzhuettl, J., & Roebers, C. M. (2020). Neuropsychological, Physical Activity and Psychological Interventions for Pediatric Cancer Survivors: A Review and Synthesis. Journal of Clinical & Developmental Psychology, 2(1).

Thorndike, E. L., & Woodworth, R. S. (1901). The influence of improvement in one mental function upon the efficiency of other functions. II. The estimation of magnitudes. Psychological Review, 8(4), 384–395.

Tomporowski, P. D., McCullick, B., Pendleton, D. M., & Pesce, C. (2015). Exercise and children's cognition: The role of exercise characteristics and a place for metacognition. *Journal of Sport and Health Science*, *4*(1), 47–55.

Tomporowski, P. D., & Pesce, C. (2019). Exercise, sports, and performance arts benefit cognition via a common process. *Psychological Bulletin*, *145*(9), 929–951.

Trepp, R., Muri, R., Muri, R., Abgottspon, S., Bosanska, L., Hochuli, M., Slotboom, J., Rummel, C., Kreis, R., Everts, R., & Everts, R. (2020). Erratum: Impact of phenylalanine on cognitive, cerebral, and neurometabolic parameters in adult patients with phenylketonuria (the PICO study): A randomized, placebo-controlled, crossover trial *21*(178)

Turley-Ames K & Whitfield M. (2003). Strategy training and working memory task performance. J Mem Lang., 49:446-68.

Van Brussel, M., Takken, T., Van Der Net, J., Engelbert, R. H. H., Bierings, M., …, & Helders, P. J. M. (2006). Physical function and fitness in long-term survivors of childhood leukaemia. *Pediatric Rehabilitation*, *3*, 267–274.

Vazou, S., Pesce, C., Lakes, K., & Smiley-Oyen, A. (2019). More than one road leads to Rome: A narrative review and metaanalysis of physical activity intervention effects on cognition in youth. International Journal of Sport and Exercise Psychology, 17(2), 153–178.

Veenman, M. V. J., Kok, R., & Blöte, A. W. (2005). The relation between intellectual and metacognitive skills in early adolescence. *Instructional Science*, *33*(3), 193–211.

Wade, S. L., Walz, N. C., Carey, J., Williams, K. M., Cass, J., Herren, L., Mark, E., & Yeates, K. O. (2010). A randomized trial of teen online problem solving for improving executive function deficits following pediatric traumatic brain injury. The Journal of Head Trauma Rehabilitation, 25(6), 409–415.

Wapp, M., Everts, R., Burren, Y., Kellner-Weldon, F., El-Koussy, M., Wiest, R., Federspiel, A., Michel, P., & Schroth, G. (2015). Cognitive improvement in patients with carotid stenosis is independent of treatment type. *Swiss Medical Weekly*.

Wechsler D. Wechsler intelligence scale for children-WISC-IV. London: Pearson; 2003.

Winne, P. H., & Hadwin, A. F. (1998). Studying as self - regulated learning. In D. Hacker, J. Dunlosky & A. Graesser (Eds.), Metacognition in educational theory and practice. Mahwah, NJ: Erlbaum.

Wouters, P., Van Nimwegen, C., Van Oostendorp, H., & Van Der Spek, E. D. (2013). A meta-analysis of the cognitive and motivational effects of serious games. Journal of educational psychology, 105(2), 249.

Xu, J., Murphy, S. L., Kochanek, K. D., & Bastian, B. A. (2016). Deaths: Final Data for 2013. *National Vital Statistics Reports: From the Centers for Disease Control and Prevention, National Center for Health Statistics, National Vital Statistics System*, *64*(2), 1–119

Zimmerman, B. J. (2001). Theories of self - regulated learning and academic achievement: An overview and analysis. In B. J. Zimmerman & D. H. Schunk (Eds.), Self - regulated learning and academic achievement (2nd ed., pp. 1 – 38). Hillsdale, NJ: Erlbaum.

Dear Reviewers,

Thank you for giving us the opportunity to submit a revised version of our project with the new title:
Training the body and the brain of children with cancer using an E-health tool.

We have completely revised our research protocol and now set a different focus on our study using the framework a random control trial. Since most of the paragraphs are new, all methods are re-written and hypothesis changes, we did not mark changes in blue color.

We had to work with the extensive comments of six Reviewers and therefore could not abide by the 6-pages constraint for the point-to-point reply. Nonetheless, we tried to keep it as short as possible and therefore only implemented the reviewer comments that include suggestions or criticism and deleted all appraising remarks. We do hope that all the favorable comments that praised our study and commended our research team (all had to be deleted due to space restriction), will not be fully forgotten when reading the point-to-point reply.

Thank you for your consideration. Kind regards,
Prof. Dr. Regula Everts

Comments from reviewer 2

Comment 1: *[…] It would have been informative to have seen a review of the already published literature to see how the authors would advance this literature. their primary innovation is the design of this game for pediatric cancer survivors. There are even video games for pediatric cancer survivors which would have been valuable to have seen a review.*
Response 1: We have now embedded the most important reviews on serious games in the Background sections (with a focus on serious games in pediatric cancer patients) and refer to the main findings of previous research. We believe that the complete revision and new set up of our research protocol is now more clearly pointing out how the present project fills the gap of research (see last paragraph of `1. The current state of research`).

Comment 2: *Involving end users in the design of an intervention, especially a game, is an important step, but there are limits on how this involvement should be structured. See A DeSmet et al. JMIR 2016, 18:e94.*
Response 2: Thank you very much for this valuable input. We carefully weighted the cost and benefits of involving the end user in the design process to a large extend (in terms of effort and time for us and the burden on the patients and their families). We finally decided to rethink the study and to minimize the participatory design process and the qualitative research approach. With regards to the App development process, only a minor participatory aspect (in the form of a small pilot study to test the design, usability etc. in an iteratiy manner; see 'Time frame' in the Research Plan, p.9) will remain. We now plan to do a randomized controlled trial (RCT), with a focus on quantitative instead of qualitative research methods (see 'Methods' section of the Research Plan, p. 5).

Comment 3: *Current issues in the design of games for health include the role of story/narrative, immersion, presence, engagement, etc. None of these issues were even cursorily addressed.*
Response 3: The role of the narrative and of immersion is now included in the method section and cited with appropriate literature (Lu et al., 2012; Martin-Niedecken et al., 2017). Furthermore, we acknowledge the importance of individualization of a serious game and follow suggestion from a systematic review on this issue (Saijadi et al., 2022) as stated in the 'Methods' of the Research Plan.

Comment 4: *The research team does not appear to incorporate someone with expertise in fun game design which could address the identified deficits.*
Response 4: Dr. Anna Lisa Martin-Niedecken is an expert on the research-based and user-centered design of both attractive and effective (serious) games for health. Furthermore, the project team will be supported by game design experts from the Subject Area in Game Design, Zürcher Hochschule der Künste. The team is well-known for their expertise in innovative game art and design for both entertainment and Serious Games.

Comment 5: *Little detail is provided on the targeted behaviors (PA or cognitive performance), game design, cognitive training, behavior change techniques, measures employed, etc. While the involvement of end users is crucial to making wise selections from among design options, the literature must be sourced to provide those alternatives.*
Response 5: We now present a random control trial and give all details the reviewer is asking for in the study protocol (see Research Plan, p. 4-9). We describe the cognitive modules in detail (based on the Memo-Training, a product of the work of Regula Everts) and present details on the physical modules

(inspired by the principles of Sphery Racer and Plunder planet, both products of the work of our collaborator Anna Lisa Martin-Niedecken). We further describe the methods in much more detail and present an approach how to teach and train metacognitive thinking.

Comment 6: *A sample of 20 will not be able to reliably or validly inform answers to questions III b, c, or d. A sense of feasibility is the best that could be obtained.*
Response 6: We now reframed the project to a randomized controlled trial and performed a power analysis to determine the number of patients needed for our primary outcome analysis (see 'Participants' in the Method section). We now aim to include 40 patients (20 per intervention group).

Comment 7: *Dr. Anna Lisa Martin-Niedecken, M.A. appears to have a very similar grant funded: 2021 – 2023: AAL Grant (1'181'193.30 €), Project: "ExerG: An innovative digital solution to individually improve physical and cognitive functions using an exergame (video game-based) training in an ecologically valid and safe setting for the geriatric population". This was not mentioned in the application. How do they differ?*
Response 7: This project is indeed also focusing on the development of a concurrent training of physical and cognitive functions by improving walking, balance, and cognitive abilities of older adults in a video game-based training device (ExerG). The training is performed by integrating a body weight support system. Players control a video game environment, projected on three surrounding screens, with specific (whole) body movements. The expertise from this project will flow into our project. However, the whole framework of our planned App, its metacognitive focus and our end user population clearly differ from the AAL grant mentioned above

Comments from reviewer 4

Comment 1: *[…] In addition, the authors take into account the question of acceptability: a holistic approach will be applied involving patients and their primary care givers via a smartphone app (personalized serious game involvingcognitive and motor modules as well as psychoeducation).*
The research method is rigourous and in adequacy with the aim of the study. […]
If the research plan seems to be very solid, we regret that the applicant give no details about how they plan to analyze quantitative (statistic plan?) and qualitative data (i.e. Focus groups).
Response 1: We have now re-designed the study to a randomized controlled trial and we give details on the methodology and the statistical plan (see Research Protocol p.9). However, upon careful consideration, we now refrain from the qualitative approach and focus on a purely quantitative research design.

Comment 2: *The applicant has extensive experience in the field, the previous research projects are of high quality, which allows us to be confident about the success of the project.*
Response 2: Thank you very much for your confidence in our project plan.

Comments from reviewer 5

Comment 1: *Originality and innovative aspects […] This program target patients in the course of treatment and after treatment. There is an objective of both remediation/rehabilitation and prevention, which is also a strength of the program.*
Response 1: Thank you for this positive feedback and for your support.

Comment 2: *The project includes 3 phases, which is advisable to set up interventions.*
[…] However, the methodology as detailed does not seem suitable for evaluation of efficacy of the program. This extremely important part is not well developed: aim, evaluation tools, and evaluation times, statistical analyses are not or not well detailed (see comments below for major comments).
Response 2: After thorough discussions within our study group, we decided to change our research approach to a purely quantitative method while omitting the qualitative research aspects. We realize that in order to have an evidence-based product supporting rehabilitation after childhood cancer, the focus of the project should be the evaluation of the training program, not the development itself. Hence, we now present a randomized controlled trial (RCT) using quantitative research methods. As the PI of the project, I feel more confident with this approach and can build upon my experience from previous RCT. The participatory design approach might be useful for some clinical populations, however in children diagnosed with cancer, the burden to take an active part in a participatory design project in addition to coping with the disease and its treatment's side effects might exceed the resources of families and patients.

Comment 3: *[…]The duration of 36 months and the request for funding are justified and appropriate to the assessment of needs, the development of the application and a pilot phase of evaluation (acceptance, compliance...). If the project includes an efficacy/effectiveness evaluation, this phase will need to be better described and the study will probably require more time.*

Response 3: The evaluation of the efficacy of the training program is now the focus of the research project and is therefore described in much more detail in the Research Protocol (p.4 – 9). Because we decided to significantly reduce the participatory aspect of the design process and to entirely omit the qualitative methodology of the research project, we now have at least 24 months for the evaluation of the efficacy of the training program (see 'Time frame' in the Research Protocol, p. 9).

Comment 4: *[…] The constitution of the team meets the needs of the project.*
Response 4: Thank you for this positive evaluation. In the meantime, our research team even strengthened its collaboration as we reflected thoroughly on the reviewers' comments, obtained advice from others and discussed new issues with respect to the study protocol within the team. Hence, I am more convinced than ever, that our research proposal will be successful.

Comment 5: *Regarding the development of the program:*
The program targets children aged between 8 and 18 with different types of cancer at different times oftreatment. This can be a strength (see above) but also a limiting point:
a) *How do the authors justify this point? The inclusion criteria should be more detailed.*
b) *Depending on the age of children, physical and motor capacities, centers of interest will be different. Do the authors plan to adapt the application / game according to the age?*
c) *Similarly, depending on the type of cancer, the difficulties and needs of children may not be quite the same.*
d) *Will the authors assess the needs of the child and his family before their participation? Are adaptations of the serious game modules planned according to their needs / pathologies?*

Response 5: regarding point (1)
a) Inclusion criteria are now more clearly specified and a power analysis justifies the new sample size of n = 40 (see 'Participants' in the Research Protocol, p.5).
b) Since the age range is now narrower in the revised version of the Research Protocol (8-12 years), we do not adapt the training modules for age. However, all tasks will be adaptable to the individual performance level in order to avoid under- or over challenge.
c) The literature shows that children with brain tumors show more alterations in cognitive and physical development than children with other cancer types (Margelisch et al. 2015). Furthermore it is suggested, that a combination of cancer treatments (i.e. radiotherapy and chemotherapy) entails more pronounced cognitive alterations than surgery alone (i.e. Anderson et al. 1997). However, cognitive and physical development is at risk in all types of cancer and after all treatment types despite varying degrees of impact. Further, more factors besides the cancer type and treatment can impact the individual recovery needs. This is why we do not adapt the training program to the different cancer or treatment types. Within the App, all tasks are adaptive in difficulty, hence individualized to the individual needs and of the children.
d) We have some knowledge from two reviews focusing on the individual needs of children with cancer and pediatric cancer survivors (Brier et al., 2015; Raj et al., 2018). This knowledge will be crucial for the development phase of the App. Furthermore, we have extensive experience and data from our own previous interviews with inpatients (unpublished) about the needs of our patients with cancer. All of these considerations will be embedded in the App.

Comment 6:*The intervention methods must be specified:*
a) Duration and frequency of tasks / modules of the serious game.
b) Need to "play" every day? Indeed, the capacities of the child (physical / cognitive) can also vary according to the phases of the treatment. During the intensive phases, children and parents could be less available.
c) How will parents be involved? This point deserves to be more detailed.

Response 6: Because our revised version of the study protocol now focuses on a random control trial, we describe the study design in much more detail so that question a to c is answered (see 'Methods' of the Research Plan, p. 4 – 8).

Comment 7: *Regarding the assessment of the program:*
The phase 3 needs more precision. The assessment of feasibility and acceptability, the assessment of the patients' experience and their motivation seems appropriate even if these points deserve to be more detailed (question a). A mixed approach is planned (quantitative and qualitative data) which is recommended. However if authors want assess the efficacy of the program and the factors related to the success of the intervention (questions b, c and d), they must clarify certain points:
- *The calculation of the sample size needs to be better supported. Given the number of variables and the heterogeneity of the population, it is important to detail this point. The number of patients envisaged (n=20) should allow answering the question "a" but will not answer to the others.*

- *Evaluations time, tools and questionnaires used in function of the age of the patients,*
- *Cognitive and motor tests should be detailed.*
- *Is it planned to form a control group?*
- *The statistical analysis plan should be detailed.*

Response 7: As described above, the new study design is now presented in much more detail in the Research Protocol (p. 4 – 9). A power analysis is presented based on the primary outcome suggesting a sample size of n= 40, outcome measures are clearly described, tests are presented and cited in detail, the control group is described and the statistical analysis plan has been adjusted to the new random control trial.

Comment 8: *Minor comments: Social and psychological difficulties are also frequent during and after treatment and associated with cognitive and physical difficulties. Additionally, children's emotional state may play a role in adherence, motivation, and compliance with interventions.*
Do the authors plan to assess the emotional state of children and their parents? Will the serious game include specific modules dedicated to these issues?
Response 8: There is no focus on the emotional state and psychosocial modules are not included in the App. In everyday clinical care, a psychologist accompanies all patients and their families during their time at the hospital and during treatment and aftercare, hence the emotional state of the patient is closely supervised. The app will be introduced to the patients and their families by a healthcare professional, who knows the family well and will be in regular contact with the family throughout the study. This healthcare professional will assess the patient's and their family's individual circumstance and decide with them together, whether the training program is a good fit for them in their situation at that time.

Comments from reviewer 6

Comment 1: *[…] However, the intervention methods need to be specified (eg duration and frequency of tasks...). Some current issues in the design of games for health could also have been specified (the role of story/narrative, immersion...). The program targets children aged between 8 and 18 with different types of cancer at different times of treatment.*
This is potentially a limiting point and has to be justified, since the centers of interest and the needs can be rather different.
Response 1: We have developed the intervention method in much more detail now (see 'Methods' section in the Research Plan, p. 6-8). In the Research Plan, we present tasks that will be incorporated. The individual tasks will have different durations. For the study, children of the training group will train for 10-12 weeks, three times per week for 20 minutes per training session, resulting in 30 – 36 training sessions in total (18 strategy-training sessions; 18 physical activity sessions).
We agree with the feedback on the difficulty of designing an app that is attractive to a broad age range. After careful reconsideration, we have limited the range to children from 8 – 12 years of age. We believe, however, that the app will be well suited for patients with different types of cancer, since the difficulty levels of the tasks are individually adaptable. Further, there will be a variety of tasks, catering to different levels of physical mobility of the patient (i.e. some tasks can be done lying in bed).

Comment 2: *[…] The methodological aspects (aim, evaluation tools, and evaluation times, statistical analyses) are insufficiently described. Moreover, the low number of patients (20) will not be sufficient to answer questions III b, c, or d.*
The feasibility of the development of the application is excellent, regarding the experience of the team. The applicant and co-applicants team have already worked and published together. Some data have already beencollected. However, the phase 3 objectives appear difficult to be reached (see above).
Response 2: We agree with this feedback and thus, we have allocated much more time to conducting the feasibility study (see 'Time frame' in the Research Plan, p. 11). In addition, we have reduced the research questions that we will try to answer with the study. The focus will now be on the evaluation of the training program and on its efficacy with improving the metacognition scores. The analysis of the mechanisms associated with its success will not be an aim of this study anymore and could instead be addressed in a larger, future study.

Comments from reviewer 7

Comment 1: *[…] The authors plan to have three phases: the first dedicated to assessments of needs with quantitative and qualitative evaluation, the second dedicated to the development of intervention end the third dedicated to theevaluation of the efficacy of intervention. A sample of 20 will not be able to reliably or validly inform answers to questions III b, c, or d. A sense of feasibility is the best that could be obtained.*
Response 1: We agree with this feedback and thus, we have allocated much more time to conducting the feasibility study (see 'Time frame' in the Research Plan, p. 11). In addition, we have reduced the research questions that we will try to answer with the study. The focus will now be on the evaluation of the program

with improving the metacognition scores. The analysis of the mechanisms associated with its benefit will not be an aim of this study anymore and could instead be addressed in a larger, future studies.

Comment 2: *The inclusion criteria should be more detailed. Depending on the age of children, physical and motor capacities, centers of interest will be different. Do the authors plan to adapt the application / game according to the age? depending on the type of cancer, the difficulties and needs of children may not be quite the same. Will the authors assess the needs of the child and his family before their participation? Are adaptations of the seriousgame modules planned according to their needs / pathologies?*
Response 2: The literature shows that children with brain tumors show more alterations in cognitive and physical development than children with other cancer types (Margelisch et al. 2015). Furthermore the literature suggests, that a combination of cancer treatments (i.e. radiotherapy and chemotherapy) entails more pronounced cognitive alterations than surgery alone (i.e. Anderson et al. 1997). However, cognitive and physical development is at risk in all types of cancer and after all treatment types despite varying degrees of impact. Further, more factors besides the cancer type and treatment can affect the individual recovery needs. This is why we do not adapt the training program to the different cancer or treatment types. Within the App, all tasks are adaptive in diffculty, hence individualized to the individual needs and of the children. We have some knowledge from two reviews focusing on the individual needs of children with cancer and pediatric cancer survivors (Brier et al., 2015; Raj et al., 2018). This knowledge will be crucial for the development phase of the App. Furthermore, we have extensive experience and datafrom our own previous interviews with inpatients (unpublished) about the needs of our patients with cancer. All of these considerations will be embedded in the App.

Comment 3: *The intervention methods must be specified: Duration and frequency of tasks / modules of the serious game. Need to "play" every day? Indeed, the capacities of the child (physical / cognitive) can also vary according to the phases of the treatment. During the intensive phases, children and parents could be less available. How will parents be involved? This point deserves to be more detailed*.
Response 3: We now have planned the intervention methods in much more detail as laid out in the new Research Plan (p. 4 – 9). For instance, “participants will train for 10-12 weeks, three times per week for 20 minutes per training session, resulting in 30 – 36 training sessions in total (18 strategy training sessions, 18 physical activity sessions)” (p. 4). The app will include will include tasks on different scales of difficulty and requiring different levels of mobility in order to adapt for the huge variety in needs and capabilities of patients in different phases of the treatment. For example, the physical module will encompass tasks for many different levels of physical mobility (i.e. some tasks can be done lying in bed).

Comment 4: *The calculation of the sample size needs to be better supported. Given the number of variables and the heterogeneity of the population, it is important to detail this point. The number of patients envisaged (n=20) should allow answering the question “a” but will not answer to the others*.
Response 4: We have since conducted a new sample size calculation (see ‘Participants’ in the Research Protocol, p. 5 – 6) and plan to include n = 40 patients. Indeed, this sample size only allows for investigating the main hypotheses, namely showing if the training program improves outcomes in a group comparison over the three time points. The investigation of potential factors, which could influence the success of the training, indeed goes beyond the scope of the planned study and hence is omitted from the hypothesis. Thus, this specific investigation will be purely exploratory with the aim of inspiring future studies on this topic.

Comment 5: *Evaluations time, tools and questionnaires used in function of the age of the patients, cognitive and motor tests should be detailed. The statistical analysis plan should be detailed.*
Response 5: The new Research Protocol provides details on the outcome measures (p.8 - 9) and on the statistical analysis (p. 9).

Comment 6: *[…] The research team does not appear to incorporate someone with expertise in fun game design which could address the identified deficits.*
The co-applicant have research activities and publication record focus on links between physical activity andcognitive development, cognition and pediatric cancer, rehabilitation after pediatric cancer, development of esport / e-health… Anna Lisa Martin-Niedecken has also a great experience on development of fitness videogame and actually works at the head of institute of design research. The constitution of the team meets the needs of the project.
Response 6: Dr. Anna Lisa Martin-Niedecken is an expert on the research-based and user-centered design of both attractive and effective (serious) games for health. Furthermore, the project team will be supported by game design experts from the Subject Area ‘Game Design’, Zürcher Hochschule der Künste.

The team is well-known for their expertise in innovative game art and design for both entertainment and Serious Games.

Comments from reviewer 8

Comment 1: *[…] Please consider the approaches from Barry: Barry, G., Galna, B., & Rochester, L. (2014). The role of exergaming in Parkinson's disease rehabilitation: a systematic review of the evidence. Journal of neuroengineering and rehabilitation, 11(1), 1. It is not in the field of cancer, but also addresses the combination and potential of exergaming in terms of a combination of physical exercises and cognition.*
For the game-based approach and therapy adherence, the work from P. Kato et.al in the context of Re-Mission (cancer therapy for young people) might be considered as well. This example also underlines the strong involvement (and required budget) of/for game developers, who are responsible for the development of the app.
Response 1: Thank you for the interesting literature suggestions. The systematic review by Barry et al. (2014) suggests that the methodology of studies overall is rather weak and that there is a lack in randomized controlled trials (RCT) testing computerized exergames for rehabilitation purposes. Thus, we feel even more encouraged in our decision to redesign the project and focus on a RCT. We also appreciate the reminder to allocate sufficient financial means and time of the project to the professional game developers.

Comment 2: *Research plan and development: The project aims to develop an app with a story(telling) component which is going to be considered in the whole therapy program. This storytelling aspect sounds fine in principle, but might become very complex in the development phase, especially when personalised approaches are considered: this would require adaptive storytelling techniques (with 'adaptive content'), which would be a complex project on its own.*
Response 2: We appreciate this attention to the details of the design. After careful considerations, we are confident that we came up with a good compromise in the storyline between a general framework, which is not too complex, and individually adaptable elements. Specifically, the framework of a journey around the world leaves a lot of room for individual decisions, such as in which order to 'visit' different places (see 'framework of the training app' in the Research plan p. 6 – 7).

Comment 3: *Evaluation/testing: Some references are provided referring to evaluation. Apart from UX, game experience aspects also should be taken into account. Here, the work from Ijsselstein providing a game experience questionnaire should be reflected. Besides, as stated in the proposal, it is clear that the duration of the project (36 month) only allows evaluation aspects in terms of a feasibility study and getting a rough idea about usability, UX, GX, motivational aspects... A mid/longterm clinical study would be necessary to validate clinical aspects.*
Response 3: We thank the reviewer for this valuable suggestion to include a game experience questionnaire, which is now part of our outcome measures. The evaluation of the efficacy of the training program is now the focus of the research project and is therefore described in much more detail in the Research Protocol (p.4 – 9).
